# Paradoxical relationships between active transport and global protein distributions in neurons

**DOI:** 10.1016/j.bpj.2021.02.048

**Published:** 2021-04-02

**Authors:** Adriano Bellotti, Jonathan Murphy, Lin Lin, Ronald Petralia, Ya-Xian Wang, Dax Hoffman, Timothy O’Leary

**Affiliations:** 1 National Institute of Child Health and Human Development, Bethesda, Maryland; 2 National Institute on Deafness and Other Communication Disorders, Bethesda, Maryland; 3 Department of Engineering, University of Cambridge, Cambridge, United Kingdom

## Abstract

Neural function depends on continual synthesis and targeted trafficking of intracellular components, including ion channel proteins. Many kinds of ion channels are trafficked over long distances to specific cellular compartments. This raises the question of whether cargo is directed with high specificity during transit or whether cargo is distributed widely and sequestered at specific sites. We addressed this question by experimentally measuring transport and expression densities of Kv4.2, a voltage-gated transient potassium channel that exhibits a specific dendritic expression that increases with distance from the soma and little or no functional expression in axons. In over 500 h of quantitative live imaging, we found substantially higher densities of actively transported Kv4.2 subunits in axons as opposed to dendrites. This paradoxical relationship between functional expression and traffic density supports a model—commonly known as the sushi belt model—in which trafficking specificity is relatively low and active sequestration occurs in compartments where cargo is expressed. In further support of this model, we find that kinetics of active transport differs qualitatively between axons and dendrites, with axons exhibiting strong superdiffusivity, whereas dendritic transport resembles a weakly directed random walk, promoting mixing and opportunity for sequestration. Finally, we use our data to constrain a compartmental reaction-diffusion model that can recapitulate the known Kv4.2 density profile. Together, our results show how nontrivial expression patterns can be maintained over long distances with a relatively simple trafficking mechanism and how the hallmarks of a global trafficking mechanism can be revealed in the kinetics and density of cargo.

## Significance

Healthy nervous system function depends on continuous replenishment of proteins across complex neuronal morphologies. We provide experimental evidence that an ion channel important for signal integration and regulation of excitability maintains a specific intracellular expression pattern with a relatively simple trafficking mechanism in which cargo is distributed widely and sequestered where needed. This model accounts for substantially different kinetics of cargo movement in different parts of the cell and, paradoxically, predicts that trafficked cargo can be denser in compartments where it is not used. This provides new data on global protein trafficking and insights into the mechanisms underlying expression patterns important for neuronal function. Our results also prompt caution on how to interpret static and dynamic intracellular cargo measurements generally.

## Introduction

Neurons homeostatically maintain function by continually producing proteins and distributing them to function-specific regions of the cell. The logistics of this task are especially challenging in complex neural morphologies with projections that extend hundreds to thousands of microns (1, 2, 3). Most proteins have half-lives on the order of hours (4), and some proteins have very precise distributions that are important for cell physiology. Although many proteins, particularly small soluble proteins, are synthesized locally in dendrites (5, 6, 7), others are primarily synthesized at or near the soma. Among these are many kinds of ion channels that control neuronal excitability and whose intracellular spatial distribution is tightly regulated (8).

How do intracellular trafficking mechanisms maintain spatial distributions of protein in a complex morphology? This question is critical for our understanding of neuronal biophysics and homeostasis and for unraveling the causes of pathologies associated with dysregulation of protein expression (9,10). Leading conceptual models suggest that cargo is exported and sorted, and local interactions detect and sequester bypassing subunits as needed (11, 12, 13, 14). This general model, in which cargo is distributed coarsely at a global level and local interactions dictate fine-grained subcellular distribution, is called the sushi belt model (12). However, the extent to which this model explains ion channel distributions in neurons remains open.

In this study, we directly test whether the sushi belt model can account for relationships between ion channel traffic and steady-state distributions of a voltage-gated potassium channel, Kv4.2, whose subcellular distribution is critical for maintaining cellular excitability (8,15,16). Kv4.2 conducts an A-type, transient potassium current, which is abundant in dendrites but scarce in axons (15). Dendritic expression of Kv4.2 is consistent with its hypothesized role in dendritic integration and control of excitability (8,15,16). Functionally, the current density exhibits a five- to sixfold increase along the length of the apical dendrite (16). Localization studies of Kv4.2 corroborate this finding, showing a 70% increase in channel density along the apical dendrite, from soma to the apical region (17).

Axonal Kv4.2-mediated A-currents have not been reported, but other channels that pass A-current have been found in axons (15,18,19). The reported amount of Kv4.2 subunits localized in axons varies substantially among quantitative localization studies. Alfaro-Ruíz et al. report only 1.2% of total CA1 immunogold particles are found in axons (20). Kerti et al. contrastingly report nearly 20%, and the authors remark that “[this result] is surprising, because the Kv4.2 subunit is conceived as a somato-dendritic ion channel” (17). In this study, we recapitulate these results and found predominant expression of Kv4.2 in dendrites with a non-negligible presence in axons. Several studies have measured Kv4.2 trafficking and internalization in dendrites (21, 22, 23, 24), but none to date have enabled a quantitative, global model of transport and expression patterns.

We measured and analyzed Kv4.2 active transport in axons and dendrites, including displacement, directional bias, speed, and stall time of individual particles. We inferred parameters in a stochastic model of transport that accounts for cargo dynamics, indicating qualitative differences between axonal and dendritic transport. Axonal transport exhibited superdiffusivity, with long, uninterrupted runs, consistent with lower subunit demand and fewer interactions. By contrast, dendritic cargo trajectories were diffusive, consistent with increased local interactions that interrupt transport.

Surprisingly, we found a greater density of actively transported Kv4.2 subunits in axons than in dendrites. We show that the apparent discordance of high trafficking densities in regions of low functional expression turns out to be consistent with a simple lumped model of intracellular transport derived from the sushi belt model. Furthermore, a spatially discretized version of the model accounts for increasing Kv4.2 localization from proximal to distal compartments. This expression pattern has been extensively characterized and is important for dendritic function (16,25,26), but the question of how it emerges from a relatively simple trafficking mechanism has remained unanswered. We experimentally estimated model parameters including microtubule occupancy and transport rates as functions of distance from soma. Constrained with these data, we provide an analytical solution for the microtubule occupancy profile that can recapitulate the Kv4.2 localization profile along dendrites.

Together, our findings constitute a test of a widely hypothesized, parsimonious model of intracellular transport. We find that this model is consistent with measured and highly specific intracellular protein distributions and predicts observed disparities between transported and delivered cargo.

## Materials and methods

### Animals and cell culture

All animal procedures are conducted with accordance of the National Institutes of Health Guide for the Care and Use of Laboratory Animals under a protocol approved by the National Institutes of Child Health and Human Development (NICHD)’s Animal Care and Use Committee.

#### Rat hippocampal dispersed cultures

Hippocampal cultures are prepared from gestational day 18 to 19 wild-type (WT) Sprague-Dawley rats as previously described (23). Briefly, fetal pups are removed from the mother, and hippocampus tissues are dissected and placed in dissection media. For 500 mL of dissection media, we filter sterilized 50 mL 10× Hanks' Balanced Salt Solution (HBSS) (14185-052; Gibco, Gaithersburg, MD), 5 mL penicillin/streptomycin (15140122; Gibco), 5 mL pyruvate (11360070; Gibco), 5 mL HEPES (1 M, 15630080; Gibco), 15 mL of 1 M stock solution glucose (from powder; Sigma-Aldrich, St. Louis, MO), and 420 mL Ultra Pure Water (KD Medical, Columbia, MD).

Tissue was mixed with papain (Worthington Biochemical, Lakewood, NJ) for 45 min at room temperature. Tissues were rinsed for removal of extracellular material with dissection media several times, and dissociated cells were plated in neurobasal media (Thermo Fisher Scientific, Waltham, MA) with 5% fetal bovine serum (HyClone characterized fetal bovine serum, SH30071.03; GE Healthcare LifeSciences, Pittsburgh, PA), 2% GlutaMAX (Thermo Fisher Scientific), and 2% Gibco B-27 supplement (Thermo Fisher Scientific) (subsequently called NB5 media). Cells were incubated in 5% CO_2_ at 37°C. After 24 h, cells were transferred to neurobasal media containing 2% GlutaMAX and 2% Gibco B-27 supplement (NB0 media). Half of the media is replaced with fresh NB0 media every 3–4 days, and cells are imaged after 9–13 days in vitro.

#### Construct

A Kv4.2 construct was conjugated at the N-terminus to strongly enhanced green fluorescent protein (SGFP2) (27), henceforth referred to as Kv4.2-SGFP2. pSGFP2-C1 was a gift from Dorus Gadella (plasmid # 22881; Addgene, Watertown, MA; http://www.addgene.org/22881/; RRID:Addgene_22881). We subcloned mouse Kv4.2 into the SGFP2 plasmid using NheI amd SalI restriction sites.

#### Transfection

Lipofectamine 2000 transfection was performed following manufacturer protocol with some modifications. 2 *μ*L of Lipofectamine 2000 Transfection Reagent (Thermo Fisher Scientific) and 2 *μ*g of DNA plasmid were each diluted in 200 *μ*L of neurobasal media and incubated at room temperature for 5 min. The two solutions were then combined and incubated at room temperature for 15–20 min. 100 *μ*L of total mixture was added to each well and incubated at 37°C for 4 h before changing media. The cells were then incubated for an additional minimum of 1 h before imaging.

#### Immunostaining

After hour-long time series, samples reserved for antibody staining were fixed or permeabilized and immunostained as previously described (28,29) and briefly reiterated here. Upon completion of time series, the coverslips were removed from the imaging chamber, and the location of the neuron of interest was labeled with a fine tip marker. Coverslips were fixed with 4% paraformaldehyde (R 15710; Electron Microscopy Sciences, Hatfield, PA) and 4% sucrose (S9378; Sigma-Aldrich) at room temperature for 15 min, followed by three 1× PBS (14190; Gibco) washes before overnight storage in 1× PBS at 4°C. Coverslips were permeabilized in 0.2% Triton X-100 (T8787; Sigma-Aldrich) for 5 min at room temperature and washed once in 1× PBS for 5 min. Cells were incubated for 1 h at room temperature in 0.04% Triton X-100 solution in 1× PBS containing 1:100 dilution of anti-ankyrin-G rabbit primary antibody (75–146; NeuroMab, Davis, CA) or 1:1000 dilution of MAP2 antibody (Chemicon, Burlington, MA). Upon the completion of primary incubation, coverslips are washed three times with 1× PBS for 5 min. Coverslips are then incubated with secondary antibodies anti-rabbit-555 (1:500) for ankyrin-G or MAP2 and anti-GFP-488 (1:400) (Molecular Probes, Eugene, OR) for 1 h at room temperature before another three washes with 1× PBS. Coverslips were then mounted onto glass slides using ProLong Diamond Antifade Mountant containing DAPI (Invitrogen, Carlsbad, CA).

### Microscopy

In this study, we relate experimental observations to microtubule-bound cargo density (*mt*), delivered cargo density (*del*), and total cargo density (*mt* + *del*). These observations are made using various modes of microscopy and are intended as comparative measures of density between axons and dendrites. Election microscopy of synapses reveals *del* cargo. Fluorescence microscopy of transfected Kv4.2-SGFP2 and immunostaining of endogenous Kv4.2 both represent total *mt* + *del* cargo. Time series recordings of mobile puncta reveal *mt* cargo.

#### Hour-long time series recordings

18-mm coverslips were removed from wells and placed in a Quick Release Chamber (QR-41LP, 64–1944; Warner Instruments, Hamden, CT). Cells were immersed in 800 *μ*L imaging buffer consisting of 1× Tyrode’s solution: 135 mM NaCl, 5 mM KCl, 2 mM CaCl_2_, 1 mM MgCl_2_, 25 mM HEPES, and 10 mM glucose (all Sigma-Aldrich) at pH 7.4. All imaging was carried out at the NICHD Microscopy and Imaging Core using a Zeiss LSM 710 laser scanning confocal microscope (Carl Zeiss Microscopy, White Plains, NY). Global still images were captured using a 40× oil-immersion objective and stitched together in ImageJ.

Time series were performed using a 63× oil-immersion objective with the pinhole diameter set to 4 airy units. The 495-nm laser line was used for both imaging and bleaching. During imaging, the laser power was set to 4%, and during bleaching, the power was set to 100%. Images were acquired at 1024 × 1024 resolution at 1.0× optical zoom with 750× gain. Time-lapse images were captured at 0.2 Hz for 60–85 min using Zeiss LSM Image Browser software. Z-plane focus was maintained using Zeiss Definite Focus after each frame captured. The cells were temperature- and CO_2_-controlled at 37°C and 5% during imaging using a stage top incubator (STXG-WSKMX-SET; Tokai Hit, Fujinomiya, Japan). Every 10–20 min, Kv4.2-SGFP2 was bleached to 30–70% of baseline intensity by the same 495-nm laser (100% power, 10 iterations) over the bleach region of interest.

#### Extended time series

Coverslips were maintained for extended recordings of up to 11 h. A distilled water reservoir was placed adjacent to the imaging chamber, and the chamber was covered with a 35-mm petri dish to maintain humidity. Time-lapse images were captured at 0.1 Hz with recurrent photobleaching every 50 min. All other procedures and conditions are as previously described.

#### Colchicine treatment

Samples treated with colchicine (C9754; Sigma-Aldrich) were prepared and time series captured as previously described for hour-long recordings. Six coverslips were treated with colchicine during hour-long recordings. At 30 min into the recording, 1 *μ*L dimethyl sulfoxide (DMSO) (control) or 1 *μ*L DMSO containing 40 *μ*g colchicine was mixed into the imaging chamber, for a final colchicine concentration of 125 *μ*M. The number of mobile puncta per minute was then counted for the duration preceding administration and starting 10 min postadministration.

#### Electron microscopy

The electron micrographs used in this study were collected for a previous study (30). Mouse hippocampi used for the postembedding immunogold localization were prepared as described previously (31, 32, 33). Mice were perfused with phosphate buffer, followed by perfusion with 4% paraformaldehyde + 0.5% glutaraldehyde in phosphate buffer. Fixed brains were vibratomed at 350 *μ*m, then cryoprotected in glycerol overnight and frozen in a Leica EM CPC (Leica Microsystems, Wetzlar, Germany), and processed and embedded in Lowicryl HM-20 resin (Electron Microscopy Sciences) in a Leica AFS freeze-substitution instrument. Thin sections were incubated in 0.1% sodium borohydride + 50 mM glycine in Tris-buffered saline plus 0.1% Triton X-100 (TBST). Then they were immersed in 10% normal goat serum (NGS) in TBST, and primary antibody in 1% NGS/TBST (overnight), then incubated with 10 nm immunogold-conjugated secondary antibodies (Ted Pella, Redding, CA) in 1% NGS in TBST with 0.5% polyethylene glycol (20,000 MW) and stained with uranyl acetate and lead citrate. In this material, the axonal compartment was identified definitively by its synaptic contact. In the original experiments for Sun et al., 2011 (30), a random sample of micrographs were taken from the hippocampus CA1-stratum radiatum from two experiments with 3 + 3 and 2 + 2 WT + knockout (KO) mice; the two experiments produced similar results, and the data were then combined for a total of 646 WT and 642 KO spine profiles. Only the WT spine synapse profiles from the 2011 study were used for this study. The unpublished data from the analysis of the presynaptic terminals are presented here.

### Image analysis

#### Import and kymogram generation

Raw microscope time series were imported into ImageJ with StackReg and Bio-Formats plug-ins. A segmented line selection was drawn through the neurite of interest with the thickness adjusted to cover the diameter of the dendrite (7–12 pixels), with a resultant region of interest as shown in Fig. S1 *A*. Using the plugin KymoResliceWide, a kymogram was generated from the time series in which the horizontal dimension corresponded to the average pixel intensity along the diameter of the cell for each pixel distance from the soma and the vertical dimension was time. A sample segment of kymogram from a bleached neurite is shown in Fig. S1 *B*. The intermittent photobleaching of the region of interest is marked on the sample kymogram with leftward-facing blue arrows.

All kymograms were saved as TIF files, trajectories were saved as TXT files of coordinates, and all were imported into MATLAB (The MathWorks, Natick, MA) for further processing.

#### Differentiating neurites

Axons and dendrites were differentiated based on their morphology. Dendrites exhibit a steady decrease in diameter with distance from the soma and typically terminate within 1000 *μ*m. Axons extend for thousands of microns and have a relatively constant diameter. The most obvious changes in diameter are in neurite trunks; the axon initial segment is thin like the axons, at a few microns, whereas dendritic trunks can be several microns thick and broadly blend into the plasma membrane of the soma. In addition, dendrites branch more frequently and at more acute angles, whereas axons can branch at perpendicular or even obtuse angles. Oftentimes, the morphological features differentiating axons and dendrites are not visible in the frame of the time series, and additional global images of neuron must be referenced to distinguish neurites. An example of this is depicted in Fig. S1 *C*, in which the time series frame is outlined in red, but the defining morphological features of the axon and dendrite are only visible in the larger, global image. In Fig. S1 *C*, an axon (*red arrow*) and dendrite (*blue arrow*) exhibiting the aforementioned characteristics are labeled.

Beyond these morphological characteristics, the definitive way to differentiate neurites is with antibody staining for structural proteins exclusively found in one neurite type. Several coverslips were stained with ankyrin-G post-live imaging to confirm identification of the axon initial segment. One such neuron is depicted in Fig. S1 *D* and Video S2.

Video S1. A sample time series of Kv4.2-SGFP2 puncta trafficking

Video S2. Time series depicts characteristic high frequency trafficking in axons compared to dendrites

#### Contrast enhancement and thresholding

To improve the visibility of puncta trajectories, kymograms were enhanced using automated and manual methods in ImageJ. As an example, raw kymogram sections from a representative axon and dendrite are depicted in Fig. S2*, Ai and Bi.* ImageJ’s automatic optimization of brightness and contrast is first performed based on the image’s histogram (Fig. S2*, Aii and Bii*). Next, the brightness and contrast settings were manually adjusted by narrowing the visible display range (Fig. S2*, Aiii and Biii*). Lastly, a lower threshold was set, setting pixel values below this threshold to background, as shown in Fig. S2*, Aiv and Biv*.

#### Puncta trajectory selection

Puncta trajectories were traced using a segmented line selection. The brightness and contrast and threshold settings were adjusted and readjusted for regions of varying immobile fraction within the same kymogram. For instance, a dendrite that is bleached five times over the course of a recording, as in Fig. S1 *B*, required different contrast and threshold settings to visualize puncta in early beaches and later bleaches.

In some cases, puncta appear to merge into one trajectory or split into multiple trajectories. An example of this is depicted in Fig. S2 *C*. In these cases, when tracing trajectories, each parent and child path is designated as an individual trajectory, as in the three trajectories depicted in Fig. S2 *Cii*. The same protocol is followed for two puncta that seemingly merge into one trajectory.

Mobile puncta sometimes rapidly oscillate or vibrate in position. In these cases, if the specific path of the oscillations cannot be resolved, a trajectory was drawn through the mean position of the puncta. An example of this is depicted in Fig. S2 *D*, with a trajectory drawn through the mean position of an oscillation marked in Fig. S2 *Dii*.

Further, a punctum can increase and decrease in fluorescence or appear and disappear during a recording, as shown in Fig. S2 *E*. Because segmented line selections are never drawn through neurite branch points, this likely corresponds to Kv4.2-SGFP2 dispersion or accumulation. To minimize the subjectivity of trajectory selection through such events, each puncta trajectory was trimmed based on a threshold for net displacement, as described in the next section.

### Data analysis and modeling

#### Trajectory trimming

Because only mobile trajectories were considered, puncta with an immobile segment of trajectory before and/or after a mobile segment were trimmed. This was achieved by iterating through each trajectory and summing the net distance traveled. Portions of the trajectories up to the mobility threshold were removed, eliminating stall time before and after mobile segments. The minimal distance threshold was 5 *μ*m for both axon and dendrite trajectories. As an example, both trajectories shown in Fig. S2 *Eii and iii* are interpreted as the same trajectory (Fig. S2 *Eiv*) after trimming. This was useful in cases in which puncta appear or disappear on a kymogram, as in Fig. S2 *Ei*. This also relieves some degree of subjectivity surrounding puncta start and end points and in measurement of stall time.

#### Analysis of trajectory properties

Net displacement: The net displacement is the absolute distance along the neurite between the puncta’s final position and its initial position.
Average speed: Velocity was computed between each consecutive paired frames of a time series. The mean of the absolute values of these instantaneous velocities equals the average speed.
Stall time: Puncta stall time is defined as the fraction of total time during which puncta are traveling with a speed less than 0.1 *μ*m/s.
Mean-square displacement and superdiffusion: In addition to pure random walk motion, puncta may undergo long unidirectional runs. In an ensemble of trajectories, the bulk flow will then be characterized by diffusion with superlinear spread (superdiffusion). The degree of superdiffusivity in individual puncta trajectories was quantified as follows. Mean-square displacement (MSD) was computed by averaging the square of the difference between puncta coordinates some time separation *τ* apart. This was repeated for *τ* up to one-quarter the length of the recording duration. MSD was then plotted against *τ*, and resulting data were fitted to MSD(*τ*) = *Dτ*^*α*^ for each individual trajectory to obtain parameters *D* and *α. D* and *α* correspond to diffusion and superdiffusivity coefficients, respectively. In normal diffusion (a linear process), *α* = 1. *α* < 1 corresponds to subdiffusion, and *α* > 1 corresponds to superdiffusion. For the vast majority of trajectories, *α* > 1 (Fig. 3 *Biv*, *first column*). We therefore use the magnitude of *α* as a measure of the degree of superdiffusivity for individual puncta.

**Figure 1:**
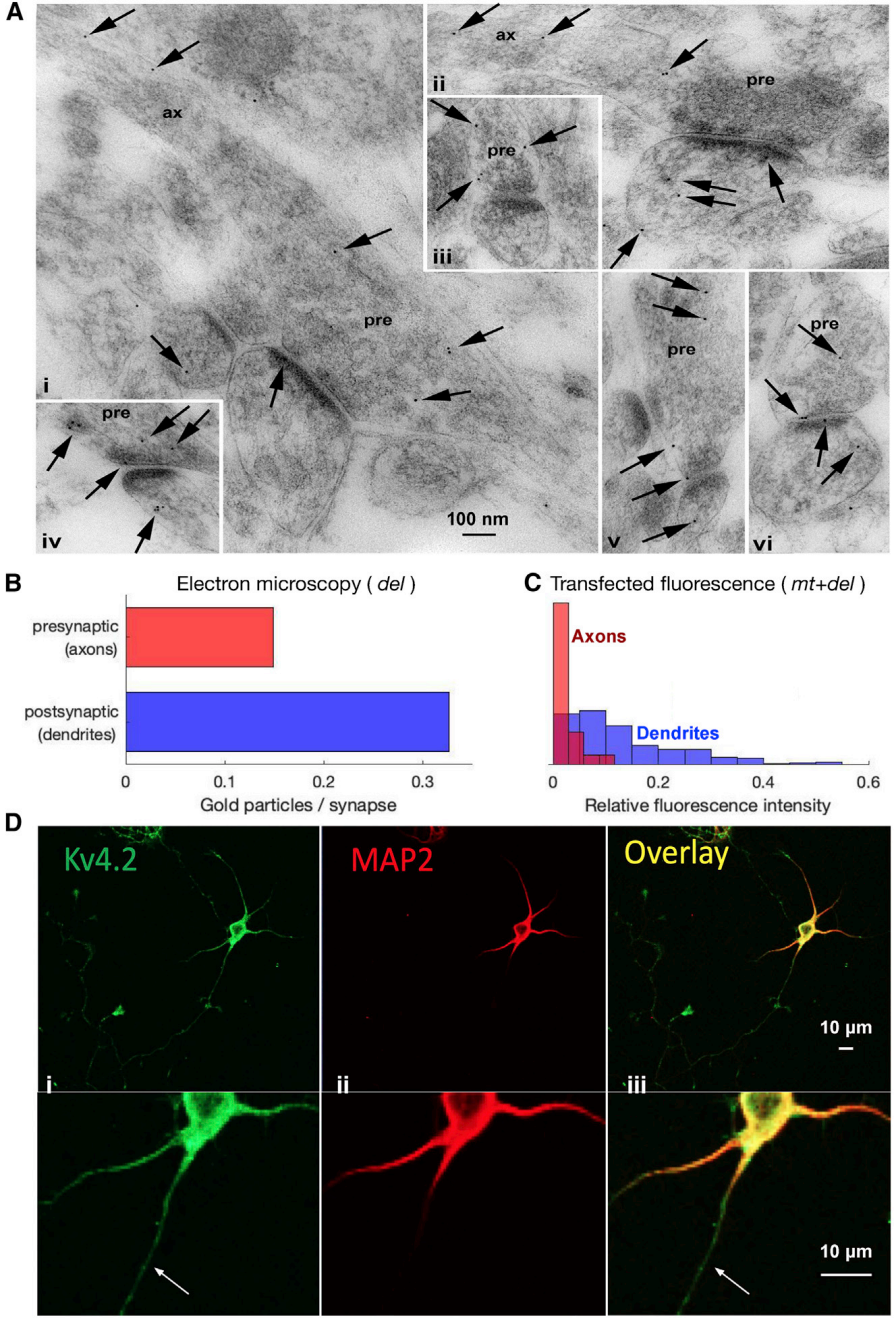
Kv4.2 preferentially localizes to dendrites in both endogenous and transfected expression systems, but axonal density is not negligible. (*A*) Immunogold localization (*arrows*) of Kv4.2 in the CA1 stratum radiatum of the hippocampus of WT mice. Synapse profiles show the presynaptic terminal (pre) contacting one or two postsynaptic spines. In (*i*) and (*ii*), the axon (ax) can be traced from the presynaptic terminal. Examples of gold labeling associated with the plasma membrane of the synapse and counted in the accompanying graph include those at the axon synaptic membrane shown in (*iv*), (*v*), and (*vi*); the axon extrasynaptic membrane shown in (*iii*) and (*v*); the dendrite synaptic membrane shown in (*i*) and (*vi*); and the dendrite extrasynaptic membrane shown in (*ii*). (*B*) Quantification of (*A*), in which presynaptic (axonal) and postsynaptic (dendritic) compartments yielded concentrations of 0.149 and 0.327 gold particles per synapse, respectively. Note that the bar “postsynaptic (dendrites)” was published previously in another form in Sun et al., 2011 (30) and is included here for comparison with axons. (*C*) Histogram of the relative prebleach fluorescence intensities of neurons transfected with Kv4.2-SGFP2, showing total subunit count. (*D*) E18 cultured rat hippocampal neurons at DIV5 were immunostained with Kv4.2 antibodies (*i*, *green*) to visualize the endogenous Kv4.2 and MAP2 antibodies (*ii*, *red*) to mark the dendritic arbor. The arrow indicates a representative axon with less Kv4.2 than the surrounding dendrites. To see this figure in color, go online.

**Figure 2:**
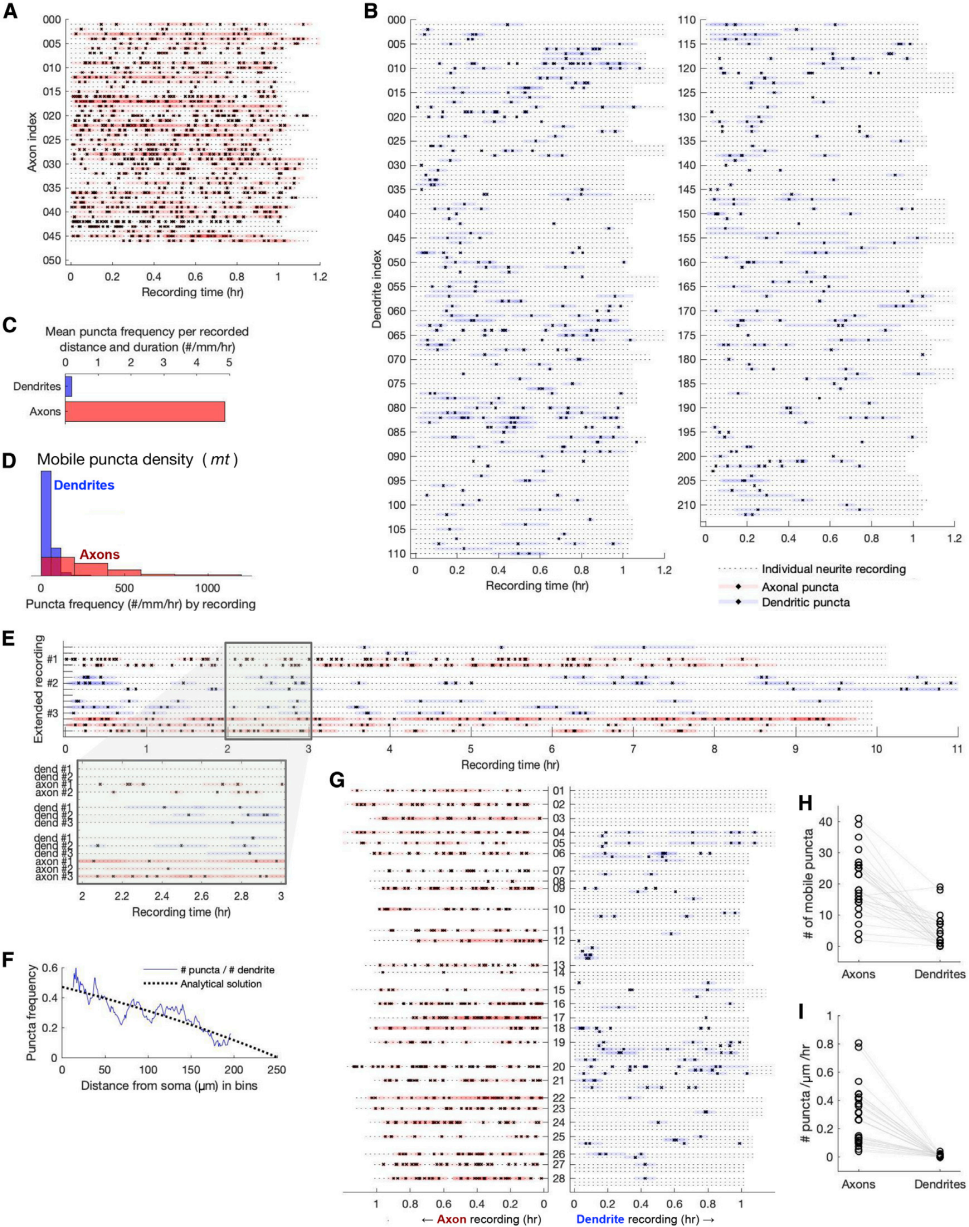
Kv4.2 microtubule-based trafficking is observed more frequently in axons than in dendrites. (*A*) Hour-long recordings of 46 axons are depicted, with highlighted sections indicating periods of puncta mobility. (*B*) Hour-long recordings of 213 dendrites are depicted, with highlighted sections indicating periods of puncta mobility. This subset of 478 dendrites has ≥1 mobile puncta. (*C*) Puncta frequency (*mt*) in axons and dendrites is standardized by total neurite length visualized and time recorded (units: number of puncta/mm/h). (*D*) Histogram depicting puncta frequency by neurite recording. (*E*) Three extended recordings that substantiate the puncta frequency discrepancy between axons and dendrites over extended periods of observation. (*F*) Puncta frequency (*mt*) decreases with distance from soma in dendrites, consistent with analytical solutions to the drift-diffusion equation. (*G*) Axons and dendrites originating from the same soma (same neuron) are depicted, demonstrating similar trends as those observed in isolated recordings. The central column of numbers indicates an arbitrary recording index for individual neurons. (*H*) Number of mobile puncta per neurite from concurrent recordings in (*G*). (*I*) Number of mobile puncta per neurite standardized by length and time for concurrent recordings from (*G*). To see this figure in color, go online.

**Figure 3:**
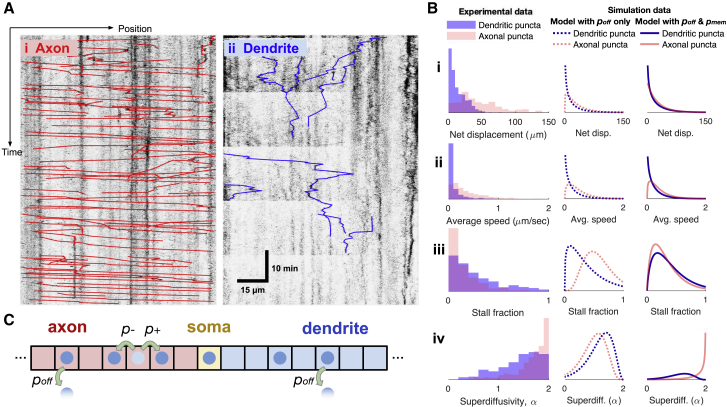
Kinetic differences between axons and dendrites are attributable to varying propensities for cargo offloading and unidirectional runs. (*A*) Representative histograms in axon (*i*) and dendrite (*ii*) show typical puncta trajectories and kinetics. Untraced histograms are depicted in Fig. S7. (*B*) Histograms for various transport parameters, normalized as probability density functions. On average, axonal puncta have greater net displacement (*i*), faster speed (*ii*), deceased stall time (*iii*), and increased unidirectional runs (*iv*). Model fits to these results using *p*_off_ alone (*second column*) as well as using *p*_off_ and *p*_mem_ (*third column*) are depicted. (*C*) Setup of stochastic simulations along linear multicompartment model (axon-soma-dendrite), with left and right jump and offloading rates depicted. Complete model is depicted in Fig. S9. To see this figure in color, go online.

Each trajectory fit to MSD vs. *τ* is depicted in Fig. S8 *A*, and the bold lines indicate median fits for each neurite type. Puncta in both dendrites and axons undergo motion with similar *D* (Fig. S8 *B*). However, the MSD tends to increase more rapidly with *τ* for axonal puncta than for dendritic puncta (Fig. 3 *Biv*). This corresponds to the axonal puncta taking more consecutive steps in the same direction, resulting in motion that is more directed than the memoryless walk of particles in typical diffusion. In other words, axonal puncta exhibited a higher degree of superdiffusivity than dendritic puncta. This discrepancy is consistent with inferred parameters $p_{\text{mem}}^{a}$ = 0.60 and $p_{\text{mem}}^{d}$ = 0.05 in the stochastic model.

#### Steady-state analysis for compartmental models

The cargo content of each compartment in a model is defined by a differential equation that sum the quantities of cargo entering and exiting that compartment. A generalized rate *v*_*d*,*r*_ from a donor *d* to receiver *r* transfers an amount of mass *dv*_*d*,*r*_. As an example, the system of differential equations for the simplest model in Fig. S6 *B* is as follows:

$$\begin{array}{l} {\dot{a}}_{\text{tot}}=+s_{d,a}d_{\text{tot}}-s_{a,d}a_{\text{tot}}\\ {\dot{d}}_{\text{tot}}=s_{a,d}a_{\text{tot}}-s_{d,a}d_{\text{tot}}. \end{array}$$

This system of equations can be solved at steady state to estimate the ratio of these rates. The steady-state assumption sets $\dot{a_{\text{tot}}}=\dot{d_{\text{tot}}}$ = 0. Then, rearranging either equation yields

$$\frac{d_{\text{tot}}}{a_{\text{tot}}}=\frac{s_{a,d}}{s_{d,a}}.$$

#### Bulk flow approximation using drift-diffusion-decay equation to estimate transport parameters

We used population dynamics for average puncta position as a function of distance along the dendrite. Fig. 2 *F* depicts the observed puncta frequency of dendritic puncta versus their measured distance from the soma. Puncta trajectories are grouped in 1-*μ*m bins along the dendrites and normalized by the number of dendritic recordings in each bin. To avoid numerical errors with low replicate count, we only considered bins with ≥30 dendrite recordings. The resulting distribution of puncta frequency is plotted (Fig. 2 *F*) and displays a trend of decreasing puncta frequency with distance from the soma.

This distribution of puncta frequency versus distance is expected for a collection of mobile particles obeying a drift-diffusion equation with low decay, which we demonstrate analytically. The one-dimensional drift-diffusion equation with decay is as follows:

(1) $$\frac{\partial m\left(x,t\right)}{\partial t}=D\frac{\partial^{2}m\left(x,t\right)}{\partial x^{2}}+v\frac{\partial m\left(x,t\right)}{\partial x}-\left(n_{\text{off}}+w_{\text{mt}}\right)m\left(x,t\right),$$

where *m*(*x*, *t*) denotes the concentration of some substance (heat, particles—in this case, Kv4.2-containing puncta) as a function of position *x* and time *t*. *D* is the diffusion coefficient, and *v* is the mean net velocity (drift). *n*_off_ is puncta offloading, and *w*_mt_ is degradation, together modeled as decay. Superdiffusion and unidirectional runs with *p*_mem_ are not explicitly estimated here but are incorporated into *D*, as established by Williams et al. (3). *p*_mem_ is fitted later based on puncta kinetics (Fig. 3).

Because all of our time series were in cells with strong fluorescence many hours after transfection, transport has reached a steady state in which an equal number of puncta enter and leave the recording region. Thus, $\frac{\partial m\left(x,t\right)}{\partial t}$ = 0, reducing Eq. 1 to

(2) $$D\frac{\partial^{2}m\left(x\right)}{\partial x^{2}}+v\frac{\partial m\left(x\right)}{\partial x}-\left(n_{\text{off}}+w_{\text{mt}}\right)m\left(x\right)=0.$$

This special case of the drift-diffusion equation is Poisson’s equation with decay, which we can solve as a boundary value problem (BVP) using the boundary conditions observed experimentally. From this steady-state distribution, we approximate whether puncta exhibit a forward (*p*_+_ > *p*_−_), backward (*p*_+_ < *p*_−_), or no (*p*_+_ ≈ *p*_−_) directional bias. The endpoints of our data,

(3) $$m\left(0\text{ μm}\right)=B_{P}\;\text{and}\;m\left(200\text{ μm}\right)=B_{D},$$

are set for fitting, where *B*_*P*_ and *B*_*D*_ are also the proximal and distal boundaries of the model. Our analytical result is the solution to Eq. 2 with boundary values as in Eq. 3, as follows:

(4) $$\begin{array}{l} m\left(x\right)=[B_{D}\text{exp}\left(\frac{1}{2}x\left(\frac{\sqrt{4D\left(n_{\text{off}}+w_{\text{mt}}\right)+v^{2}}}{D}-\frac{v}{D}\right)+\frac{100\sqrt{4D\left(n_{\text{off}}+w_{\text{mt}}\right)+v^{2}}}{D}+\frac{100v}{D}\right)-\\ B_{D}\text{exp}\left(\frac{1}{2}x\left(-\frac{\sqrt{4D\left(n_{\text{off}}+w_{\text{mt}}\right)+v^{2}}}{D}-\frac{v}{D}\right)+\frac{100\sqrt{4D\left(n_{\text{off}}+w_{\text{mt}}\right)+v^{2}}}{D}+\frac{100v}{D}\right)+\\ B_{P}\text{exp}\left(\frac{1}{2}x\left(-\frac{\sqrt{4D\left(n_{\text{off}}+w_{\text{mt}}\right)+v^{2}}}{D}-\frac{v}{D}\right)+\frac{200\sqrt{4D\left(n_{\text{off}}+w_{\text{mt}}\right)+v^{2}}}{D}\right)-\\ B_{P}\text{exp}\left(\frac{1}{2}x\left(\frac{\sqrt{4D\left(n_{\text{off}}+w_{\text{mt}}\right)+v^{2}}}{D}-\frac{v}{D}\right)\right)]/\left(\text{exp}\left(\frac{200\sqrt{4D\left(n_{\text{off}}+w_{\text{mt}}\right)+v^{2}}}{D}\right)-1\right). \end{array}$$

We fitted this analytical solution to the experimental data (Fig. 2 *F*) using least squares to obtain *B*_*P*_, *B*_*D*_, *D*, *v*, and (*n*_off_ + *w*_mt_):

(5) $$B_{P}=0.49;\;B_{D}=0.11;\;D=3.3\times 10^{-4};\;v=1.0\times 10^{-5};\;n_{\text{off}}+w_{\text{mt}}=5.4\times 10^{-8}.$$

This analytical solution is overlaid on the experimental data in Fig. 3 *B*.

*D* and *v* describe the bulk flow of a population of particles. When Eq. 1 is discretized, *D* and *v* characterize the rates of cargo transfer between adjacent compartments:

(6) $$D=\frac{f+b}{2}\;\text{and}\;v=b-f,$$

where *f* and *b* are the forward and backward rates of the discretized compartmental model. In the limit of large numbers, the propensities of a particle undergoing a random walk *p*_+_ and *p*_−_ are related to compartmental model rates *f* and *b* according to

(7) $$f=\frac{2p_{+}-{\left(p_{+}-p_{-}\right)}^{2}}{2}\;\text{and}\;b=\frac{2p_{-}-{\left(p_{+}-p_{-}\right)}^{2}}{2},$$

as derived in (3). We use the result from the BVP (Eq. 5) along with Eqs. 6 and 7. Because *v* ≈ 0 and *n*_off_ + *w*_mt_ ≈ 0, we estimate *p*_+_ = 0.500005 and *p*_−_ = 0.499995. Puncta have a minimal directional bias and *p*_+_ ≈ *p*_−_.

#### Stochastic model propensities p_*off*_ and p_*mem*_

In the stochastic variant of the model, we simulate individual puncta trajectories as unbiased bidirectional random walks on a one-dimensional lattice with additional propensities *p*_off_ and *p*_mem_. With each time step, individual puncta are removed from the lattice with propensity *p*_off_. The left and right jump propensities are therefore *p*_−_ = *p*_+_ = $\frac{1-p_{\text{off}}}{2}$ and *p*_+_ + *p*_−_ + *p*_off_ = 1.

*p*_mem_ is an additional memory feature, depicted in Fig. S9 *Aii and iii.* When *p*_mem_ = 0, the next directional step is independent of the previous step (Fig. S9 *Aii*). When *p*_mem_ = 1, the next step is always the same as the previous step (Fig. S8 *Aiii*). A linear interpolation between these extremes, as described in (3), produces a range of memory propensities that scales the average length of the unidirectional run.

#### Stochastic model fitting

To fit the stochastic model of a random walk modified with offload rate (*p*_off_) and memory (*p*_mem_) to experimental data, we use a combination of maximal likelihood estimation (MLE) and least-squares fitting. Experiment and model data are normalized and fit to a *γ* distribution using MLE (MATLAB function fitdist). A *γ* distribution accommodates all data given its continuity and coverage of a semi-infinite [0, ∞) interval.

The shape and scale parameters of *γ* fits are compared using nonlinear least-squares data fitting (MATLAB function lsqcurvefit). Generating stochastic model estimates requires a large number of simulated puncta *N*_*s*_ to produce consistent distributions. We employ a moderate *N*_*s*_ = 10,000 and increase the finite difference step size of lsqcurvefit. A script continuously iterates between 1) running *N*_*s*_ iterations of the stochastic model, 2) MLE of stochastic data, and 3) least-squares fitting of distribution parameters to match those of experimental data. A full description of this method is presented in the Supporting materials and methods.

## Results

We first characterized the intracellular distribution of Kv4.2 in our preparation to establish consistency with previously reported results and to validate that the expression of the fluorescent-tagged construct we used for live imaging did not substantially deviate from endogenous expression patterns. Throughout our results, we will refer to two subpopulations of protein subunits: the fraction undergoing active transport on microtubules, denoted *mt* (for “microtubule”), and the remaining fraction that is not undergoing active transport, which we denote *del* (“delivered”). It is important to note that the delivered fraction, *del*, comprises the cytosolic pool as well as the functional, membrane-bound fraction. Imaging modalities differ in their ability to distinguish and reliably quantify the *mt* and *del* populations. We therefore use three different comparative measurements: electron microscopy (EM), fluorescent immunostaining, and live imaging.

To quantify endogenous expression, we first used EM after immunogold labeling of endogenous Kv4.2 subunits. Owing to the inherent constraints of EM imaging, we quantified axonal and dendritic expression in identifiable pre- or postsynapses and contiguous extrasynaptic regions, which correspond to *del* cargo densities. We imaged 624 presynaptic and 646 postsynaptic regions. Example micrographs in Fig. 1 *A* show an axon (ax) that can be traced to presynapses (pre).

Sampled immunogold particles identified in the synapses and perisynapses are broadly divided into pre- and postsynaptic regions corresponding to axons and dendrites, respectively. Axons contained 30.6% of all gold particles and 0.15 particles/synapse. Dendrites contained 69.4% of particles and 0.33 particles/synapse (Fig. 1 *B*). This is consistent with previous localization studies (17) in showing substantial, non-negligible subunit localization in axons. Gold particles are also subdivided into synaptic and extrasynaptic regions. In both axons and dendrites, under one-third of particles (28.0 and 32.2%, respectively) of particles were found in synaptic spaces, with the remaining two-thirds in extrasynaptic regions. These percentages and gold particle frequencies are summarized in Table S1.

We next confirmed preferential Kv4.2 expression in dendrites using fluorescence labeling in both endogenous and transfected expression systems. Fig. 1 *D* depicts a neuron with multiple dendrites and one axon stained for somatodendritic marker MAP2 (Fig. 1 *Dii*, *red*). We found substantial Kv4.2 (Fig. 1 *Di*, *green*) in both dendrites and the axon (marked with the *arrow*). The axon exhibits lower fluorescence intensity than the dendrite but is well above levels of background staining. Measurements of Kv4.2-SGFP2 transfected neurons also corroborate this trend, depicted as a histogram of prebleach fluorescence intensity (Fig. 1 *C*). Dendrites contain significantly more Kv4.2 per unit area compared to axons. Taken together, these results establish that the static fraction of Kv4.2 subunits preferentially localizes in dendrites, but its expression in axons is non-negligible, consistent with previous studies (16,17,20).

### Kv4.2 microtubule-based trafficking is observed more frequently in axons than in dendrites

**Figure 4:**
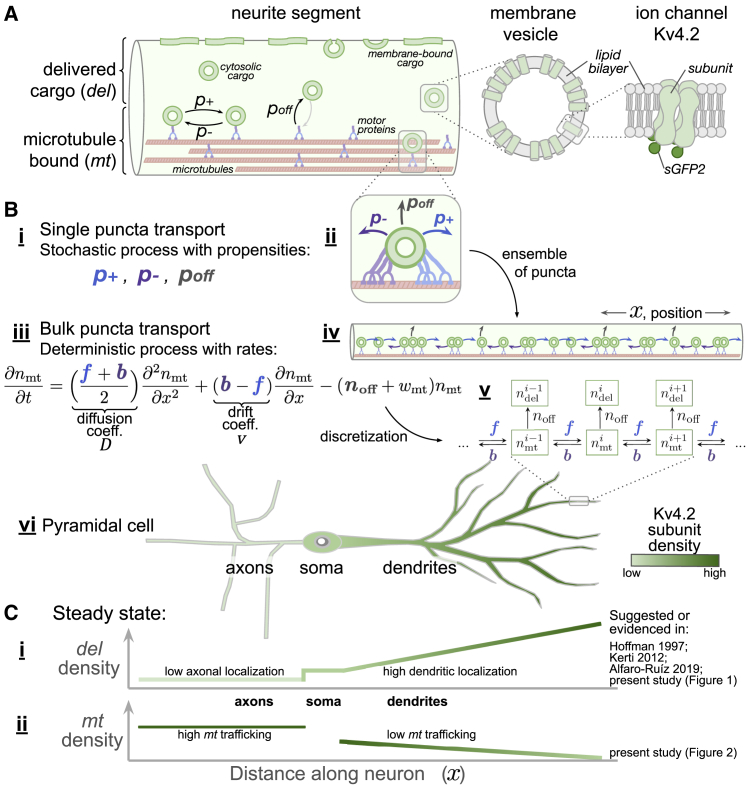
Mathematical model of intracellular transport. (*A*) Kv4.2 subunits are divided into microtubule-bound (*mt*) and delivered (*del*) cargo populations. (*B*) Individual vesicles containing cargo (puncta) have microscopic dynamics modeled as a directed random walk (*i* and *ii*). At the population level, the density of cargo (*iv*) behaves as a deterministic process, described by a drift-diffusion equation with decay (*iii*), which can be discretized into a compartmental model (*v*). Compartmental models can represent neurites of a full neuron morphology (*vi*). (*C*) Steady-state cargo densities depend on the relative rates of delivery and transport. Delivered (*del*) Kv4.2 density is low in axons and high in dendrites and increases with dendritic distance (*i*). Microtubule-bound (*mt*) trafficking densities are high in axons and low in dendrites (*ii*). To see this figure in color, go online.

We next measured the frequency, density, and kinetic properties of actively transported Kv4.2 subunits on microtubules (the *mt* population) in both axons and dendrites. To establish reliable estimates, we performed 129 hour-long recordings in neurites of cultured rat hippocampal cells. In total, 507 mobile Kv4.2-SGFP2 puncta were identified among 478 recorded dendrites, and 961 mobile puncta were identified in 46 axons (see Materials and methods). We define mobile puncta as distinct points of fluorescence observed in motion during a recorded time series (Video S1). Ion channels and other integral membrane proteins are transported in membrane vesicles on microtubules (34,35), as depicted in Fig. 4, *Ai*–*iii*. To measure puncta mobility, we created kymograms from the time series recordings (Fig. S1*, A and B*), traced puncta paths, and filtered for mobile trajectories (see Materials and methods). We assume that axonal and dendritic puncta contain a similar number of Kv4.2 subunits such that our recordings provide a comparative measure of *mt* density between neurite types.

To validate that mobile puncta are transported via active, motor protein-based transport, we applied the microtubule-disrupting drug colchicine (36, 37, 38). On average, colchicine administration resulted in a substantial (>60%) decrease in the number of mobile puncta when compared with vehicle (Fig. S5). The Kv4.2-SGFP2 puncta transport that we observe is thus likely to be an active, microtubule-dependent process.

The durations over which puncta are mobile are depicted in Fig. 2, *A* and *B* for axons and dendrites, receptively. Of the 478 dendrites in hour-long recordings, only 213 dendrites (45%) exhibited at least one mobile punctum and are presented in Fig. 2 *B*. Mobile puncta appeared consistently in axons, whereas in dendrites, mobile puncta appear intermittently or not at all. The average length of a sampled region was 85.4 *μ*m in axons compared to 52.3 *μ*m in dendrites. When standardizing these measurements for recording duration and neurite length, the difference in puncta frequency (*mt*) is 4.9 puncta/mm/h in axons vs. 0.18 puncta/mm/h in dendrites, depicted in Fig. 2 *C*. Puncta frequency (*mt*) in dendrites drops to 0.039 puncta/mm/h when considering dendritic recordings with zero mobile puncta (not depicted in Fig. 2 *B*). A histogram showing puncta frequency (*mt*) by neurite recording is depicted in Fig. 2 *D*.

To control for the possibility of global trafficking failure in dendrites that did not show puncta during hour-long recordings, we performed extended recordings lasting 10 h, shown in Fig. 2 *E*. The trend in frequency for extended recordings is consistent with that of hour-long recordings, suggesting that hour-long recordings with no puncta are simply a result of sampling.

Puncta frequency (*mt*) was found to be strongly, negatively correlated with distance from soma in dendrites (Fig. 2 *F*). We found no strong correlation between transit frequency and degree of branching from primary (apical) dendrites to quaternary branches (Fig. S3). To ensure that puncta visibility is not an artifact of the fluorescence intensity, we plot puncta frequency (*mt*) versus standardized neurite intensity and find no strong correlation (Fig. S4).

In some cases, it was possible to reliably identify and record from axons and dendrites originating from the same soma to control for cell-to-cell variation in trafficking or metabolism rates. Axons and dendrites from these 28 recordings are depicted alongside each other in Fig. 2 *G*. In all but one case, axons possessed the majority of mobile puncta, even though multiple dendrites were recorded for most neurons. Comparisons of raw puncta count and standardized puncta count (number of puncta/*μ*m/h) are depicted in Fig. 2, *H* and *I*. After standardizing measurements to sampling distance and duration, the axons average a 36-fold increase over the simultaneously recorded dendrites from the same cell.

Taken together, these data establish that actively transported Kv4.2 puncta are present in significantly higher frequencies and densities in axons as compared to dendrites. Thus, the density of trafficked (*mt*) cargo follows the opposite trend of delivered (*del*) subunit density in axons and dendrites.

### Kinetics of cargo motion in axons and dendrites reflects differential demand and trafficking mechanisms

The observed disparity between actively transported cargo versus delivered cargo in axons and dendrites raised the question of whether there were differences in the kinetic properties of puncta motion in these compartments. We analyzed Kv4.2 puncta trajectories in axons and dendrites by recording time-lapse images of neurite segments and tracing puncta trajectories on the resulting kymograms (see Materials and methods).

Representative kymograms from axons and dendrites are shown in Fig. 3, *Ai* and *ii*, respectively. Population measurements of puncta kinetics are shown in Fig. 3 *B* (*first column*). On average, axonal puncta have greater net displacement (Fig. 3 *Bi*), faster speed (Fig. 3 *Bii*), deceased stall time (Fig. 3 *Biii*), and increased superdiffusivity (Fig. 3 *Biv*). The computation of these four kinetic measures is detailed in the Materials and methods. Quantifying superdiffusivity involves fitting the MSD of each trajectory to a curve of anomalous diffusion, as discussed in the Materials and methods and depicted in Fig. S8. Taken together, axonal puncta undergo unidirectional runs at high speeds, whereas dendritic puncta appear to change direction more frequently and stall longer. This observation is consistent with low functional expression and a low delivered density of cargo in axons.

We next asked whether a microscopic model of transport could account for observed differences in axonal and dendritic transport and whether these differences might (in part) be explained by differences in cargo demand and sequestration. We expected that higher sequestration rates to the delivered cargo pool (*del*) in dendrites would lead to more interruptions in the directed movement of transported (*mt*) particles, with the opposite trend in axons. We formulated a simple mathematical model of the dynamics of discrete cargo particles as a (directed) random walk (11,39,40).

A cartoon of a neurite segment in Fig. 4 *A* depicts cargo-containing membrane vesicles, corresponding to observed Kv4.2-SGFP2 puncta, undergoing active transport and delivery to a local pool. In keeping with our previous conventions, we assume that cargo belongs either to the microtubule-bound *mt* fraction or the delivered *del* fraction. A microtubule-bound vesicle is attached to opposing motor proteins (Fig. 4 *Bii*), which subject it to stochastic anterograde and retrograde forces (11, 12, 13, 14,41, 42, 43, 44). We use a stochastic model to represent the net effects of collective forces on individual puncta. In the simplest variant of our model, puncta move in a modified random walk: right (*x* = *i* → *i* + 1) with propensity *p*_+_ and left (*x* = *i* → *i* − 1) with propensity *p*_−_ per time step Δ*t*. Puncta also detach from the microtubule with net propensity *p*_off_ per time step Δ*t*.

Puncta trajectories were simulated on a one-dimensional lattice of spatial bins. A schematic of the model is shown in Fig. 3 *A* and contains three types of compartment: axon (A), soma (S), and dendrite (D). Each punctum begins in the S compartment. Puncta in axons and dendrites have distinct offload propensities $p_{\text{off}}^{a}$ and $p_{\text{off}}^{d}$, consistent with differing cargo demands in each neurite type. We inferred parameters of this model from our experimental measurements of puncta trajectories using maximal likelihood (see Materials and methods and Supporting materials and methods).

The result of fitting for *p*_off_ is in Fig. 3 *B* (*second column*). Optimal parameter estimates for surface delivery gave $p_{\text{off}}^{a}<p_{\text{off}}^{d}$ ($p_{\text{off}}^{a}$ = 0.01 and $p_{\text{off}}^{d}$ = 0.04), consistent with our own observations and published evidence for stronger Kv4.2 sequestration in dendrites versus axons (8,15, 16, 17,20,23,45). Thus, a memoryless random walk with differential *p*_off_ in axons and dendrites is sufficient to explain the differences in net displacement and average speed of cargo (Fig. 3, *Bi* and *ii*). However, stall time distributions and superdiffusivity are not captured fully by this model (Fig. 3, *Biii* and *iv*).

We next incorporated an additional state into the stochastic model with parameter *p*_mem_ that introduces memory into the kinetics (depicted in Fig. S9 *A* and further explained in Materials and methods). *p*_mem_ is the probability that a punctum repeats its previous step, giving rise to extended runs if *p*_mem_ > 0. The result of fitting the model with memory (0 < *p*_mem_ < 1) is shown in Fig. 3 *B* (*third column*). We again found $p_{\text{off}}^{a}<p_{\text{off}}^{d}$, producing the same trends in displacement and speed (Fig. 3, *Bi* and *ii*). Optimal estimates of the memory term were $p_{\text{mem}}^{a}$ = 0.60 and $p_{\text{mem}}^{d}$ = 0.05. This is consistent with high superdiffusivity in axons and elevated stall times in dendrites (Fig. 3, *Biii* and *iv*; Fig. S9 *B*), as observed in trajectories (Fig. S6*, A and B*).

Together, this analysis suggests mechanistic differences in the transport of Kv4.2 in axons and dendrites. Increased net displacement, average speed, and puncta frequency in axons are explained by a random walk with minimal delivery (*p*_off_) in axons, consistent with Kv4.2 localization to dendrites. However, $p_{\text{off}}^{a}<p_{\text{off}}^{d}$ only partially explains the longer observed runs. Other kinetic parameters—stall time and superdiffusivity—require an additional memory term *p*_mem_ in our model, suggesting a distinct axonal transport mechanism.

### A mathematical model of bulk intracellular transport can account for trafficked and delivered cargo densities

We next asked whether a model of intracellular transport based on the sushi belt model could account for the bulk distributions of actively transported and delivered Kv4.2 cargo. Fig. 4 outlines how this model is derived and how bulk transport relates to the motion of individually measured puncta and delivered cargo (Fig. 4 *A*). The previous results established that the microscopic dynamics of trafficked cargo conform to a random walk. A standard derivation (Fig. 4, *Bi*–*iv*; Supporting materials and methods) shows that the density of a large population of cargo undergoing such stochastic motion is described by a drift-diffusion equation (3,46):

(8) $$\frac{\partial n_{\text{mt}}}{\partial t}=\left(\frac{f+b}{2}\right)\frac{\partial^{2}n_{\text{mt}}}{\partial x^{2}}+\left(b-f\right)\frac{\partial n_{\text{mt}}}{\partial x}-\left(n_{\text{off}}+w_{\text{mt}}\right)n_{\text{mt}}.$$

This partial differential equation relates the position *x* and time *t* dependence of microtubule-bound puncta density *n*_mt_ with rates for bulk flow directed to the right *f*, left *b*, and off the microtubule *n*_off_. These flow rates relate to diffusion *D* and drift *v* coefficients and to stochastic propensities *p*_+_ and *p*_−_ (as described in Materials and methods; Eqs. 6 and 7). Microtubule-bound puncta degrade with rate *w*_mt_.

For the purpose of simulation and for fitting to data, Eq. 8 can be discretized into a compartmental model in which the flow of cargo obeys the law of mass action (Fig. 4 *Bv*). The spatial scale of compartmentalization can be refined or lumped into smaller or larger compartments, respectively, to account for spatially averaged average or distance-dependent measurements (3,47). We constrained compartmental models of bulk transport to our experimental measurements to test whether global microtubule-based trafficking, combined with differential local sequestration, can account for broad relationships observed in our data and in previous studies. Specifically, we asked the following:

1) Is the disparity between actively transported cargo and delivered cargo in axons and dendrites predicted by the sushi belt model?
2) Can such a model reconcile our trafficking measurements with the known functional dendritic density profile of Kv4.2 reported elsewhere (16,17)?

These questions are schematized in Fig. 4, *Ci* and *ii*, which show the contrasting observed densities of the *del* and *mt* populations, respectively, throughout a neuron.

**Figure 5:**
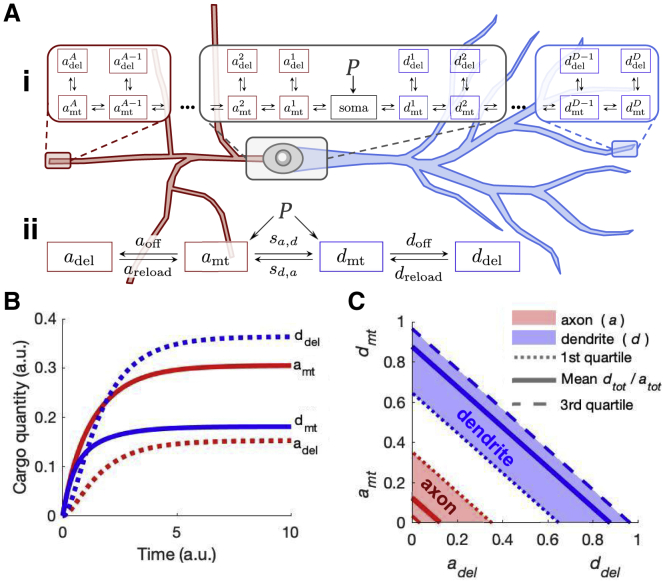
Disparity between delivered Kv4.2 (*del*) subunit density and puncta frequency (*mt*) in lumped neurites is explained by a mass-action model. (*A*) Box diagram of mass-action model of axon and dendrite transport. In a full morphology (*i*), the central soma is surrounded by microtubule (*mt*) and delivered (*del*) cargo compartments for axons *a* and dendrites *d*. Arrows denote rates of cargo transfer between compartments. A lumped variant (*ii*) can accommodate experimental constraints to simulate disparities in subunit density between axons and dendrites. (*B*) Result of simulation with experimentally constrained rates, corroborating *mt* and *del* densities observed experimentally. (*C*) Analytical result demonstrating negative correlation between *a*_del_ and *a*_mt_ or *d*_del_ and *d*_mt_ when restricted to a constant total (*tot*) density. To see this figure in color, go online.

We first asked whether a lumped compartmental model, constrained by our experimental measurements, can account for measured relationships between trafficked and delivered cargo. Fig. 5 *Ai* depicts a full neuron morphology discretized into spatial compartments. In each compartment, we assumed that cargo is either undergoing transport on microtubules (subscript *mt*) or delivered (subscript *del*) in axonal (*a*) and dendritic (*d*) compartments. As depicted in Fig. 4 *Ai*, *mt* denotes microtubule-bound cargo. Compartments *del* account for all channel subunits that have detached from microtubules, including those in local pools, in the cytosol, and on the plasma membrane. Rates from *mt* to *del* represent cargo offloading from the microtubules (*a*_off_, *d*_off_). The reverse rates (*a*_reload_, *d*_reload_) represent cargo reloading from *del* to *mt*.

Measurements in our study (Figs. 1 and 2) and others (17,20) do not provide axonal data as a function of axonal distance. To incorporate axonal data into a model, we coarsened into a lumped compartmental model that considers only the average density of material in axons and dendrites, irrespective of location (Fig. 5 *Aii*). In the lumped model, *s*_*a*,*d*_ and *s*_*d*,*a*_ represent the net flux of cargo passing between axons and dendrites on microtubules. Allowing separate fluxes, *s*_*a*,*d*_ and *s*_*d*,*a*_, provides for asymmetric flow due to sorting mechanisms that are known to regulate cargo entry into both axons and dendrites (48, 49, 50), including mechanisms specific for Kv4.2 (51). All other rates and compartments are as previously described.

The system of differential equations for the lumped compartmental model (Fig. 5 *Aii*) is as follows:

(9) $$\begin{array}{l} {\dot{a}}_{\text{del}}=a_{\text{off}}a_{\text{mt}}-a_{\text{reload}}a_{\text{del}}\;-w_{\text{del}}a_{\text{del}}\\ {\dot{a}}_{\text{mt}}=P+a_{\text{reload}}a_{\text{del}}-a_{\text{off}}a_{\text{mt}}+s_{d,a}d_{\text{mt}}-s_{a,d}a_{\text{mt}}\;-w_{\text{mt}}a_{\text{mt}}\\ {\dot{d}}_{\text{mt}}=P+d_{\text{reload}}d_{\text{del}}-d_{\text{off}}d_{\text{mt}}+s_{a,d}a_{\text{mt}}-s_{d,a}d_{\text{mt}}\;-w_{\text{mt}}d_{\text{mt}}\\ {\dot{d}}_{\text{del}}=d_{\text{off}}d_{\text{mt}}-d_{\text{reload}}d_{\text{del}}\;-w_{\text{del}}d_{\text{del}}, \end{array}$$

where a generalized rate *v*_*d*,*r*_ describes the flow of mass *dv*_*d*,*r*_ from donor *d* to receiver *r* compartments (detailed in Materials and methods). The lumped model does not contain a soma compartment. To account for biosynthesis, we add a fixed production term *P* to both dendritic and axonal microtubule compartments. Note that flux into both is not assumed to be equal because the flow between axons and dendrites is accounted for by parameters *s*_*d*,*a*_ and *s*_*a*,*d*_. *w* represents cargo degradation (not depicted in Fig. 5 *Aii*), which, consistent with endolysosomal and authophagic degradation pathways of membrane proteins (52), is faster in *del* than *mt*: *w*_del_ > *w*_mt_. The remaining rates in Eq. 9 are estimated from experimental results as described here.

We set *s*_*a*,*d*_ and *s*_*d*,*a*_ to a timescale slower than the other four rates because the distances traveled on microtubules are substantially longer than from *mt* to *del*, especially for large neuron morphologies. To enable a (quasi) steady-state estimate, *s*_*a*,*d*_ and *s*_*d*,*a*_ are set to a timescale 10-fold slower than the other rates, although more modest timescale separation produced the same qualitative result.

We next constrained the rates in this model with our experimental measurements. Rates *s*_*a*,*d*_ and *s*_*d*,*a*_ are estimated using the total (*del* + *mt*) subunit density in axons *a*_tot_ and dendrites *d*_tot_. We grouped *mt* and *del* compartments (from Fig. 5 *Aii*) to produce a model with only *a*_tot_ and *d*_tot_, depicted in Fig. S6 *B*, where

(10) $$a_{\text{tot}}=a_{\text{mt}}+a_{\text{del}}\;\text{and}\;d_{\text{tot}}=d_{\text{mt}}+d_{\text{del}}.$$

Fluorescence microscopy of Kv4.2 captures *a*_tot_ and *d*_tot_, in which we found a *d*_tot_/*a*_tot_ ratio of 7.1:1 (see Fig. 1 *C*). Predominant dendritic segregation of the channel is corroborated by other localization studies (8,15,17,20). Steady-state analysis of the differential equations (see Materials and methods) for this model variant (Fig. S6 *B*) yields

(11) $$\frac{d_{\text{tot}}}{a_{\text{tot}}}=\frac{s_{a,d}}{s_{d,a}}\approx \frac{7.11}{1}.$$

Rates *s*_*a*,*d*_ and *s*_*d*,*a*_ are normalized to axonal measures.

Constraining offload (*a*_off_, *d*_off_) and reload (*a*_reload_, *d*_reload_) rates requires estimates of *mt* and *del* cargo in both axons and dendrites. We estimate steady-state *mt* compartments (*a*_mt_, *d*_mt_) using experimental data for puncta frequency (Fig. 2 *C*). Normalizing to the axon, *d*_mt_ = 0.04 and *a*_mt_ = 1. We estimate steady-state *del* compartments (*a*_del_, *d*_del_) using our data from EM in synapses (see Fig. 1 *B*; Table S1). Normalizing to the axon, *d*_del_ = 2.24 and *a*_del_ = 1.

To estimate offload and reload rates from *mt* and *del* densities, we modeled axons and dendrites individually, as depicted in Fig. S6 *C*. As twice before, we arrived at expressions that allowed us to solve for ratios of rates:

(12) $$\frac{a_{\text{mt}}}{a_{\text{del}}}=\frac{a_{\text{reload}}}{a_{\text{off}}}\approx \frac{1}{1}\;\text{and}\;\frac{d_{\text{mt}}}{d_{\text{del}}}=\frac{d_{\text{reload}}}{d_{\text{off}}}\approx \frac{0.0374}{2.24}.$$

Together, these estimates (Eqs. 11 and 12) provide constraints for all rates in the lumped model variant (Fig. 5 *Aii*).

The behavior of this model is shown in Fig. 5 *B*. At steady state, the negative correlation between *mt* and *del* compartments in both neurites is clear: *a*_del_ < *a*_mt_ and *d*_mt_ < *d*_del_. In the context of mass action, the result is intuitive. Because cargo demand in axons is restricted (*a*_del_ < *d*_del_), more cargo tends to accumulate in the microtubules of axons versus those of dendrites (*a*_mt_ > *d*_mt_).

We next analyzed the negative correlation between *mt* and *del* compartments using Eqs. 10 and 11. We normalized Eq. 11 to a total mass *a*_tot_ + *d*_tot_ = 1 (*a*_tot_ = 0.12, *d*_tot_ = 0.88) such that each density (*a*_mt_, *a*_del_, *d*_mt_, *d*_del_) is a fractional quantity. The resulting steady-state densities of *del* and *mt* cargo are plotted in Fig. 5 *C*. Shaded regions indicate the range of *a*_tot_ and *d*_tot_ from first to third quartiles on Fig. 1 *C*. Quantities of cargo *mt* have a clear negative correlation with *del*, and this result holds for any *a*_tot_ and *d*_tot_.

### Measured active transport dynamics can account for functional Kv4.2 density along the somatodendritic axis

We next asked whether the transport model described previously could account for the spatial profiles of dendritic distributions of transported and delivered cargo. We measured a decreasing *mt* profile along dendrites with distance from the soma (Fig. 2 *F*). On the other hand, functional and localization studies show that Kv4.2 current and subunit density increase along this axis (16,17).

**Figure 6:**
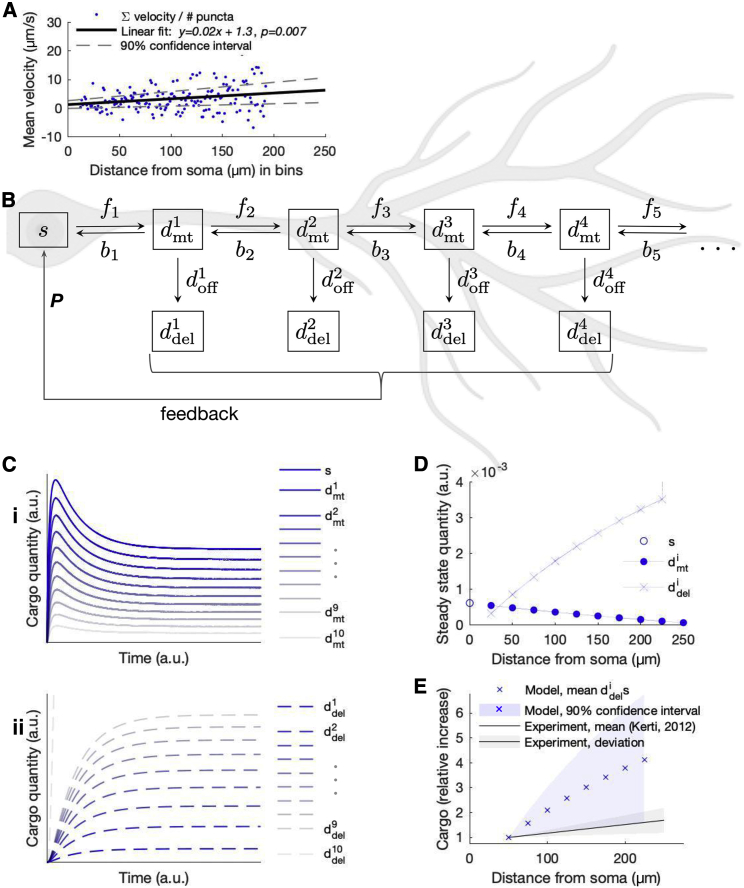
Opposing gradients in *del* and *mt* along somatodendritic axis are reconciled with mass-action kinetics. (*A*) The mean instantaneous velocities for all dendritic puncta are standardized by puncta frequency along the length of the dendrite. A linear tread line is plotted through the data with 90% confidence intervals, indicating a positive (distal) velocity bias that increases with distance from soma. (*B*) Box diagram of a mass-action model of dendritic transport and delivery with feedback. The dendrite is spatially discretized, with each discretization *i* comprising a microtubule-bound $d_{\text{mt}}^{i}$ and delivered $d_{\text{del}}^{i}$ compartment. *f*_*i*_-values, *b*_*i*_-values, and $d_{\text{off}}^{i}$-values denote rates between compartments. Degradation rates for all compartments are simulated but not depicted. (*C*) Simulation results for $d_{\text{mt}}^{i}$ (*i*) and $d_{\text{del}}^{i}$ (*ii*). (*D*) Steady-state concentrations of all compartments. (*E*) Steady-state concentrations of $d_{\text{del}}^{i}$-values standardized by $d_{\text{del}}^{2}$ at 50 *μ*m overlaid on equivalently standardized experimental data of Kv4.2 localization (17). To see this figure in color, go online.

To examine whether these observations were consistent with the model, we spatial discretized the model (Fig. 5 *Ai*). Somatic and dendritic compartments are depicted in Fig. 6 *B*, where $d_{\text{mt}}^{i}$ and $d_{\text{del}}^{i}$ represent microtubule-bound and delivered cargo, respectively. We considered a linear dendritic branch extending 250 *μ*m from the soma. To constrain the steady-state concentrations of *mt* compartments (*s*, $d_{\text{mt}}^{1},d_{\text{mt}}^{2},\ldots ,d_{\text{mt}}^{10}$), we used experimental values obtained in Fig. 2 *F*. *f*_*i*_ and *b*_*i*_ denote the forward and backward transport rates along the microtubule. We have combined microtubule offload and reload rates into a net rate $d_{\text{off}}^{i}$ for each $d_{\text{mt}}^{i}$.

We next computed directional bias in punctal velocity as a function of distance to constrain rates *f*_*i*_ and *b*_*i*_. We averaged the instantaneous velocities of each puncta trajectory in bins by distance from the soma. Mean puncta velocity showed an increasing linear trend with *p*-value < 0.01, as plotted in Fig. 6 *A* with 90% confidence intervals. With a positive *y* intercept and slope, the mean punctal velocity is directed distally and increases with distance from the soma. That is, *f*_*i*_ > *b*_*i*_ and *f*_*i* + 1_ ≫ *b*_*i* + 1_. The velocities in Fig. 6 *A* range from 1.5 to 5.2 *μ*m/s and are scaled according to the spatial discretization of the model to estimate *f*_*i*_-values and *b*_*i*_-values. The diffusion coefficient *D* = $\frac{f_{i}+b_{i}}{2}$ was estimated using Eq. 6, and *D* remains constant throughout the dendritic tree.

We constrained the cargo offloading rate $d_{\text{off}}^{i}$ in each spatial compartment by solving the corresponding equations at steady state (Fig. 6 *B*). We found that a profile of increasing *f*_*i*_-values and decreasing *b*_*i*_-values with distance from the soma produces an increasing profile of $d_{\text{off}}^{i}$-values. In other words, for cargo with an increasing directional bias such that 0 < *f*_*i*_ − *b*_*i*_ < *f*_*i* + 1_ − *b*_*i* + 1_ and decreasing *mt* profile, mass action dictates increasing offload rates $d_{\text{off}}^{i}<d_{\text{off}}^{i+1}$ with distance from the soma.

Increasing $d_{\text{off}}^{i}$-values can produce *del* profiles that have the opposite spatial profile as *mt* densities. To demonstrate this, we simulate regulated Kv4.2 production, distribution, and delivery in our model. In the soma, Kv4.2 biosynthesis *P* is regulated by active subunits in *del* compartments, as depicted in Fig. 6 *B*. The equation for negative feedback is

$$P=K_{P}\left(d_{\text{avg},\text{del}}^{\text{target}}-\frac{\sum_{i=1}^{10}d_{\text{del}}^{i}}{10}\right),$$

where $d_{\text{avg},\text{del}}^{\text{target}}$ is the target *del* concentration (setpoint), $\frac{\sum_{i=1}^{10}d_{\text{del}}^{i}}{10}$ is mean delivered cargo (process variable), and *K*_*P*_ is the proportional gain. This control loop feedback mechanism is consistent with experimental observations that Kv4.2 expression is regulated as a function of neuron excitability (22, 23, 24,53). However, the exact nature of the feedback signal is unknown. We therefore used the averaged delivery rate over all $d_{\text{del}}^{i}$, which amounts to simple proteostasis and is a realistic feedback signal in a neuron (54).

The result of simulating this model is depicted in Fig. 6 *C*. $d_{\text{mt}}^{i}$-values assume a profile similar to that observed experimentally (Fig. 2 *F*), with decreasing density with dendritic distance (Fig. 6 *Ci*). $d_{\text{del}}^{i}$-values form the opposite profile—increasing density with dendritic distance (Fig. 6 *Cii*). Steady-state densities versus position along the dendrite are plotted in Fig. 6 *D*.

The increasing $d_{\text{del}}^{i}$ density is notable because localization experiments (17) and, to a larger degree, recordings of A-type current (16) both demonstrate increasing profiles with distance from the soma. In this analysis, the gradient of steady-state $d_{\text{del}}^{i}$-values (Fig. 6 *D*) largely depends on that of the mean velocities (Fig. 6 *A*) used to constrain the directional bias *f*_*i*_ > *b*_*i*_. In Fig. 6 *E*, we plot $d_{\text{del}}^{i}$-values for the linear fit and 90% confidence intervals from our measured directional bias. On the same plot, we shade the reported localization profile of Kv4.2 immunogold-tagged particles from Kerti et al.’s 2012 (17) study. Our model predicts an asymmetric profile of $d_{\text{del}}^{i}$-values that falls within a standard deviation of localization data. Together, these results provide an account of how a previously unexplained and highly organized protein expression pattern can emerge from relatively simple transport mechanisms.

### A summary of the relationships between *mt* and *del* cargo densities

**Figure 7:**
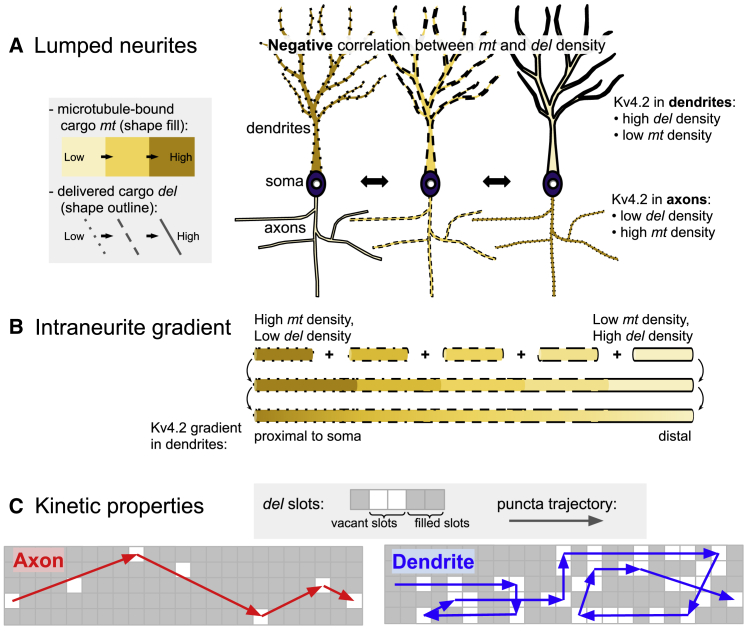
A summary of the relationships between microtubule-bound (*mt*) and delivered (*del*) cargo densities. The negative correlation between *mt* and *del* holds in lumped neurites (*A*) and along intraneurite gradients (*B*). (*C*) The kinetic properties of individual puncta trajectories in *mt* also reflects cargo demand in *del*. To see this figure in color, go online.

The questions we address in this study concern how densities of actively trafficked cargo (*mt*) relate to delivered localization (*del*). We lastly provide a summary of these relationships. A cartoon depicting *mt* and *del* is shown in Fig. 7, where *mt* and *del* densities are depicted by the shading and outline of the cell, respectively.

We depict the negative correlation between *mt* and *del* in lumped neurites in Fig. 7 *A*. As *mt* density in a neurite increases, we expect a corresponding decrease in *del* and vice versa. In our measurements, the intracellular distribution of Kv4.2 most closely resembles the rightmost cartoon (Fig. 7 *A*), as labeled. We next depict the negative correlation between *mt* and *del* along a gradient within a single neurite in Fig. 7 *B*. We find that the *mt* density of Kv4.2 decreases with dendritic distance, which is sufficient to explain the well-established *del* profile. An intuitive explanation for these negative relationships (Fig. 7, *A* and *B*) correlates cargo demand in *del* to local interactions that sequester and deplete passing cargo in *mt*.

Lastly, the kinetic properties of trafficked cargo in *mt* partially reflect the cargo demand in *del* (Fig. 7 *C*). Transport in axons is mechanistically distinct such that cargo is trafficked efficiently through regions of low, sparse demand with direct, unidirectional trajectories. Increased cargo demand in dendrites results in diffusive, winding, and meandering trajectories.

## Discussion

In this study, we measured densities of delivered Kv4.2 in synapses (*del*). We also analyzed mobile subunits (*mt*) in bleached neurites to estimate basal trafficking frequencies. We found substantially higher Kv4.2 subunit trafficking on *mt* in axons than in dendrites (Fig. 2). The *mt* distribution does not match established Kv4.2 functional or localization profiles (*del*, Fig. 1). However, just as a satellite photo of car traffic might reveal the highest density of cars on freeways as opposed to parked at a destination, our analysis showed that our measurements are consistent with a mass-action model of transport (Fig. 5). This implies that increased dendritic demand and local interactions with mobile cargo depletes dendritic microtubule-bound subunit density (*mt*). In axons, low subunit demand can result in higher trafficking density (*mt*). Indeed, previous localization studies, as well as our own observations, reveal a non-negligible density of axonal Kv4.2 (17,20). With no known presynaptic function, this axonal fraction might be an artifact of mass action, as our study suggests. We stress that although this paradoxical regime is consistent with standard trafficking models, the inverse relationship may not hold in all situations. Trivially, if transported cargo is strongly filtered from a compartment where it is not expressed, then trafficked density and expressed density may not show inverse relationships.

We also observed proximal-to-distal trends in dendritic Kv4.2 expression, particularly in puncta frequency (*mt*) and directional bias (Fig. 6). When these parameters constrain the rates of a mass-action model, the resultant delivered subunit density (*del*) can account for its well-established, characteristic functional profile (16,17). A similar increasing profile also exists for hyperpolarization-activated cyclic nucleotide-gated channels (55). Moreover, a study of hyperpolarization-activated cyclic nucleotide-gated channel trafficking and surface expression reveals similar dendritic trafficking dynamics to those reported here but no measured kinetic trend with distance along the dendrite (56). We suspect that the distance-dependent trafficking parameters observed here are partial contributors to the functional expression profiles of Kv4.2 channels, which likely rely heavily on local interactions with membrane protein complexes, in line with the sushi belt model. Other mechanisms for supporting distal dendritic expression include elegant passive mechanisms that exploit differences between volume and surface-confined diffusion (57).

Transport kinetics of Kv4.2 puncta differed quantitatively in axons and in dendrites (Fig. 3). Puncta in axons showed increased superdiffusivity, with increased net displacement, increased velocity, and decreased stall time. The opposite is observed in dendrites. This relationship makes sense physiologically; there is greater sequestration (higher *p*_off_) in dendrites, presumably because of local interactions with Kv4.2 cargo. However, increased microtubule offloading only partially explains the observed differences in kinetics. A random walk with memory better characterizes the observed stall fraction and diffusivity. We therefore infer that transport in axons is mechanistically different, with microtubule configuration or motor composition increasing the likelihood of unidirectional runs.

A number of implicit assumptions are made in our modeling. Notably, microtubule orientation is not considered in mass-action or stochastic simulations. Axons have a uniform arrangement of “plus-end-out” microtubules, whereas dendritic orientation is mixed. However, the microtubule motors are also mixed, with both kinesins and dyneins present in all neurites. Our understanding of Kv4.2 interaction with microtubule motors is incomplete, with only Kif17 identified as having a role in subunit trafficking (58). Without a comprehensive understanding of all motors and localization mechanisms, we assume the molecular “tug of war” between motors is equivalent in dendrites and axons, with no bias for microtubule orientation.

The models used in this study are an approximation of the molecular mechanisms known to underly trafficking. We briefly review the transport and expression mechanisms of Kv4.2 lumped within our models. Kv4.2 interacts with kinesin Kif17, suggesting transport on microtubules. In the absence of Kif17, Kv4.2 fails to localize in dendrites (58). Deletion of a portion of the C-terminus or fusion with myosin Va restricts expression of Kv4.2 to the somatodendritic region (51,59,60). Further, Kv channel-interacting proteins (KChIPs) have been established as auxiliary subunits that promote Kv4.2 exit from the endoplasmic reticulum for surface expression (4,22,61). An auxiliary subunit, DPP6, attached to Kv4.2 by a transmembrane domain (62), assists in trafficking Kv4.2 out of the endoplasmic reticulum to the plasma membrane (63). Dumenieu et al. (64) summarize these results with the following working hypothesis: Kv4.2 is trafficked short distances such as to proximal dendrites or within spines on actin filaments via myosin Va, whereas long-range transport is mediated along microtubules via KChIPs and Kif17.

There are unavoidable methodological tradeoffs between attempting to quantify protein at physiologically low expression levels and inducing high expression that enables live imaging. We assumed that the transport behavior of the transfected construct Kv4.2-SGFP2 is similar to that of endogenously expressed subunits. Our results are therefore subject to this caveat. It is possible that transfection of a recombinant construct alters intracellular expression profiles. For this reason, we validated expression profiles by labeling and quantifying both endogenous and transfected Kv4.2 subunits while using a construct that has been thoroughly compared to endogenous channel (28). We anticipate that our approach can spur future work that will mitigate experimental challenges by designing enhanced fluorescent probes that might be suited to live superresolution imaging. Such methods will be crucial for peering deeper into the logic of intracellular protein regulation.

## Author contributions

A.B. and T.O. designed the study. A.B., L.L., R.P., and Y.-X.W. carried out all experiments. A.B. performed numerical simulations. J.M. contributed the construct and experiment support. A.B., J.M., D.H., and T.O. designed experiments. A.B., J.M., L.L., R.P., and T.O. analyzed results. A.B. and T.O. wrote the manuscript. All authors discussed the results and commented on versions of the manuscript.

## Supporting material

Document S1. Supporting materials and methods, Figs. S1–S9, and Table S1

Document S2. Article plus supporting material
