# Supplementary material for: Paradoxical relationships between active transport and global protein distributions in neurons
Source: Biophys J. 2021 Apr 2;120(11):2085–101. doi: 10.1016/j.bpj.2021.02.048 (PMC8390833; doi:10.1016/j.bpj.2021.02.048)
Supplement: Document S1. Supporting materials and methods, Figs. S1–S9, and Table S1 [file mmc1.pdf]

Biophysical Journal, Volume *120*

Supplemental information

Paradoxical relationships between active transport and global protein distributions in neurons

Adriano Bellotti, Jonathan Murphy, Lin Lin, Ronald Petralia, Ya-Xian Wang, Dax Hoffman, and Timothy O'Leary

Paradoxical relationships between active transport and global protein distributions in neurons - Supplementary Material

A. Bellotti, J. Murphy. L. Lin, R. Petralia,
Y-X Wang, D. Hoffman, T. O'Leary

March 18, 2021

Supplementary Material

An online supplement to this article can be found by visiting BJ Online at http://www.biophysj.org.

Derivation of drift-diffusion equation

Here we derive the drift-diffusion equation in one dimension from a random walk.

Random walk master equation

We begin with a one dimension line that is discretized in space with index $x = 1, 2, ..., X$ and in time with index $n = 1, 2, ..., N$. We are observing a random walk, where a random walker has a probability of f that a step is taken to the right (forwards) and b that a step is taken to the left (backwards). Each step therefore increases or decreases space by one distance unit Δx ($x \to x - \Delta x$ or $x \to x + \Delta x$, respectively). Each step also increases time by one time unit ($n \to n + \Delta n$) where Δn is the duration of one time step.

Let $P_n(x)$ be the probability of finding the walker at position x at some time n. We can now write a master equation for the walker occupying position x at time $n + \Delta n$:

$$P_{n+1}(x) = fP_n(x - \Delta x) + bP_n(x + \Delta x) + (1 - f - b)P_n(x) \tag{1}$$

Notice that we allow for the probability that the walker stays in place with probability $1 - f - b$. If we distribute the last term on the RHS, we have

$$P_{n+\Delta n}(x) = fP_n(x - \Delta x) + bP_n(x + \Delta x) + P_n(x) - fP_n(x) - bP_n(x)$$

Subtract the term $P_n(x)$ from both sides.

$$P_{n+\Delta n}(x) - P_n(x) = fP_n(x-\Delta x) + bP_n(x+\Delta x) - bP_n(x) - fP_n(x) \tag{2}$$

We leave the LHS of Equation 2 as it is now before converting to continuous time and space after working on the RHS.

The RHS of Equation 2 is algebraically modified to a form related to the finite difference approximations of the first and second spatial derivatives. In order to do this, the first and second terms of the RHS of Equation 2 are expanded as follows:

$$pP_n(x-\Delta x) = \frac{1}{2}fP_n(x-\Delta x) - -\frac{1}{2}pP_n(x-\Delta x)$$

$$bP_n(x+\Delta x) = \frac{1}{2}bP_n(x+\Delta x) - -\frac{1}{2}bP_n(x+\Delta x)$$

Additionally, the following terms summing to zero are added to the RHS of Equation 2

$$\frac{1}{2}fP_n(x+\Delta x) - \frac{1}{2}fP_n(x+\Delta x) + \frac{1}{2}bP_n(x-\Delta x) - \frac{1}{2}bP_n(x-\Delta x)$$

All terms are added to Equation 2 and expansions are substituted into Equation 2. Rearrangement of terms and factoring puts the equation in the desired form, as follows:

$$\begin{aligned}
P_{n+\Delta n}(x) - P_n(x) &= fP_n(x-\Delta x) + bP_n(x+\Delta x) - fP_n(x) - bP_n(x) \\
&= \frac{1}{2}fP_n(x-\Delta x) - fP_n(x) + \frac{1}{2}pP_n(x+\Delta x) + \frac{1}{2}bP_n(x-\Delta x) - bP_n(x) \\
&\quad + \frac{1}{2}bP_n(x+\Delta x) - \frac{1}{2}fP_n(x+\Delta x) + \frac{1}{2}fP_n(x-\Delta x) + \frac{1}{2}bP_n(x+\Delta x) \\
&\quad - \frac{1}{2}bP_n(x-\Delta x) \\
&= \frac{1}{2}\Big(fP_n(x-\Delta x) - 2fP_n(x) + fP_n(x+\Delta x) + bP_n(x-\Delta x) - 2bP_n(x) \\
&\quad + bP_n(x+\Delta x)\Big) - \frac{1}{2}\Big(fP_n(x+\Delta x) - fP_n(x-\Delta x) - bP_n(x+\Delta x) \\
&\quad + bP_n(x-\Delta x)\Big) \\
P_{n+\Delta n}(x) - P_n(x) &= \frac{1}{2}(f+b)\Big(P_n(x-\Delta x) - 2P_n(x) + P_n(x+\Delta x)\Big) \\
&\quad - \frac{1}{2}(f-b)\Big(P_n(x+\Delta x) - P_n(x-\Delta x)\Big)
\end{aligned} \tag{3}$$

The LHS of Equation 3 will become the time derivative of P, and the first and second terms on the RHS will become second and first spatial derivatives of P, respectively.

Finite difference approximation

As the spatial and temporal step sizes Δx and Δn approach zero, Equation 3 approaches the continuous drift-diffusion equation. In order to approximate the form of the derivatives in continuous space and time, we can use the finite difference approximations for first and second derivatives. We begin with the forward difference equation for a first derivative of some arbitrary function $f(t)$:

$$\frac{df}{dt} = \frac{f(t + \Delta t) - f(t)}{\Delta t} \tag{4}$$

We can also consider the central difference equation for the first derivative of $f(t)$:

$$\frac{df}{dt} = \frac{f(t + \frac{1}{2}\Delta t) - f(t - \frac{1}{2}\Delta t)}{\Delta t}$$

Since the smallest step size in our discrete case was Δt and not $\frac{1}{2}\Delta t$, it might be more helpful to take a central difference approximation over two steps $2\Delta t$:

$$\frac{df}{dt} = \frac{f(t + \Delta t) - f(t - \Delta t)}{2\Delta t} \tag{5}$$

The central difference approximation can then be applied to a second derivative using the chain rule:

$$\frac{d^2 f}{dt^2} = \frac{\frac{d}{dt} f(t + \frac{1}{2}\Delta t) - \frac{d}{dt} f(t - \frac{1}{2}\Delta t)}{\Delta t}$$

$$\frac{d^2 f}{dt^2} = \frac{1}{\Delta t}\Big(\frac{f(t + \Delta t) - f(t)}{\Delta t} - \frac{f(t) - f(t - \Delta t)}{\Delta t}\Big)$$

$$\frac{d^2 f}{dt^2} = \frac{f(t + \Delta t) - 2f(t) + f(t - \Delta t)}{\Delta t^2} \tag{6}$$

The approximations in Equations 4, 5, and 6 can be used to write discrete difference equations as continuous derivatives.

Discrete to continuous space and time

In order to modify our master equation in Equation 3 to continuous space and time, all terms must be in the form of Equations 4, 5, or 6. We can multiply the LHS by unit value $\frac{\Delta n}{\Delta n}$, the first term on the RHS by unit $\frac{\Delta x^2}{\Delta x^2}$, and the second

term on the RHS by unit $\frac{\Delta x}{\Delta x}$:

$$\begin{aligned}
\Big(P_{n+\Delta n}(x) - P_n(x)\Big)\frac{\Delta n}{\Delta n} &= \frac{1}{2}\frac{\Delta x^2}{\Delta x^2}(f+b)\Big(P_n(x-\Delta x) - 2P_n(x) + P_n(x+\Delta x)\Big) \\
&\quad - \frac{1}{2}\frac{\Delta x}{\Delta x}(f-b)\Big(P_n(x+\Delta x) - P_n(x-\Delta x)\Big) \\
\frac{P_{n+\Delta n}(x) - P_n(x)}{\Delta n}\Delta n &= \frac{\Delta x^2}{2}(f+b)\frac{P_n(x-\Delta x) - 2P_n(x) + P_n(x+\Delta x)}{\Delta x^2} \\
&\quad - \frac{\Delta x}{2}(f-b)\frac{P_n(x+\Delta x) - P_n(x-\Delta x)}{\Delta x} \\
\frac{P_{n+\Delta n}(x) - P_n(x)}{\Delta n} &= \frac{\Delta x^2(f+b)}{2\Delta n}\frac{P_n(x-\Delta x) - 2P_n(x) + P_n(x+\Delta x)}{\Delta x^2} \\
&\quad - \frac{\Delta x(f-b)}{\Delta n}\frac{P_n(x+\Delta x) - P_n(x-\Delta x)}{2\Delta x}
\end{aligned}$$

We can now make derivative approximations using Equations 4, 5, and 6 and replace discrete time n and space x with continuous time t and space x.

$$\frac{\partial P(x,t)}{\partial t} = D\frac{\partial^2 P(x,t)}{\partial x^2} + v\frac{\partial P(x,t)}{\partial x}$$

where $P(x,t)$ is the probability of finding a random walker at position x at time t in continuous space and time. We have also identified $D = \frac{\Delta x^2(f+b)}{2\Delta n}$ and $v = \frac{\Delta x(f-b)}{\Delta n}$ as $\lim_{\Delta x \to 0}$ and $\lim_{\Delta n \to 0}$. For a population that contains a total of T random walkers, the concentration of particles in some segment of line can be defined as $c(x,t) = TP(x,t)/\Delta x$ using the law of large numbers. Substituting this into our equation produces the one-dimensional drift-diffusion equation for a large population of particles in a more recognizable form:

$$\frac{\partial c(x,t)}{\partial t} = D\frac{\partial^2 c(x,t)}{\partial x^2} + v\frac{\partial c(x,t)}{\partial x} \tag{7}$$

Distribution of kinetic measures in stochastic model

In this section, the stochastic model is analyzed as a modified discrete time random walk. We aim to realize the distributions of the experimental measures of puncta kinetics (Figure 5C). Among the distributions derived here are distance traveled and mean speed.

Total distance traveled

Until absorption, a puncta in this stochastic model behaves as in a one-dimensional unbiased random walk. We use this premise to solve for the distributions of the kinetic measures of interest.

We begin with total distance traveled D_{tot}, which is a measure of the puncta's final position relative to its origin. The final position is the site of absorption, which ends the random walk. The expected value of an unbiased

random walk is 0, regardless of the number of time steps n. For a biased random walk with rightward (+1) propensity f and leftward (-1) propensity b, the expected value after n time steps is $n(f-b)$. The variability around this expected value scales with $\sqrt{n}$. As the number of puncta simulated approaches infinity, the distribution of their final positions - by central limit theorem - approximates a bell curve. In a DTRW with no absorption, this is well approximated by a normal distribution. We also find that unidirectional runs (with increasing parameter p_{mem}) increase the the standard deviation σ of the normal distribution approximation [1, 2].

In a DTRW with absorption (also called decay, degradation, sink, etc.), the shape of the distribution changes. To estimate the total distance traveled by puncta on a 1D lattice with absorption, we use a differential equation for diffusion. A random walk with more than a few steps is well approximated by diffusion [1]. The PDE for diffusion with decay and source is

$$\frac{\partial c}{\partial t} = D\frac{\partial^2 c}{\partial x^2} - K_{\mathrm{off}}c + s \tag{8}$$

where $c = c(x,t)$ is the concentration of puncta at some position x at time t, D is the diffusion coefficient, K_{off} is the absorption rate, and s is puncta source at $x = 0$. The distribution of net displacement of puncta is approximated by the steady-state ($\frac{\partial c}{\partial t} = 0$) of Eq. 8:

$$0 = D\frac{\partial^2 c}{\partial x^2} - K_{\mathrm{off}}c$$

Solving this produces two exponentials of the form:

$$c(x) = Ae^{\lambda x} + Be^{-\lambda x} \tag{9}$$

where λ is a space constant for spread of puncta before absorption: $\lambda = \sqrt{K_{\mathrm{off}}/D}$. If we next impose the restriction that $c \to 0$ as $x \to \pm\infty$, then Eq. 9 is restricted to

$$\begin{aligned} c(x) &= Ae^{\lambda x} && \text{for } x < 0 \\ c(x) &= Be^{-\lambda x} && \text{for } x > 0 \end{aligned}$$

For continuity at $c(0)$, $A = B$. Solving for this single coefficient $A = B$ requires the amount of mass M released at the source at $c = 0$. This quantity M in units of quantity per cross sectional area per time is split into left and right directions, therefore

$$A = B = \frac{M}{2D\lambda} = \frac{M}{2\sqrt{DK_{\mathrm{off}}}}$$

. The full symmetric solution is

$$\begin{aligned} c(x) &= \frac{M}{2\sqrt{DK_{\mathrm{off}}}} \exp \sqrt{K_{\mathrm{off}}/D}x && \text{for } x < 0 \\ c(x) &= \frac{M}{2\sqrt{DK_{\mathrm{off}}}} \exp -\sqrt{K_{\mathrm{off}}/D}x && \text{for } x > 0 \end{aligned}$$

In our experiments, the orientation of axons was not always clear. We therefore report this measure as distance traveled rather than net displacement, since we can only account for magnitude of displacement and not direction. The distribution for this measure of total distance traveled is thus symmetric about the y-axis:

$$c(x) = \frac{M}{\sqrt{DK_{\text{off}}}} \exp -\sqrt{K_{\text{off}}/D}x \qquad \text{for } x > 0 \tag{10}$$

For a DTRW, the diffusion coefficient can be approximated as $D = 2(\Delta x)^2 p_+ p_- / \Delta t$, where Δx and Δt are the discrete steps in space and time, and p_+ and p_- are right and left jump probabilities [3]. The total punctal distance traveled as computed in our experimental kinetic measure is follows a distribution with the form of Eq. 10, a monotonic decreasing function.

Average speed

The next kinetic measure for which we derive a probability distribution is average speed v. As a kinetic measure, v is computed as distance traveled divided by total puncta run time.

As before, we assume that a DTRW with more than a few steps is well approximated by diffusion [1]. We can then solve the diffusion equation

$$\frac{\partial c}{\partial t} = D \frac{\partial^2 c}{\partial x^2}$$

for diffusion propagator $c(x,t)$

$$c(x,t) = \frac{1}{\sqrt{4\pi Dt}} \exp\Big(- \frac{x^2}{4Dt} - K_{\text{off}} t \Big)$$

We use $c(x,t)$ to compute the probability of puncta at position x at time t: $p(x|t)$.

$$\begin{aligned} p(x|t) &= \frac{K_{\text{off}} c(x,t)}{\int_{-\infty}^{\infty} K_{\text{off}} c(x,t) dx} \\ &= \frac{K_{\text{off}} \frac{1}{\sqrt{4\pi Dt}} \exp\Big(- \frac{x^2}{4Dt} - K_{\text{off}} t \Big)}{\int_{-\infty}^{\infty} K_{\text{off}} \frac{1}{\sqrt{4\pi Dt}} \exp\Big(- \frac{x^2}{4Dt} - K_{\text{off}} t \Big) dx} \end{aligned}$$

We cancel terms in the numerator and denominator and solve the integral:

$$\begin{aligned} p(x|t) &= \frac{\exp\Big(- \frac{x^2}{4Dt} \Big)}{\int_{-\infty}^{\infty} \exp\Big(- \frac{x^2}{4Dt} \Big) dx} \\ &= \frac{\exp - \frac{x^2}{4Dt}}{\sqrt{4\pi Dt}} \end{aligned}$$

Note that $p(x|t)$ is independent of K_{off}

With $p(x|t)$, we can now compute the probability of a given puncta velocity v at time t: $p(v|t)$

$$p(v|t) = \int_{-\infty}^{\infty} \delta(v - \frac{|x|}{t}) p(x|t) dx$$

where δ is the Dirac delta function. This integral sweeps through all positions x to find the probability of puncta at least location ($p(x|t)$) that matches each speed ($\delta(v - \frac{|x|}{t})$. We proceed using the scaling, symmetry, and translation properties of the Dirac delta function:

$$\begin{aligned}
p(v|t) &= \int_{-\infty}^{\infty} \delta(v - \frac{|x|}{t}) p(x|t) dx \\
&= \int_{-\infty}^{\infty} \delta\big(\frac{1}{t}(vt - |x|)\big) p(x|t) dx \\
&= \int_{-\infty}^{\infty} t\delta(vt - |x|) p(x|t) dx \\
&= t\int_{-\infty}^{\infty} \delta(vt - |x|) p(x|t) dx \\
&= t\Big(p(\delta t|t) + p(-\delta t|t)\Big) \\
&= \frac{2t}{\sqrt{4\pi D t}} e^{-(vt)^2/4Dt} \\
p(v|t) &= \frac{t}{\sqrt{\pi D}} e^{-v^2 t/4D}
\end{aligned}$$

To check this probability distribution, we integrate it over the entire domain to ensure it sums to 1:

$$\int_0^{\infty} p(v|t) dv = \frac{1}{2}\int_{-\infty}^{\infty} p(v|t) dv = \frac{1}{2}\sqrt{\frac{t}{\pi D}} \int_{-\infty}^{\infty} \exp -\frac{v^2 t}{4D} dv = \frac{1}{2}\sqrt{\frac{t}{\pi D}}\sqrt{\frac{4\pi D}{t}} = 1$$

We next compute the expected time until absorption $\phi(t)$, which is approximated as

$$\phi(t) = K_{\text{off}} e^{-K_{\text{off}} t}$$

With $p(v|t)$ and $\phi(t)$, we can now compute the probability distribution for average speed $F(v)$. We integrate the velocity distribution at a fixed time

$(p(v|t))$ multiplied by the fraction of particles $(\phi(t))$ for all time $t > 0$:

$$
\begin{aligned}
F(v) &= \int_0^\infty p(v|t)\phi(t)dt \\
&= \int_0^\infty \Big(\frac{t}{\sqrt{\pi D}} e^{-v^2 t/4D}\Big)\Big(K_{\text{off}} e^{-K_{\text{off}} t}\Big) dt \\
&= \frac{K_{\text{off}}}{\sqrt{\pi D}} \int_0^\infty \sqrt{t} e^{-(v^2/4D+k)t} dt \\
&= \frac{K_{\text{off}}}{\sqrt{\pi D}} \frac{\sqrt{\pi}}{2} (\frac{v^2}{4D} + K_{\text{off}})^{-3/2} \\
&= \frac{K_{\text{off}}}{2\sqrt{D}(\frac{v^2}{4D} + K_{\text{off}})^{-3/2}}
\end{aligned}
$$

If we substitute $v_0 = 2\sqrt{DK_{\text{off}}}$,

$$
F(v) = \frac{1}{v_0}\Big(\frac{v^2}{v_0^2} + 1\Big)^{-3/2} \tag{11}
$$

To confirm our result, we can compute its integral for all $v \geq 0$:

$$
\int_0^\infty F(v)dv = 1
$$

Like $c(x)$, $F(v)$ is a monotonically decreasing function. Increasing D and/or K_{off} increases the tailedness of the distribution. We can compute the expected value of mean particle speed $\langle v \rangle$ as follows:

$$
\langle v \rangle = \int_0^\infty vF(v)dv = v_0 = 2\sqrt{DK_{\text{off}}}
$$

This shows that $\langle v \rangle$ increases with increasing D and/or K_{off}

Statistical inference using stochastic model

Here we describe our method for statistical inference of puncta behavior based on experimental observations. In broad terms, we estimate parameters p_{off} and p_{mem} from our stochastic model using the observed distributions of kinetic measurements.

We interpret our experimental measures of puncta kinetics (Figure 5C) as evidence of puncta behavior. We have developed a stochastic model of puncta transport based on a modified random walk (Figure S8A), consistent with observed intracellular transport [4, 5, 6]. Our model produces simulated data similar to our experiments (compare Figures S7 and S8). We aim to infer whether the observed differences between axons and dendrites can result from a difference in p_{off} or p_{mem}. To this end, we perform a model fit to data from axons and dendrites. We then compare a model fit using p_{off} alone as well as with p_{off} and p_{mem}.

Maximum likelihood estimation

Maximum likelihood estimation (MLE) is a standard method of estimating the parameters of a model such that the model's output is the most probable match to some observed data. In this method, a likelihood equation is derived from the joint probability distribution of simulated and observed data as a function of model parameters. The set of parameters that maximizes the likelihood equation produces the best fit between the simulated and observed data.

We first explore the direct estimation of p_{off} and p_{mem} for which our stochastic model most closely reproduces our observed data. We then estimate parameters that reproduce a distribution that estimates our observed data—a less direct but simpler method.

We begin by defining the likelihood function:

$$\mathcal{L}(p_{\text{off}}, p_{\text{mem}} \mid \text{observed data}) = P(\text{transport producing observed data} \mid p_{\text{off}}, p_{\text{mem}}) \tag{12}$$

where the likelihood $\mathcal{L}$ of parameters $p_{\text{off}}, p_{\text{mem}}$ producing the observed data is equal to the probability of the actual physical transport mechanism producing the observed data given those parameters $p_{\text{off}}, p_{\text{mem}}$. Defining the probability in Eq. 12 is difficult for a few reasons. First, the observed data consists of sets of net displacements δ, mean speeds s, stall fractions ϵ, and diffusivities α for $N_a = 961$ puncta in axons and $N_d = 507$ puncta in dendrites:

$$\delta_1, \delta_2, ..., \delta_{N_a-1}, \delta_{N_a} \qquad \delta_1, \delta_2, ..., \delta_{N_d-1}, \delta_{N_d} \tag{13}$$

$$s_1, s_2, ..., s_{N_a-1}, s_{N_a} \qquad s_1, s_2, ..., s_{N_d-1}, s_{N_d} \tag{14}$$

$$\epsilon_1, \epsilon_2, ..., \epsilon_{N_a-1}, \epsilon_{N_a} \qquad \epsilon_1, \epsilon_2, ..., \epsilon_{N_d-1}, \epsilon_{N_d} \tag{15}$$

$$\alpha_1, \alpha_2, ..., \alpha_{N_a-1}, \alpha_{N_a} \qquad \alpha_1, \alpha_2, ..., \alpha_{N_d-1}, \alpha_{N_d} \tag{16}$$

This observed data is a large combination of variables. Further, these variables (Eqs. 13-16) are not measured directly; they are computed from the physical coordinates of trajectories from individual puncta. Computing s, ϵ, and α involves the trajectories themselves, not just the final position. The random process in our model is time-dependent (non-stationary) and non-ergodic, so deriving the statistics (expected value and variance) does not reveal the time averages. The expected value itself would only be useful in computing net displacements (Eq. 13), since this is the only kinetic measure computed using solely outcome of the random process without the full trajectory. Since a key parameter of our system p_{mem} abruptly ends the random process, the statistics are not as simple as those of a standard random walk. These nontrivial derivations only produce the likelihood function, which then requires differentiation w.r.t. p_{off} and p_{mem} for an analytical solution to this problem. For these reasons, rather than deriving the statistics and time-averages of our model, we opt for a numerical approach to this problem.

Instead of deriving equations for δ, s, ϵ, and α, we can estimate these probability distributions of these kinetic measures by averaging the behavior of simulated puncta. Simulating a large number of puncta N_s is crucial given the

stochasticity of the model. From N_s simulated trajectories with a given p_{off} and p_{mem}, we can then compute:

$$\delta_1, \delta_2, ..., \delta_{N_s-1}, \delta_{N_s} \tag{17}$$

$$s_1, s_2, ..., s_{N_s-1}, s_{N_s} \tag{18}$$

$$\epsilon_1, \epsilon_2, ..., \epsilon_{N_s-1}, \epsilon_{N_s} \tag{19}$$

$$\alpha_1, \alpha_2, ..., \alpha_{N_s-1}, \alpha_{N_s} \tag{20}$$

With a sufficiently large N_s, we obtain averages of δ, s, ϵ, and α. This method follows the law of large numbers, whereby the average of a large number of iterations approaches the expected behavior of the random process. We can then compare simulated data (Eqs. 17-20) to observed data (Eqs. 13-16) and optimize for parameter set p_{off} and p_{mem} that minimizes this variation. This semi-empirical approach employs both model simulation to estimate kinetic distributions and optimization to minimize the difference between simulated and observed data.

MLE is still applicable in this approach. Approximating simulated and observed data as probability distributions simplifies the optimization problem. Both observed and simulated sample sets can be approximated as probability distributions, and the distance between distribution parameters is minimized. MLE is a suitable method for fitting observed and simulated data to a distribution with a defined probability density function (pdf).

The pdf of two kinetic measures δ (Eq. 10) and s (Eq. 11) are derived in the previous section. Both are monotonically decreasing functions with domain $[0, \infty)$. The pdf of the other measures ϵ and α are seemingly intractable and beyond the scope of this paper. We therefore approximate the distributions of kinetic measures using an estimate likelihood with the gamma distribution. The gamma distribution can accommodate the monotonic decreasing pdfs derived here and is flexible for all simulated and observed data (Eqs. 17-20 and Eqs. 13-16 depicted in Figure 5C). The gamma distribution is appropriate because it is continuous and covers a semi-infinite $[0, \infty)$ interval. A gamma distribution has shape k and scale θ, which are fit to both observed and simulated data.

To this end, we derive a function for the likelihood of the gamma distribution with parameters k and θ producing observed or simulated data. As an example, we estimate k and θ that best match the observed mean speed s in axons:

$$\mathcal{L}(k, \theta \mid X = s_1, ..., s_{N_a}) = P(\text{gamma distribution fits X} \mid k, \theta)$$

Here, the probability in the RHS is, by definition, the pdf of the gamma distribution:

$$\mathcal{L}(k, \theta \mid X = s_1, ..., s_{N_a}) = \frac{1}{\Gamma(k)\theta^k} X^{k-1} e^{x/\theta} \tag{21}$$

where vector X is the data to which a gamma distribution is fit. The RHS of Eq. 21 is the pdf of the gamma distribution, where Γ is the gamma function: $\Gamma(g) = \int_0^\infty z^{g-1} e^{-z} dz$.

The remaining steps for MLE involve calculating the log-likelihood $\ell(k, \theta \mid X)$, taking partial derivatives w.r.t. each parameter $\partial\ell(k,\theta)/\partial k$ and $\partial\ell(k,\theta)/\partial\theta$, setting to zero, and solving for both k and θ. The solution for MLE using the gamma distribution has no closed-form expression [7, 8]. Rather, a numerical solution is computed. We use MATLAB function `fitdist`, which solves the following system of two equations:

$$\ln k - \psi(k) = \ln\left(\frac{(\sum_{i=1}^{N_a} s_i)/N_a}{(\prod_{i=1}^{N_a} s_i)^{1/N_a}}\right) \quad , \quad \theta = \frac{(\sum_{i=1}^{N_a} s_i)/N_a}{k}$$

where ψ is the digamma function: $\psi(g) = \frac{d}{dx}\ln(\Gamma(g))$.

MLE for gamma distribution parameters is performed for each set of observed data (Eqs. 13-16) as well as for N_s trajectories simulated using the stochastic model (Eqs. 17-20) with a given p_{off} and p_{mem}. Observed data for net displacement and average speed are normalized between 0 and 1. All resultant gamma parameters are:

$$k_{\delta,a}, \theta_{\delta,a} \qquad k_{s,a}, \theta_{s,a} \qquad k_{\epsilon,a}, \theta_{\epsilon,a} \qquad k_{\alpha,a}, \theta_{\alpha,a} \tag{22}$$

$$k_{\delta,d}, \theta_{\delta,d} \qquad k_{s,d}, \theta_{s,d} \qquad k_{\epsilon,d}, \theta_{\epsilon,d} \qquad k_{\alpha,d}, \theta_{\alpha,d} \tag{23}$$

$$k_{\delta,s}, \theta_{\delta,s} \qquad k_{s,s}, \theta_{s,s} \qquad k_{\epsilon,s}, \theta_{\epsilon,s} \qquad k_{\alpha,s}, \theta_{\alpha,s} \tag{24}$$

where each variable k or θ denotes gamma shape or scale parameter, the first subscript denotes kinetic measure, and the second subscript denotes source of the distribution (axons, dendrites, or simulation). The gamma distributions of kinetic measures in axons and dendrites (Eqs. 22 and 23) are plotted over the raw data in Figure 5C. We now have a succinct description of each distribution using two rather than N_a, N_d, or N_s terms. This framework allows us to compare observed and simulated data. We next use a least squares method fit simulation parameters p_{off} and p_{mem} to the experimental data.

Least squares method of optimization

MLE allows for a representation of data from axons, dendrites, or simulations as a gamma distribution using only two parameters k and θ. We now aim to fit our stochastic model to experimental data. A standard approach in model fitting is the least squares method (LSM) of regression. Here, we describe LSM in the context of our problem. We then outline numerical algorithms for solving nonlinear LSM problems.

We describe a model M with output y as a function of independant variable x and adjustable parameters β:

$$y = M(x, \beta)$$

We substitute an example optimization problem using our data and parameters,

$$[k_{\delta,s}, \theta_{\delta,s}] = M(p_{\text{off}}, p_{\text{mem}}) \tag{25}$$

where M is the stochastic model, p_{off} and p_{mem} are the adjustable parameters that are tweaked for an optimal fit with output $y = [k_{\delta,s}, \theta_{\delta,s}]$. Model fitting can be applied to any number of outputs from Eq. 24. Note that our function for optimization in Eq. 25 differs from that in curve fitting in that it takes no independent variables x. The model fitting in our example fits only the model parameters. Further, M itself is a complex, nonlinear function. Contained within M is a simulation of the N_s puncta trajectories using the stochastic model (Figure S8). Also contained within M is computation of kinetic measures (δ, s, ϵ, and α) for each of the N_s trajectories. Lastly contained within M is the MLE of the gamma parameters (k and θ) from the distributions of kinetic measures (Eqs. 17-20). Selection of N_s is therefore a balance between computational cost (run time) and accuracy of expected puncta behavior (by law of large numbers). We choose $N_s = 10,000$ puncta in each iteration of M.

The quality of fit for the output of M is measured by the size of the residuals, or the difference between the observed data and estimated data:

$$\text{residuals} = \text{observed data} - \text{simulated data}$$

In the context of our problem, we will fit the output in Eq. 25 to observed data in axons:

$$\begin{aligned} r &= [k_{\delta,a}, \theta_{\delta,a}] - M(p_{\text{off}}, p_{\text{mem}}) \\ r &= [k_{\delta,a}, \theta_{\delta,a}] - [k_{\delta,s}, \theta_{\delta,s}] \end{aligned} \tag{26}$$

where $[k_{\delta,a}, \theta_{\delta,a}]$ is the axonal data, and r is a vector of residuals, $r = [r_1, r_2]$. The LSM aims to minimize the sum of the squares S of the residuals: $S = \sum r_i^2$. S is minimized by setting its gradient to zero. This involves taking a partial derivative of S w.r.t. each parameter. In our example:

$$\begin{aligned} \frac{\partial S}{\partial p_{\text{off}}} &= \frac{\partial}{\partial p_{\text{off}}} \sum_i r_i^2 = 0 & \frac{\partial S}{\partial p_{\text{mem}}} &= \frac{\partial}{\partial p_{\text{mem}}} \sum_i r_i^2 = 0 \\ &= 2\sum_i r_i \frac{\partial r_i}{\partial p_{\text{off}}} = 0 & &= 2\sum_i r_i \frac{\partial r_i}{\partial p_{\text{mem}}} = 0 \end{aligned}$$

Using Eq. 26 and given that the partial derivatives of $[k_{\delta,a}, \theta_{\delta,a}]$ w.r.t. p_{off} and p_{mem} is zero,

$$\frac{\partial S}{\partial p_{\text{off}}} = -2\sum_i r_i \frac{\partial M}{\partial p_{\text{off}}} = 0 \qquad \frac{\partial S}{\partial p_{\text{mem}}} = -2\sum_i r_i \frac{\partial M}{\partial p_{\text{mem}}} = 0 \tag{27}$$

A closed-form solution for Eq. 27, as in most non-linear least squares problems, does not exist. A numerical algorithm is instead used to minimize S.

There are several algorithms for nonlinear curve-fitting and data-fitting in the least squares sense. We use MATLAB function `lsqcurvefit`, which employs a damped LSM, also known as the Levenberg–Marquardt algorithm [9, 10]. The damped LSM is a combination of the Gauss–Newton algorithm with a trust region. A broad overview of the damped LSM is presented here.

The damped LSM is an iterative process that begins at a starting point for parameter vector β. For our system, $\beta = [p_{\text{off}}, p_{\text{mem}}]$. The aim is to find the set of β that best fits each of the m data points (x_i, y_i). Again, in our system, we have no independent variables x_i, and we fit outputs of model M to observed data y_i. In each step of the algorithm, β is updated with a new parameter estimate $\beta + \Delta$. To make a sensible modification Δ to the parameter estimate, the nonlinear function M is approximated by linearization (first-order approximation):

$$M(x_i, \beta + \Delta) \approx M(x_i, \beta) + J_i\Delta \qquad , \qquad J_i = \frac{\partial M(\beta)}{\partial \beta}$$

where J_i is the gradient of M w.r.t. β. Using this approximation, we can compute the sum of squares S of the residuals:

$$\begin{aligned} S(\beta + \Delta) &\approx \sum_i^m r_i^2 \\ S(\beta + \Delta) &\approx \sum_i^m \Big(y_i - M(x_i, \beta) - J_i\Delta\Big)^2 \end{aligned} \tag{28}$$

As before, Eq. 28 is minimized where its derivative equals zero. The derivative of Eq. 28 w.r.t. Δ is

$$(J^T J)\Delta = J^T\big(y - M(\beta)\big) \tag{29}$$

where J is a Jacobian matrix consisting of rows J_i. Matrix multiplication in Eq. 29 results in a system of linear equations that is solved for Δ. This procedure of linearization, function approximation, and solving for step Δ repeats to progress toward a minimum S. The algorithm stops when step size Δ falls below some preset threshold. The approach as described thus far is the Gauss-Newton method.

The damped LSM has the addition of damping factor λ, a non-negative scalar, as follows:

$$(J^T J + \lambda I)\Delta = J^T\big(y - M(\beta)\big) \tag{30}$$

where I is the identity matrix. When λ is small or zero, the method approximates the Gauss-Newton method. When λ is large, the direction of Δ approaches the direction of steepest descent but with magnitude approaching zero. λ adjusts the size of the step, defining a trust region around the current estimate β that is reevaluated at each iteration. If $S(\beta + \Delta) < S(\beta)$, a successful step toward a minima, λ is decreased and the trust region increased. If $S(\beta + \Delta) > S(\beta)$, λ is increased and the trust region decreased. In this regard, a dynamic λ allows for a search that mediates between steepest descent and the Gauss-Newton method. For instance, a limitation of the Gauss-Newton method arises when second-order terms dominant the gradient, since the Gauss-Newton method relies on first-order approximation. Dampening with λ can ensure descent path more efficient than searching for steepest descent.

We use this algorithm in a series of model fits to our experimental data. Given the stochastic nature of M in our system, we increase the lower threshold for the finite difference step size Δ. This ensures continuous progression toward a global minima despite slight variation in output with repeated evaluations of M. Otherwise, with a low minimum Δ, the algorithm greatly reduces the trust region and terminates at a false local minima—an artifact of the randomness in M.

We first fit

$$[k_{\delta,s}, \theta_{\delta,s}, k_{s,s}, \theta_{s,s}] = M(p_{\text{off}}, p_{\text{mem}} = 0)$$

to the corresponding experimental results in axons ($[k_{\delta,a}, \theta_{\delta,a}, k_{s,a}, \theta_{s,a}]$) and dendrites ($[k_{\delta,d}, \theta_{\delta,d}, k_{s,d}, \theta_{s,d}]$). p_{mem} is constrained to zero, and upper and lower bounds for p_{off} are set to 0 and 1, consistent with the range of a probability. The starting value was $p_{\text{off}} = 0.001$. The result of this fit is depicted in Figure 5C (*second column*). We then fit

$$[k_{\delta,s}, \theta_{\delta,s}, k_{s,s}, \theta_{s,s}, k_{\epsilon,s}, \theta_{\epsilon,s}] = M(p_{\text{off}}, p_{\text{mem}})$$

to the corresponding experimental results in axons and dendrites. We now fit both parameters, and both are bounded between 0 and 1 with starting values $p_{\text{off}} = 0.001$ and $p_{\text{mem}} = 0.01$. The result of this fit is depicted in Figure 5C (*third column*). The goodness of these fits and their implications are discussed in Results.

References

[1] Subrahmanyan Chandrasekhar. Stochastic problems in physics and astronomy. *Reviews of modern physics*, 15(1):1, 1943.

[2] Alex H Williams, Cian O'Donnell, Terrence J Sejnowski, and Timothy O'Leary. Dendritic trafficking faces physiologically critical speed-precision tradeoffs. *eLife*, 5(DECEMBER2016), 12 2016.

[3] Alexander M Berezhkovskii, Leonardo Dagdug, and Sergey M Bezrukov. Exact solutions for distributions of first-passage, direct-transit, and looping times in symmetric cusp potential barriers and wells. *The Journal of Physical Chemistry B*, 123(17):3786–3796, 2019.

[4] Avi Caspi, Rony Granek, and Michael Elbaum. Enhanced Diffusion in Active Intracellular Transport. *Physical Review Letters*, 85(26):5655–5658, 2000.

[5] Avi Caspi, Rony Granek, and Michael Elbaum. Diffusion and directed motion in cellular transport. *Physical Review E - Statistical Physics, Plasmas, Fluids, and Related Interdisciplinary Topics*, 66(1), 2002.

[6] Benjamin M. Regner, Daniel M. Tartakovsky, and Terrence J. Sejnowski. Identifying transport behavior of single-molecule trajectories. *Biophysical Journal*, 107(10):2345–2351, 2014.

[7] S C Choi and R Wette. Maximum likelihood estimation of the parameters of the gamma distribution and their bias. *Technometrics*, 11(4):683–690, 1969.

[8] Gerald J Hahn and Samuel S Shapiro. Statistical models in engineering. Technical report, 1967.

[9] Kenneth Levenberg. A method for the solution of certain non-linear problems in least squares. *Quarterly of Applied Mathematics*, 2(2):164–168, 1944.

[10] Donald W. Marquardt. An Algorithm for Least-Squares Estimation of Nonlinear Parameters. *Journal of the Society for Industrial and Applied Mathematics*, 11(2):431–441, 1963.

Supplementary table

Table S1: Density of immunogold particles identified by electron microscopy in synapses of axons and dendrites.

**Axons**, *N = 624*		**Dendrites**, *N = 646*		*Neurite, number of synapses sampled*
93		211		*Number of gold particles*
0.149		0.327		*Gold particles / synapse*
30.6		69.4		*Percent of total*
Synaptic	*Extrasynaptic*	*Synaptic*	*Extrasynaptic*	*Subdivision*
26	67	68	143	*Number of gold particles*
0.042	0.107	0.105	0.221	*Gold particles / synapse*
28.0	72.0	32.2	67.8	*Percent of subdivision*
8.6	22.0	22.4	47.0	*Percent of total*

Supplementary figures

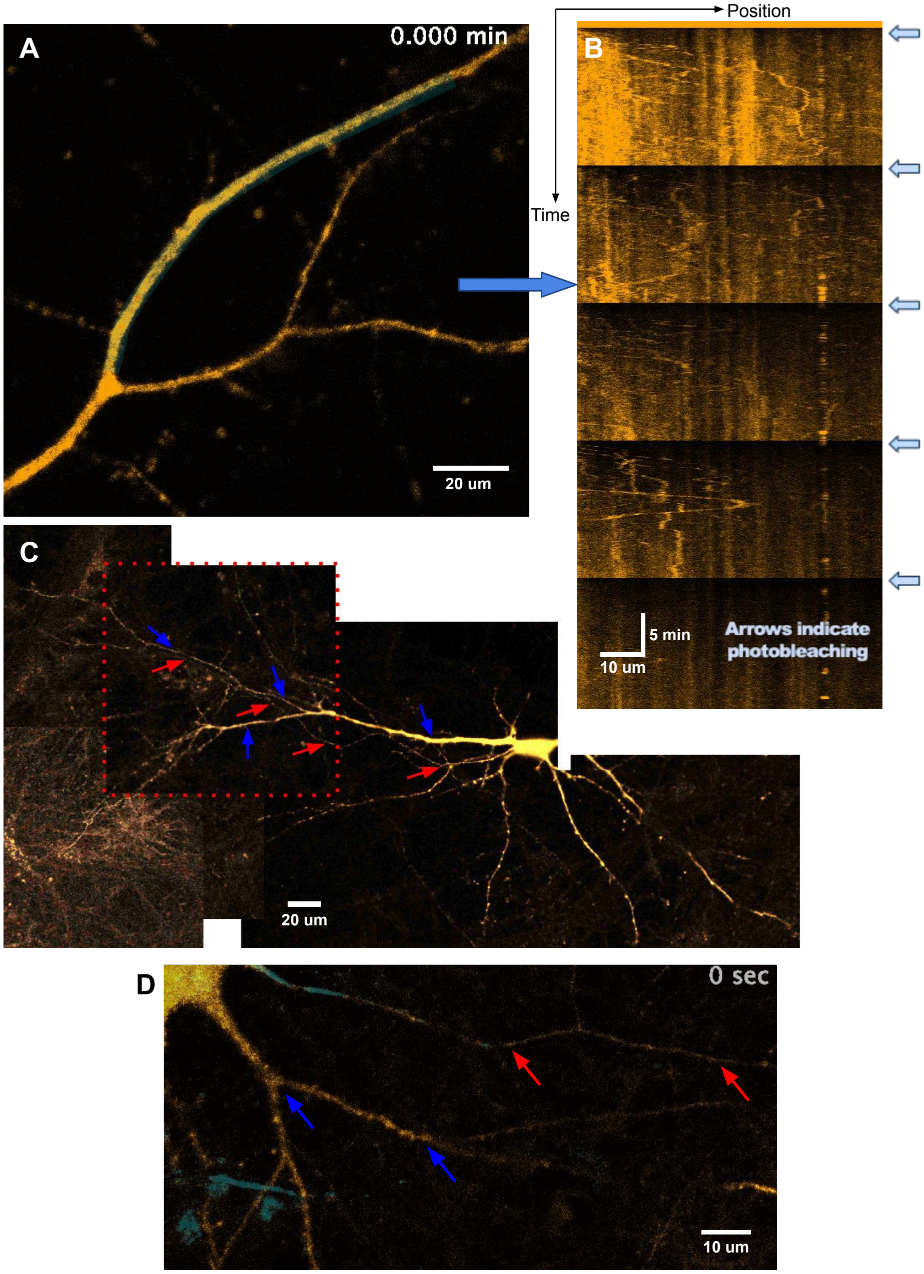

Figure S1: Time series, kymogram generation, and neurite differentiation. (A): A sample time series of Kv4.2-SGFP2 trafficking (orange) depicts a dendrite branch exhibiting frequent transport. The segmented line selection (cyan) surrounding the neurite is used to create kymogram in (B). For animation, see Supplemental Video 1. (B): Kymogram created from segmented line selection in (A). X-axis indicates neurite position and y-axis indicates time. Left-pointing arrows indicate time points for intermittent photobleaching. (C): High-magnification frame for time series (red dotted line) often makes differentiating axons (red arrows) from dendrites (blue arrows) difficult. Low-magnification global images (entire image, stitched) enable neurite differentiation by morphology and branching. (D): Time series depicts characteristic high frequency trafficking in axons (red arrow) compared to dendrites (blue arrow). Anti-GFP-488 (orange) indicates Kv4.2-SGFP2 expression and ankyrin-G (cyan) for axons are used for definitive neurite differentiation. For animation, see Supplemental Video 2.

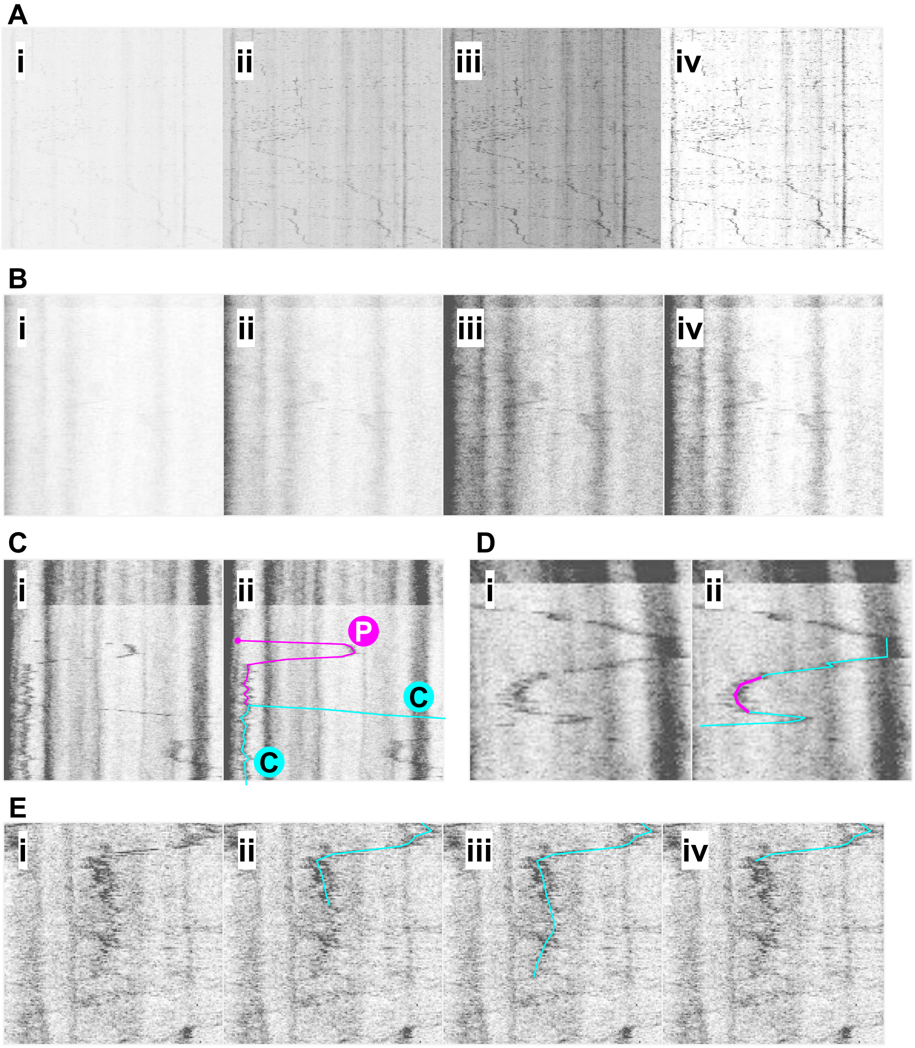

Figure S2: Kymogram contrast enhancement, thresholding, and puncta selection. (A) An example of axonal kymogram processing, where (i) is the raw image, (ii) is following automatic brightness/contrast adjustment, (iii) is following manual brightness/contrast adjustment, and (iv) is following thresholding. (B) A segment of dendritic kymogram is undergoing the same processing as (A). (C) Kymogram parent trajectories P that appear to merge or split into children C trajectories are each recorded as distinct trajectories. In (ii), one P (magenta) splits into two Cs (cyan), for a total of three trajectories. (D) If puncta appear to oscillate and the specific path cannot be resolved (i), trajectories are traced through the center of the oscillations (magenta segment) (ii). (E) To eliminate subjectivity in puncta trajectories that appear or fade away, immobile segments of trajectories are trimmed before and after mobile segments. For the disappearing trajectory shown in (i), both (ii) and (iii) would yield the same trajectory (iv) post trajectory trimming.

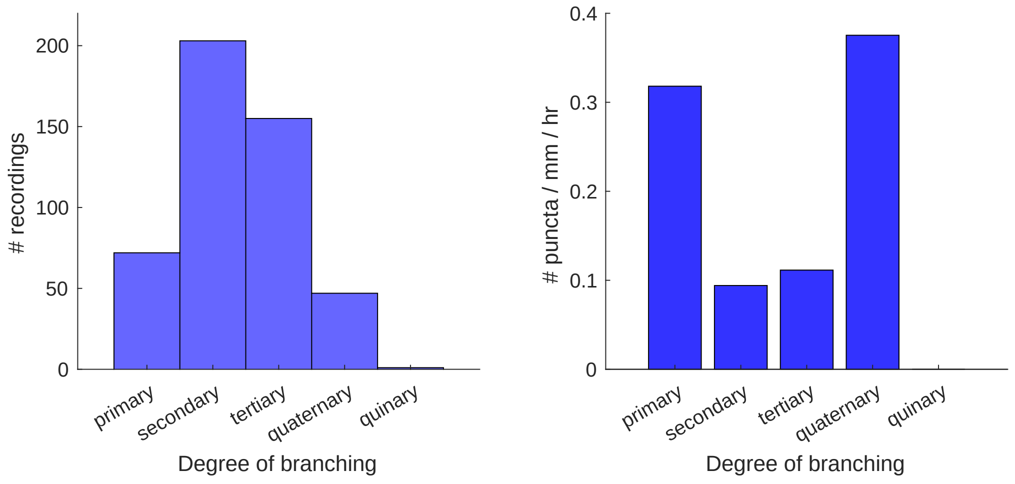

Figure S3: *Left:* Histogram depicting spread of dendritic recordings by degree of branching. Primary indicates the apical dendrite. *Right:* Degree of branching has no strong correlation with puncta frequency.

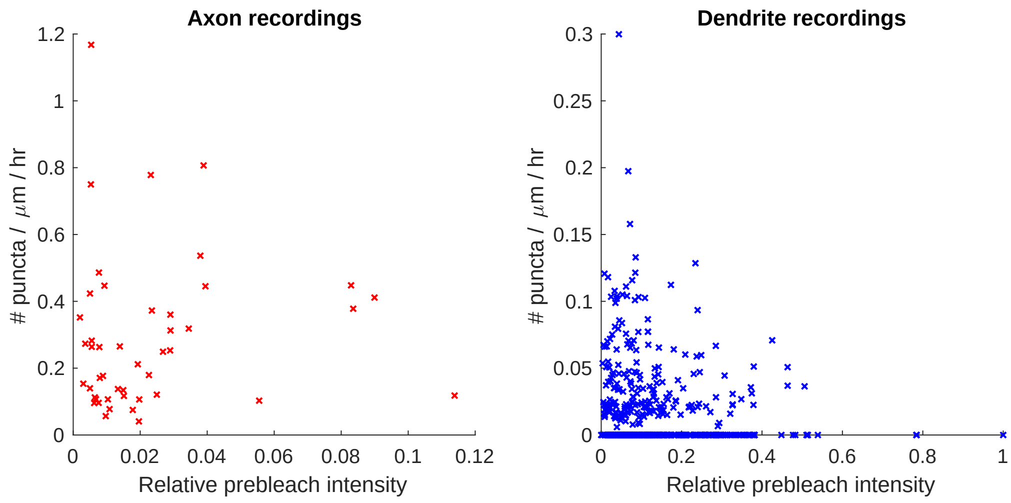

Figure S4: To ensure that puncta appearance or visibility is not an artifact of fluorescence intensity, we plot puncta frequency standardized by neurite length and recording duration versus average prebleach fluorescence intensity for recordings of all axons (*left*) and dendrites (*right*). Neither neurite population shows a strong correlation.

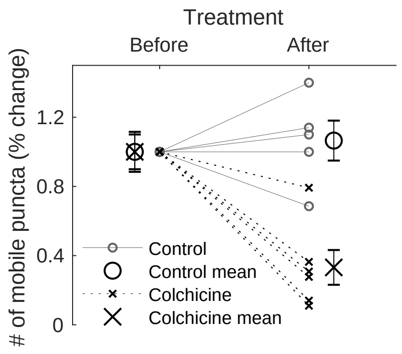

Figure S5: Percent change in number of mobile puncta following administration of microtubule disrupter (colchicine) compared to control (DMSO) during live recording.

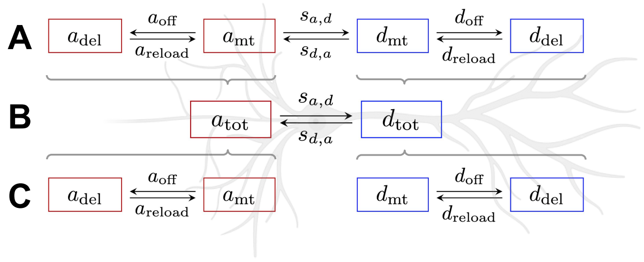

Figure S6: Lumped models of cargo distribution. (A): Mass action model of cargo transit on microtubules (*mt*) and delivery (*del*) in axons (*a*) and dendrites (*d*). (B): Simulation of (A) requires estimation of inter-neurite transit rates $s_{a,d}$ and $s_{d,a}$ using experimental constraints for total (*tot*) cargo. (C): Simulation of (A) requires estimation of cargo offloading (*off*) and reloading (*reload*) rates using experimental constraints for *mt* and *del*.

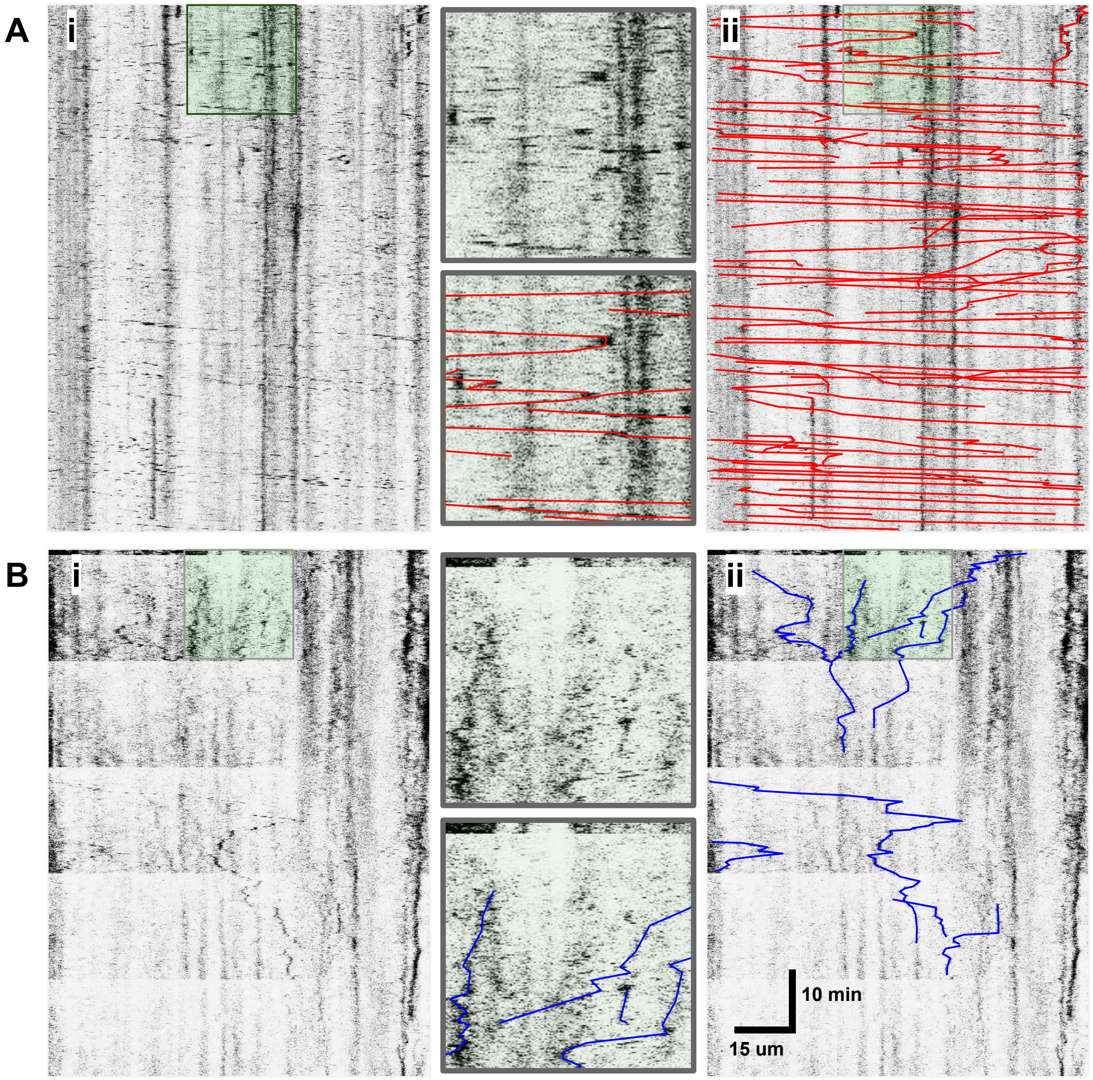

Figure S7: Kv4.2 trafficking is qualitatively different in axons and dendrites (A): Kymograms depicting characteristic axon trajectories (i) with puncta tracing overlaid in (ii). Insets correspond to regions highlighted in green. (B): Same as (A) for characteristic dendrite trajectories.

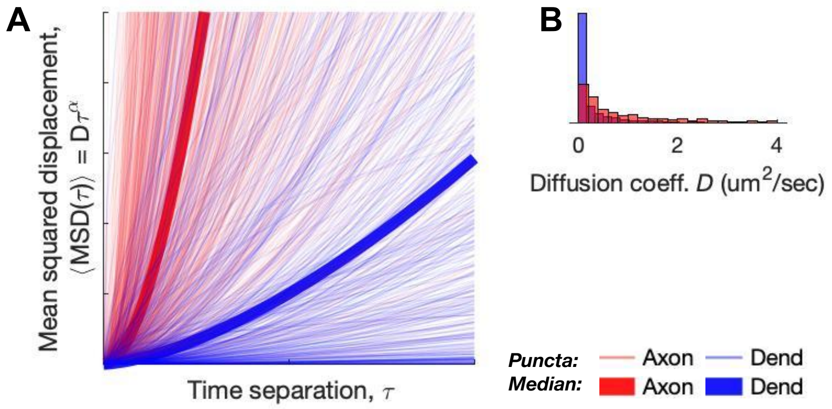

Figure S8: Result of curve fitting for mean squared displacement (MSD) versus time separation (τ), revealing higher degree of superdiffusivity in axons compared to dendrites (A). See details in Methods. Each thin line corresponds to one trajectory fit to $\text{MSD}(\tau) = D\tau^{\alpha}$, where D is the diffusion coefficient, τ is the difference between two time points, and α is the diffusivity coefficient. $\alpha = 1$ is normal diffusion, $\alpha < 1$ is sub-diffusion, and $\alpha > 1$ is superdiffusion. Bold curves indicate the medians of the axonal and dendritic populations. Histogram show distribution for diffusion coefficient D (B).

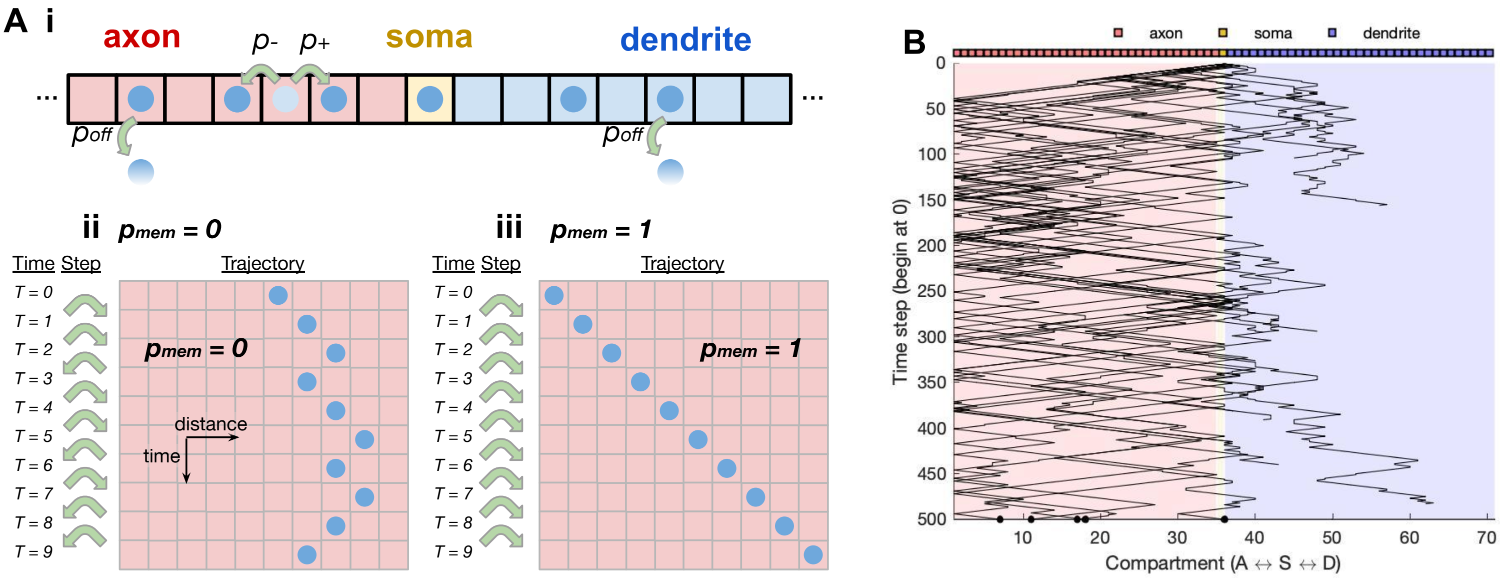

Figure S9: Model of modified random walk. (A): Setup of stochastic simulations along linear multi-compartment model (axon-soma-dendrite), with left/right jump and offloading rates depicted in (i). (ii) and (iii) depict extreme cases for memory parameter p_{mem}. (B): For demonstration of the stochastic model, 10 puncta are simulated over 500 time steps with $p^a_{\mathrm{off}} < p^d_{\mathrm{off}}$ and $p^a_{\mathrm{mem}} > p^d_{\mathrm{mem}}$. The resulting simulated trajectories visually compare to the experimentally-obtained kymograms.
