# Supplementary material for: Paradoxical relationships between active transport and global protein distributions in neurons
Source: Biophys J. 2021 Apr 2;120(11):2085–101. doi: 10.1016/j.bpj.2021.02.048 (PMC8390833; doi:10.1016/j.bpj.2021.02.048)
Supplement: Document S2. Article plus supporting material [file mmc4.pdf]

Biophysical Journal
Article

Biophysical Society

Paradoxical relationships between active transport and global protein distributions in neurons

Adriano Bellotti,[1,3] Jonathan Murphy,[1] Lin Lin,[1] Ronald Petralia,[2] Ya-Xian Wang,[2] Dax Hoffman,[1,*] and Timothy O'Leary[3,*]

[1]National Institute of Child Health and Human Development, Bethesda, Maryland; [2]National Institute on Deafness and Other Communication Disorders, Bethesda, Maryland; and [3]Department of Engineering, University of Cambridge, Cambridge, United Kingdom

ABSTRACT Neural function depends on continual synthesis and targeted trafficking of intracellular components, including ion channel proteins. Many kinds of ion channels are trafficked over long distances to specific cellular compartments. This raises the question of whether cargo is directed with high specificity during transit or whether cargo is distributed widely and sequestered at specific sites. We addressed this question by experimentally measuring transport and expression densities of Kv4.2, a voltage-gated transient potassium channel that exhibits a specific dendritic expression that increases with distance from the soma and little or no functional expression in axons. In over 500 h of quantitative live imaging, we found substantially higher densities of actively transported Kv4.2 subunits in axons as opposed to dendrites. This paradoxical relationship between functional expression and traffic density supports a model—commonly known as the sushi belt model—in which trafficking specificity is relatively low and active sequestration occurs in compartments where cargo is expressed. In further support of this model, we find that kinetics of active transport differs qualitatively between axons and dendrites, with axons exhibiting strong superdiffusivity, whereas dendritic transport resembles a weakly directed random walk, promoting mixing and opportunity for sequestration. Finally, we use our data to constrain a compartmental reaction-diffusion model that can recapitulate the known Kv4.2 density profile. Together, our results show how nontrivial expression patterns can be maintained over long distances with a relatively simple trafficking mechanism and how the hallmarks of a global trafficking mechanism can be revealed in the kinetics and density of cargo.

SIGNIFICANCE Healthy nervous system function depends on continuous replenishment of proteins across complex neuronal morphologies. We provide experimental evidence that an ion channel important for signal integration and regulation of excitability maintains a specific intracellular expression pattern with a relatively simple trafficking mechanism in which cargo is distributed widely and sequestered where needed. This model accounts for substantially different kinetics of cargo movement in different parts of the cell and, paradoxically, predicts that trafficked cargo can be denser in compartments where it is not used. This provides new data on global protein trafficking and insights into the mechanisms underlying expression patterns important for neuronal function. Our results also prompt caution on how to interpret static and dynamic intracellular cargo measurements generally.

INTRODUCTION

Neurons homeostatically maintain function by continually producing proteins and distributing them to function-specific regions of the cell. The logistics of this task are especially challenging in complex neural morphologies with projections that extend hundreds to thousands of microns (1–3). Most proteins have half-lives on the order of hours (4), and some proteins have very precise distributions that are important for cell physiology. Although many proteins, particularly small soluble proteins, are synthesized locally in dendrites (5–7), others are primarily synthesized at or near the soma. Among these are many kinds of ion channels that control neuronal excitability and whose intracellular spatial distribution is tightly regulated (8).

How do intracellular trafficking mechanisms maintain spatial distributions of protein in a complex morphology? This question is critical for our understanding of neuronal biophysics and homeostasis and for unraveling the causes of pathologies associated with dysregulation of protein expression (9,10). Leading conceptual models suggest that cargo is exported and sorted, and local interactions detect and sequester bypassing subunits as needed (11–14). This

Submitted August 5, 2020, and accepted for publication February 22, 2021.

*Correspondence: hoffmand@mail.nih.gov or timothy.oleary@eng.cam.ac.uk

Editor: Anatoly Kolomeisky.

https://doi.org/10.1016/j.bpj.2021.02.048

© 2021 Biophysical Society.

This is an open access article under the CC BY license (http://creativecommons.org/licenses/by/4.0/).

general model, in which cargo is distributed coarsely at a global level and local interactions dictate fine-grained subcellular distribution, is called the sushi belt model (12). However, the extent to which this model explains ion channel distributions in neurons remains open.

In this study, we directly test whether the sushi belt model can account for relationships between ion channel traffic and steady-state distributions of a voltage-gated potassium channel, Kv4.2, whose subcellular distribution is critical for maintaining cellular excitability (8,15,16). Kv4.2 conducts an A-type, transient potassium current, which is abundant in dendrites but scarce in axons (15). Dendritic expression of Kv4.2 is consistent with its hypothesized role in dendritic integration and control of excitability (8,15,16). Functionally, the current density exhibits a five- to sixfold increase along the length of the apical dendrite (16). Localization studies of Kv4.2 corroborate this finding, showing a 70% increase in channel density along the apical dendrite, from soma to the apical region (17).

Axonal Kv4.2-mediated A-currents have not been reported, but other channels that pass A-current have been found in axons (15,18,19). The reported amount of Kv4.2 subunits localized in axons varies substantially among quantitative localization studies. Alfaro-Ruíz et al. report only 1.2% of total CA1 immunogold particles are found in axons (20). Kerti et al. contrastingly report nearly 20%, and the authors remark that "[this result] is surprising, because the Kv4.2 subunit is conceived as a somato-dendritic ion channel" (17). In this study, we recapitulate these results and found predominant expression of Kv4.2 in dendrites with a non-negligible presence in axons. Several studies have measured Kv4.2 trafficking and internalization in dendrites (21–24), but none to date have enabled a quantitative, global model of transport and expression patterns.

We measured and analyzed Kv4.2 active transport in axons and dendrites, including displacement, directional bias, speed, and stall time of individual particles. We inferred parameters in a stochastic model of transport that accounts for cargo dynamics, indicating qualitative differences between axonal and dendritic transport. Axonal transport exhibited superdiffusivity, with long, uninterrupted runs, consistent with lower subunit demand and fewer interactions. By contrast, dendritic cargo trajectories were diffusive, consistent with increased local interactions that interrupt transport.

Surprisingly, we found a greater density of actively transported Kv4.2 subunits in axons than in dendrites. We show that the apparent discordance of high trafficking densities in regions of low functional expression turns out to be consistent with a simple lumped model of intracellular transport derived from the sushi belt model. Furthermore, a spatially discretized version of the model accounts for increasing Kv4.2 localization from proximal to distal compartments. This expression pattern has been extensively characterized and is important for dendritic function (16,25,26), but the question of how it emerges from a relatively simple trafficking mechanism has remained unanswered. We experimentally estimated model parameters including microtubule occupancy and transport rates as functions of distance from soma. Constrained with these data, we provide an analytical solution for the microtubule occupancy profile that can recapitulate the Kv4.2 localization profile along dendrites.

Together, our findings constitute a test of a widely hypothesized, parsimonious model of intracellular transport. We find that this model is consistent with measured and highly specific intracellular protein distributions and predicts observed disparities between transported and delivered cargo.

MATERIALS AND METHODS

Animals and cell culture

All animal procedures are conducted with accordance of the National Institutes of Health Guide for the Care and Use of Laboratory Animals under a protocol approved by the National Institutes of Child Health and Human Development (NICHD)'s Animal Care and Use Committee.

Rat hippocampal dispersed cultures

Hippocampal cultures are prepared from gestational day 18 to 19 wild-type (WT) Sprague-Dawley rats as previously described (23). Briefly, fetal pups are removed from the mother, and hippocampus tissues are dissected and placed in dissection media. For 500 mL of dissection media, we filter sterilized 50 mL 10× Hanks' Balanced Salt Solution (HBSS) (14185-052; Gibco, Gaithersburg, MD), 5 mL penicillin/streptomycin (15140122; Gibco), 5 mL pyruvate (11360070; Gibco), 5 mL HEPES (1 M, 15630080; Gibco), 15 mL of 1 M stock solution glucose (from powder; Sigma-Aldrich, St. Louis, MO), and 420 mL Ultra Pure Water (KD Medical, Columbia, MD).

Tissue was mixed with papain (Worthington Biochemical, Lakewood, NJ) for 45 min at room temperature. Tissues were rinsed for removal of extracellular material with dissection media several times, and dissociated cells were plated in neurobasal media (Thermo Fisher Scientific, Waltham, MA) with 5% fetal bovine serum (HyClone characterized fetal bovine serum, SH30071.03; GE Healthcare LifeSciences, Pittsburgh, PA), 2% GlutaMAX (Thermo Fisher Scientific), and 2% Gibco B-27 supplement (Thermo Fisher Scientific) (subsequently called NB5 media). Cells were incubated in 5% CO_2 at 37°C. After 24 h, cells were transferred to neurobasal media containing 2% GlutaMAX and 2% Gibco B-27 supplement (NB0 media). Half of the media is replaced with fresh NB0 media every 3–4 days, and cells are imaged after 9–13 days in vitro.

Construct

A Kv4.2 construct was conjugated at the N-terminus to strongly enhanced green fluorescent protein (SGFP2) (27), henceforth referred to as Kv4.2-SGFP2. pSGFP2-C1 was a gift from Dorus Gadella (plasmid # 22881; Addgene, Watertown, MA; http://www.addgene.org/22881/; RRID:Addgene_22881). We subcloned mouse Kv4.2 into the SGFP2 plasmid using NheI amd SalI restriction sites.

Transfection

Lipofectamine 2000 transfection was performed following manufacturer protocol with some modifications. 2 μL of Lipofectamine 2000 Transfection Reagent (Thermo Fisher Scientific) and 2 μg of DNA plasmid were each diluted in 200 μL of neurobasal media and incubated at room temperature for 5 min.

The two solutions were then combined and incubated at room temperature for 15–20 min. 100 μL of total mixture was added to each well and incubated at 37°C for 4 h before changing media. The cells were then incubated for an additional minimum of 1 h before imaging.

Immunostaining

After hour-long time series, samples reserved for antibody staining were fixed or permeabilized and immunostained as previously described (28,29) and briefly reiterated here. Upon completion of time series, the coverslips were removed from the imaging chamber, and the location of the neuron of interest was labeled with a fine tip marker. Coverslips were fixed with 4% paraformaldehyde (R 15710; Electron Microscopy Sciences, Hatfield, PA) and 4% sucrose (S9378; Sigma-Aldrich) at room temperature for 15 min, followed by three 1× PBS (14190; Gibco) washes before overnight storage in 1× PBS at 4°C. Coverslips were permeabilized in 0.2% Triton X-100 (T8787; Sigma-Aldrich) for 5 min at room temperature and washed once in 1× PBS for 5 min. Cells were incubated for 1 h at room temperature in 0.04% Triton X-100 solution in 1× PBS containing 1:100 dilution of anti-ankyrin-G rabbit primary antibody (75–146; NeuroMab, Davis, CA) or 1:1000 dilution of MAP2 antibody (Chemicon, Burlington, MA). Upon the completion of primary incubation, coverslips are washed three times with 1× PBS for 5 min. Coverslips are then incubated with secondary antibodies anti-rabbit-555 (1:500) for ankyrin-G or MAP2 and anti-GFP-488 (1:400) (Molecular Probes, Eugene, OR) for 1 h at room temperature before another three washes with 1× PBS. Coverslips were then mounted onto glass slides using ProLong Diamond Antifade Mountant containing DAPI (Invitrogen, Carlsbad, CA).

Microscopy

In this study, we relate experimental observations to microtubule-bound cargo density (*mt*), delivered cargo density (*del*), and total cargo density (*mt* + *del*). These observations are made using various modes of microscopy and are intended as comparative measures of density between axons and dendrites. Election microscopy of synapses reveals *del* cargo. Fluorescence microscopy of transfected Kv4.2-SGFP2 and immunostaining of endogenous Kv4.2 both represent total *mt* + *del* cargo. Time series recordings of mobile puncta reveal *mt* cargo.

Hour-long time series recordings

18-mm coverslips were removed from wells and placed in a Quick Release Chamber (QR-41LP, 64–1944; Warner Instruments, Hamden, CT). Cells were immersed in 800 μL imaging buffer consisting of 1× Tyrode's solution: 135 mM NaCl, 5 mM KCl, 2 mM $CaCl_2$, 1 mM $MgCl_2$, 25 mM HEPES, and 10 mM glucose (all Sigma-Aldrich) at pH 7.4. All imaging was carried out at the NICHD Microscopy and Imaging Core using a Zeiss LSM 710 laser scanning confocal microscope (Carl Zeiss Microscopy, White Plains, NY). Global still images were captured using a 40× oil-immersion objective and stitched together in ImageJ.

Time series were performed using a 63× oil-immersion objective with the pinhole diameter set to 4 airy units. The 495-nm laser line was used for both imaging and bleaching. During imaging, the laser power was set to 4%, and during bleaching, the power was set to 100%. Images were acquired at 1024 × 1024 resolution at 1.0× optical zoom with 750× gain. Time-lapse images were captured at 0.2 Hz for 60–85 min using Zeiss LSM Image Browser software. Z-plane focus was maintained using Zeiss Definite Focus after each frame captured. The cells were temperature- and CO_2-controlled at 37°C and 5% during imaging using a stage top incubator (STXG-WSKMX-SET; Tokai Hit, Fujinomiya, Japan). Every 10–20 min, Kv4.2-SGFP2 was bleached to 30–70% of baseline intensity by the same 495-nm laser (100% power, 10 iterations) over the bleach region of interest.

Extended time series

Coverslips were maintained for extended recordings of up to 11 h. A distilled water reservoir was placed adjacent to the imaging chamber, and the chamber was covered with a 35-mm petri dish to maintain humidity. Time-lapse images were captured at 0.1 Hz with recurrent photobleaching every 50 min. All other procedures and conditions are as previously described.

Colchicine treatment

Samples treated with colchicine (C9754; Sigma-Aldrich) were prepared and time series captured as previously described for hour-long recordings. Six coverslips were treated with colchicine during hour-long recordings. At 30 min into the recording, 1 μL dimethyl sulfoxide (DMSO) (control) or 1 μL DMSO containing 40 μg colchicine was mixed into the imaging chamber, for a final colchicine concentration of 125 μM. The number of mobile puncta per minute was then counted for the duration preceding administration and starting 10 min postadministration.

Electron microscopy

The electron micrographs used in this study were collected for a previous study (30). Mouse hippocampi used for the postembedding immunogold localization were prepared as described previously (31–33). Mice were perfused with phosphate buffer, followed by perfusion with 4% paraformaldehyde + 0.5% glutaraldehyde in phosphate buffer. Fixed brains were vibratomed at 350 μm, then cryoprotected in glycerol overnight and frozen in a Leica EM CPC (Leica Microsystems, Wetzlar, Germany), and processed and embedded in Lowicryl HM-20 resin (Electron Microscopy Sciences) in a Leica AFS freeze-substitution instrument. Thin sections were incubated in 0.1% sodium borohydride + 50 mM glycine in Tris-buffered saline plus 0.1% Triton X-100 (TBST). Then they were immersed in 10% normal goat serum (NGS) in TBST, and primary antibody in 1% NGS/TBST (overnight), then incubated with 10 nm immunogold-conjugated secondary antibodies (Ted Pella, Redding, CA) in 1% NGS in TBST with 0.5% polyethylene glycol (20,000 MW) and stained with uranyl acetate and lead citrate. In this material, the axonal compartment was identified definitively by its synaptic contact. In the original experiments for Sun et al., 2011 (30), a random sample of micrographs were taken from the hippocampus CA1-stratum radiatum from two experiments with 3 + 3 and 2 + 2 WT + knockout (KO) mice; the two experiments produced similar results, and the data were then combined for a total of 646 WT and 642 KO spine profiles. Only the WT spine synapse profiles from the 2011 study were used for this study. The unpublished data from the analysis of the presynaptic terminals are presented here.

Image analysis

Import and kymogram generation

Raw microscope time series were imported into ImageJ with StackReg and Bio-Formats plug-ins. A segmented line selection was drawn through the neurite of interest with the thickness adjusted to cover the diameter of the dendrite (7–12 pixels), with a resultant region of interest as shown in Fig. S1 *A*. Using the plugin KymoResliceWide, a kymogram was generated from the time series in which the horizontal dimension corresponded to the average pixel intensity along the diameter of the cell for each pixel distance from the soma and the vertical dimension was time. A sample segment of kymogram from a bleached neurite is shown in Fig. S1 *B*. The intermittent photobleaching of the region of interest is marked on the sample kymogram with leftward-facing blue arrows.

All kymograms were saved as TIF files, trajectories were saved as TXT files of coordinates, and all were imported into MATLAB (The MathWorks, Natick, MA) for further processing.

Differentiating neurites

Axons and dendrites were differentiated based on their morphology. Dendrites exhibit a steady decrease in diameter with distance from the soma and typically terminate within 1000 μm. Axons extend for thousands of microns and have a relatively constant diameter. The most obvious changes in diameter are in neurite trunks; the axon initial segment is thin like the

axons, at a few microns, whereas dendritic trunks can be several microns thick and broadly blend into the plasma membrane of the soma. In addition, dendrites branch more frequently and at more acute angles, whereas axons can branch at perpendicular or even obtuse angles. Oftentimes, the morphological features differentiating axons and dendrites are not visible in the frame of the time series, and additional global images of neuron must be referenced to distinguish neurites. An example of this is depicted in Fig. S1 *C*, in which the time series frame is outlined in red, but the defining morphological features of the axon and dendrite are only visible in the larger, global image. In Fig. S1 *C*, an axon (*red arrow*) and dendrite (*blue arrow*) exhibiting the aforementioned characteristics are labeled.

Beyond these morphological characteristics, the definitive way to differentiate neurites is with antibody staining for structural proteins exclusively found in one neurite type. Several coverslips were stained with ankyrin-G post-live imaging to confirm identification of the axon initial segment. One such neuron is depicted in Fig. S1 *D* and Video S2.

Contrast enhancement and thresholding

To improve the visibility of puncta trajectories, kymograms were enhanced using automated and manual methods in ImageJ. As an example, raw kymogram sections from a representative axon and dendrite are depicted in Fig. S2*, Ai and Bi.* ImageJ's automatic optimization of brightness and contrast is first performed based on the image's histogram (Fig. S2*, Aii and Bii*). Next, the brightness and contrast settings were manually adjusted by narrowing the visible display range (Fig. S2*, Aiii and Biii*). Lastly, a lower threshold was set, setting pixel values below this threshold to background, as shown in Fig. S2*, Aiv and Biv.*

Puncta trajectory selection

Puncta trajectories were traced using a segmented line selection. The brightness and contrast and threshold settings were adjusted and readjusted for regions of varying immobile fraction within the same kymogram. For instance, a dendrite that is bleached five times over the course of a recording, as in Fig. S1 *B*, required different contrast and threshold settings to visualize puncta in early beaches and later bleaches.

In some cases, puncta appear to merge into one trajectory or split into multiple trajectories. An example of this is depicted in Fig. S2 *C*. In these cases, when tracing trajectories, each parent and child path is designated as an individual trajectory, as in the three trajectories depicted in Fig. S2 *Cii*. The same protocol is followed for two puncta that seemingly merge into one trajectory.

Mobile puncta sometimes rapidly oscillate or vibrate in position. In these cases, if the specific path of the oscillations cannot be resolved, a trajectory was drawn through the mean position of the puncta. An example of this is depicted in Fig. S2 *D*, with a trajectory drawn through the mean position of an oscillation marked in Fig. S2 *Dii*.

Further, a punctum can increase and decrease in fluorescence or appear and disappear during a recording, as shown in Fig. S2 *E*. Because segmented line selections are never drawn through neurite branch points, this likely corresponds to Kv4.2-SGFP2 dispersion or accumulation. To minimize the subjectivity of trajectory selection through such events, each puncta trajectory was trimmed based on a threshold for net displacement, as described in the next section.

Data analysis and modeling

Trajectory trimming

Because only mobile trajectories were considered, puncta with an immobile segment of trajectory before and/or after a mobile segment were trimmed. This was achieved by iterating through each trajectory and summing the net distance traveled. Portions of the trajectories up to the mobility threshold were removed, eliminating stall time before and after mobile segments. The minimal distance threshold was 5 μm for both axon and dendrite trajectories. As an example, both trajectories shown in Fig. S2 *Eii and iii* are interpreted as the same trajectory (Fig. S2 *Eiv*) after trimming. This was useful in cases in which puncta appear or disappear on a kymogram, as in Fig. S2 *Ei*. This also relieves some degree of subjectivity surrounding puncta start and end points and in measurement of stall time.

Analysis of trajectory properties

Net displacement: The net displacement is the absolute distance along the neurite between the puncta's final position and its initial position.

Average speed: Velocity was computed between each consecutive paired frames of a time series. The mean of the absolute values of these instantaneous velocities equals the average speed.

Stall time: Puncta stall time is defined as the fraction of total time during which puncta are traveling with a speed less than 0.1 μm/s.

Mean-square displacement and superdiffusion: In addition to pure random walk motion, puncta may undergo long unidirectional runs. In an ensemble of trajectories, the bulk flow will then be characterized by diffusion with superlinear spread (superdiffusion). The degree of superdiffusivity in individual puncta trajectories was quantified as follows. Mean-square displacement (MSD) was computed by averaging the square of the difference between puncta coordinates some time separation τ apart. This was repeated for τ up to one-quarter the length of the recording duration. MSD was then plotted against τ, and resulting data were fitted to $\mathrm{MSD}(\tau) = D\tau^{\alpha}$ for each individual trajectory to obtain parameters D and α. D and α correspond to diffusion and superdiffusivity coefficients, respectively. In normal diffusion (a linear process), $\alpha = 1$. $\alpha < 1$ corresponds to subdiffusion, and $\alpha > 1$ corresponds to superdiffusion. For the vast majority of trajectories, $\alpha > 1$ (Fig. 3 *Biv*, *first column*). We therefore use the magnitude of α as a measure of the degree of superdiffusivity for individual puncta.

Each trajectory fit to MSD vs. τ is depicted in Fig. S8 *A*, and the bold lines indicate median fits for each neurite type. Puncta in both dendrites and axons undergo motion with similar D (Fig. S8 *B*). However, the MSD tends to increase more rapidly with τ for axonal puncta than for dendritic puncta (Fig. 3 *Biv*). This corresponds to the axonal puncta taking more consecutive steps in the same direction, resulting in motion that is more directed than the memoryless walk of particles in typical diffusion. In other words, axonal puncta exhibited a higher degree of superdiffusivity than dendritic puncta. This discrepancy is consistent with inferred parameters $p^{a}_{\mathrm{mem}} = 0.60$ and $p^{d}_{\mathrm{mem}} = 0.05$ in the stochastic model.

Steady-state analysis for compartmental models

The cargo content of each compartment in a model is defined by a differential equation that sum the quantities of cargo entering and exiting that compartment. A generalized rate $v_{d,r}$ from a donor d to receiver r transfers an amount of mass $dv_{d,r}$. As an example, the system of differential equations for the simplest model in Fig. S6 *B* is as follows:

$$\begin{aligned} \dot{a}_{\mathrm{tot}} &= +s_{d,a}d_{\mathrm{tot}} - s_{a,d}a_{\mathrm{tot}} \\ \dot{d}_{\mathrm{tot}} &= s_{a,d}a_{\mathrm{tot}} - s_{d,a}d_{\mathrm{tot}}. \end{aligned}$$

This system of equations can be solved at steady state to estimate the ratio of these rates. The steady-state assumption sets $\dot{a_{\mathrm{tot}}} = \dot{d_{\mathrm{tot}}} = 0$. Then, rearranging either equation yields

$$\frac{d_{\mathrm{tot}}}{a_{\mathrm{tot}}} = \frac{s_{a,d}}{s_{d,a}}.$$

Bulk flow approximation using drift-diffusion-decay equation to estimate transport parameters

We used population dynamics for average puncta position as a function of distance along the dendrite. Fig. 2 *F* depicts the observed puncta frequency

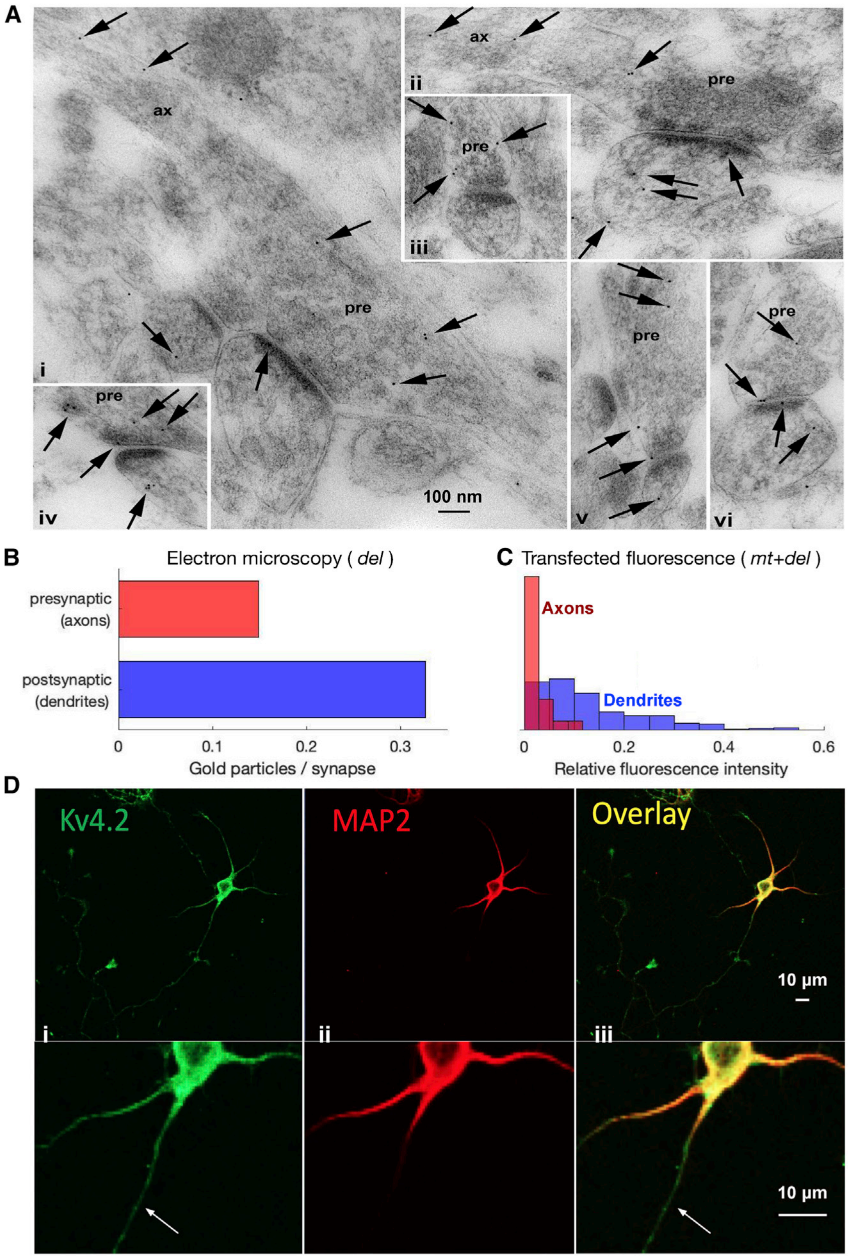

FIGURE 1 Kv4.2 preferentially localizes to dendrites in both endogenous and transfected expression systems, but axonal density is not negligible. (*A*) Immunogold localization (*arrows*) of Kv4.2 in the CA1 stratum radiatum of the hippocampus of WT mice. Synapse profiles show the presynaptic terminal (pre) contacting one or two postsynaptic spines. In (*i*) and (*ii*), the axon (ax) can be traced from the presynaptic terminal. Examples of gold labeling associated with the plasma membrane of the synapse and counted in the accompanying graph include those at the axon synaptic membrane shown in (*iv*), (*v*), and (*vi*); the axon extrasynaptic membrane shown in (*iii*) and (*v*); the dendrite synaptic membrane shown in (*i*) and (*vi*); and the dendrite extrasynaptic membrane shown in (*ii*). (*B*) Quantification of (*A*), in which presynaptic (axonal) and postsynaptic (dendritic) compartments yielded concentrations of 0.149 and 0.327 gold particles per synapse, respectively. Note that the bar "postsynaptic (dendrites)" was published previously in another form in Sun et al., 2011 (30) and is included here for comparison with axons. (*C*) Histogram of the relative prebleach fluorescence intensities of neurons transfected with Kv4.2-SGFP2, showing total subunit count. (*D*) E18 cultured rat hippocampal neurons at DIV5 were immunostained with Kv4.2 antibodies (*i*, *green*) to visualize the endogenous Kv4.2 and MAP2 antibodies (*ii*, *red*) to mark the dendritic arbor. The arrow indicates a representative axon with less Kv4.2 than the surrounding dendrites. To see this figure in color, go online.

of dendritic puncta versus their measured distance from the soma. Puncta trajectories are grouped in 1-μm bins along the dendrites and normalized by the number of dendritic recordings in each bin. To avoid numerical errors with low replicate count, we only considered bins with $\geq$30 dendrite recordings. The resulting distribution of puncta frequency is plotted (Fig. 2 *F*) and displays a trend of decreasing puncta frequency with distance from the soma.

This distribution of puncta frequency versus distance is expected for a collection of mobile particles obeying a drift-diffusion equation with low decay, which we demonstrate analytically. The one-dimensional drift-diffusion equation with decay is as follows:

$$\frac{\partial m(x,t)}{\partial t} = D\frac{\partial^2 m(x,t)}{\partial x^2} + v\frac{\partial m(x,t)}{\partial x} - \left(n_{\mathrm{off}} + w_{\mathrm{mt}}\right)m(x,t), \quad (1)$$

where $m(x, t)$ denotes the concentration of some substance (heat, particles—in this case, Kv4.2-containing puncta) as a function of position x and time t. D is the diffusion coefficient, and v is the mean net velocity (drift). n_{off} is puncta offloading, and w_{mt} is degradation, together modeled as decay. Superdiffusion and unidirectional runs with p_{mem} are not explicitly estimated here but are incorporated into D, as established by Williams et al. (3). p_{mem} is fitted later based on puncta kinetics (Fig. 3).

Because all of our time series were in cells with strong fluorescence many hours after transfection, transport has reached a steady state in which an equal number of puncta enter and leave the recording region. Thus, $\frac{\partial m(x,t)}{\partial t} = 0$, reducing Eq. 1 to

$$D\frac{\partial^2 m(x)}{\partial x^2} + v\frac{\partial m(x)}{\partial x} - \left(n_{\mathrm{off}} + w_{\mathrm{mt}}\right)m(x) = 0. \quad (2)$$

This special case of the drift-diffusion equation is Poisson's equation with decay, which we can solve as a boundary value problem (BVP) using the boundary conditions observed experimentally. From this steady-state distribution, we approximate whether puncta exhibit a forward ($p_+ > p_-$), backward ($p_+ < p_-$), or no ($p_+ \approx p_-$) directional bias. The endpoints of our data,

$$m(0\ \mu\text{m}) = B_P \quad \text{and} \quad m(200\ \mu\text{m}) = B_D, \tag{3}$$

are set for fitting, where B_P and B_D are also the proximal and distal boundaries of the model. Our analytical result is the solution to Eq. 2 with boundary values as in Eq. 3, as follows:

$$m(x) = \Bigg[B_D \exp\left(\frac{1}{2}x\left(\frac{\sqrt{4D(n_{\text{off}}+w_{\text{mt}})+v^2}}{D} - \frac{v}{D}\right) + \frac{100\sqrt{4D(n_{\text{off}}+w_{\text{mt}})+v^2}}{D} + \frac{100v}{D}\right) - B_D \exp\left(\frac{1}{2}x\left(-\frac{\sqrt{4D(n_{\text{off}}+w_{\text{mt}})+v^2}}{D} - \frac{v}{D}\right) + \frac{100\sqrt{4D(n_{\text{off}}+w_{\text{mt}})+v^2}}{D} + \frac{100v}{D}\right) + B_P \exp\left(\frac{1}{2}x\left(-\frac{\sqrt{4D(n_{\text{off}}+w_{\text{mt}})+v^2}}{D} - \frac{v}{D}\right) + \frac{200\sqrt{4D(n_{\text{off}}+w_{\text{mt}})+v^2}}{D}\right) - B_P \exp\left(\frac{1}{2}x\left(\frac{\sqrt{4D(n_{\text{off}}+w_{\text{mt}})+v^2}}{D} - \frac{v}{D}\right)\right)\Bigg] \Big/ \left(\exp\left(\frac{200\sqrt{4D(n_{\text{off}}+w_{\text{mt}})+v^2}}{D}\right) - 1\right). \tag{4}$$

We fitted this analytical solution to the experimental data (Fig. 2 *F*) using least squares to obtain B_P, B_D, D, v, and $(n_{\text{off}} + w_{\text{mt}})$:

$$B_P = 0.49; \quad B_D = 0.11; \quad D = 3.3 \times 10^{-4}; \quad v = 1.0 \times 10^{-5}; \quad n_{\text{off}} + w_{\text{mt}} = 5.4 \times 10^{-8}. \tag{5}$$

This analytical solution is overlaid on the experimental data in Fig. 3 *B*. D and v describe the bulk flow of a population of particles. When Eq. 1 is discretized, D and v characterize the rates of cargo transfer between adjacent compartments:

$$D = \frac{f+b}{2} \quad \text{and} \quad v = b - f, \tag{6}$$

where f and b are the forward and backward rates of the discretized compartmental model. In the limit of large numbers, the propensities of a particle undergoing a random walk p_+ and p_- are related to compartmental model rates f and b according to

$$f = \frac{2p_+ - (p_+ - p_-)^2}{2} \quad \text{and} \quad b = \frac{2p_- - (p_+ - p_-)^2}{2}, \tag{7}$$

as derived in (3). We use the result from the BVP (Eq. 5) along with Eqs. 6 and 7. Because $v \approx 0$ and $n_{\text{off}} + w_{\text{mt}} \approx 0$, we estimate $p_+ = 0.500005$ and $p_- = 0.499995$. Puncta have a minimal directional bias and $p_+ \approx p_-$.

Stochastic model propensities p_{off} and p_{mem}

In the stochastic variant of the model, we simulate individual puncta trajectories as unbiased bidirectional random walks on a one-dimensional lattice with additional propensities p_{off} and p_{mem}. With each time step, individual puncta are removed from the lattice with propensity p_{off}. The left and right jump propensities are therefore $p_- = p_+ = \frac{1-p_{\text{off}}}{2}$ and $p_+ + p_- + p_{\text{off}} = 1$.

p_{mem} is an additional memory feature, depicted in Fig. S9 *Aii and iii*. When $p_{\text{mem}} = 0$, the next directional step is independent of the previous step (Fig. S9 *Aii*). When $p_{\text{mem}} = 1$, the next step is always the same as the previous step (Fig. S8 *Aiii*). A linear interpolation between these extremes, as described in (3), produces a range of memory propensities that scales the average length of the unidirectional run.

Stochastic model fitting

To fit the stochastic model of a random walk modified with offload rate (p_{off}) and memory (p_{mem}) to experimental data, we use a combination of maximal likelihood estimation (MLE) and least-squares fitting. Experiment and model data are normalized and fit to a γ distribution using MLE (MATLAB function fitdist). A γ distribution accommodates all data given its continuity and coverage of a semi-infinite $[0, \infty)$ interval.

The shape and scale parameters of γ fits are compared using nonlinear least-squares data fitting (MATLAB function lsqcurvefit). Generating stochastic model estimates requires a large number of simulated puncta N_s to produce consistent distributions. We employ a moderate $N_s = 10{,}000$ and increase the finite difference step size of lsqcurvefit. A script continuously iterates between 1) running N_s iterations of the stochastic model, 2) MLE of stochastic data, and 3) least-squares fitting of distribution parameters to match those of experimental data. A full description of this method is presented in the Supporting materials and methods.

RESULTS

We first characterized the intracellular distribution of Kv4.2 in our preparation to establish consistency with previously reported results and to validate that the expression of the fluorescent-tagged construct we used for live imaging did not substantially deviate from endogenous expression patterns. Throughout our results, we will refer to two subpopulations of protein subunits: the fraction undergoing active

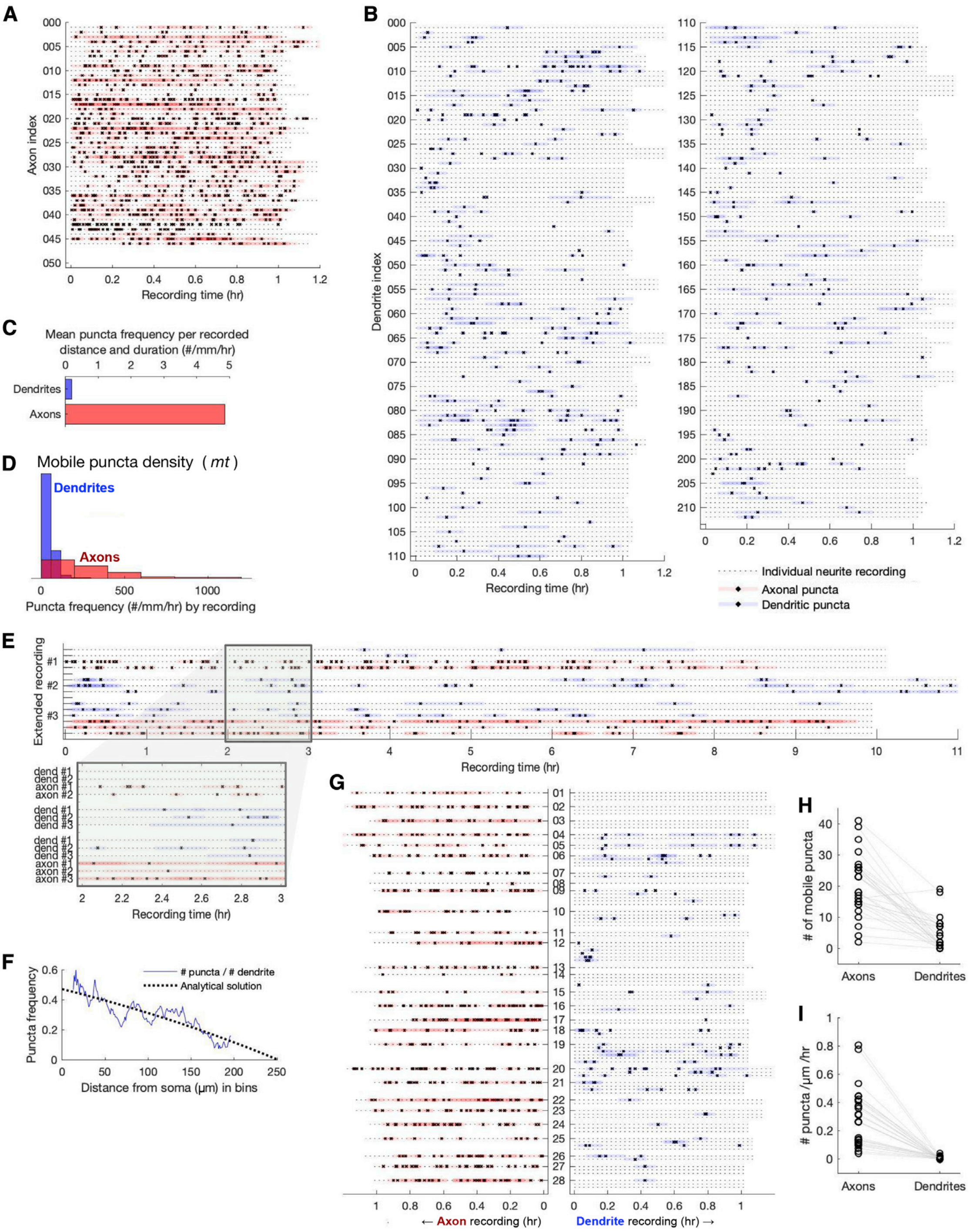

FIGURE 2 Kv4.2 microtubule-based trafficking is observed more frequently in axons than in dendrites. (*A*) Hour-long recordings of 46 axons are depicted, with highlighted sections indicating periods of puncta mobility. (*B*) Hour-long recordings of 213 dendrites are depicted, with highlighted sections indicating periods of puncta mobility. This subset of 478 dendrites has ≥ 1 mobile puncta. (*C*) Puncta frequency (*mt*) in axons and dendrites is standardized by total neurite length visualized and time recorded (units: number of puncta/mm/h). (*D*) Histogram depicting puncta frequency by neurite recording. (*E*) Three extended recordings that substantiate the puncta frequency discrepancy between axons and dendrites over extended periods of observation. (*F*) Puncta frequency (*mt*) decreases with distance from soma in dendrites, consistent with analytical solutions to the drift-diffusion equation. (*G*) Axons and dendrites originating from the same soma (same neuron) are depicted, demonstrating similar trends as those observed in isolated recordings. The central column of numbers indicates an arbitrary recording index for individual neurons. (*H*) Number of mobile puncta per neurite from concurrent recordings in (*G*). (*I*) Number of mobile puncta per neurite standardized by length and time for concurrent recordings from (*G*). To see this figure in color, go online.

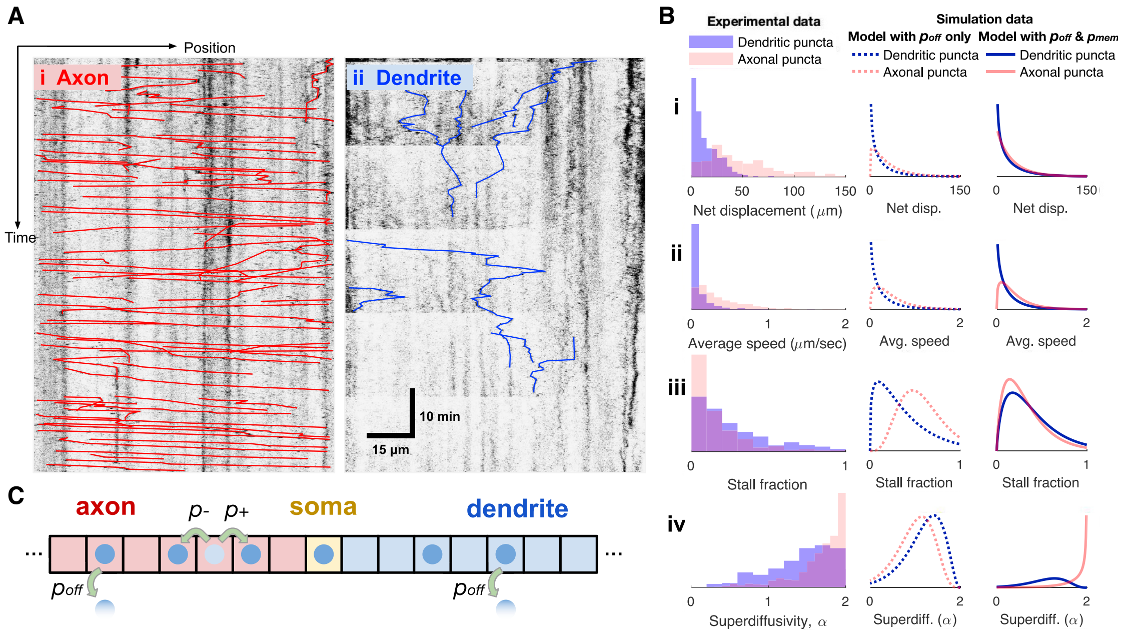

FIGURE 3 Kinetic differences between axons and dendrites are attributable to varying propensities for cargo offloading and unidirectional runs. (*A*) Representative histograms in axon (*i*) and dendrite (*ii*) show typical puncta trajectories and kinetics. Untraced histograms are depicted in Fig. S7. (*B*) Histograms for various transport parameters, normalized as probability density functions. On average, axonal puncta have greater net displacement (*i*), faster speed (*ii*), deceased stall time (*iii*), and increased unidirectional runs (*iv*). Model fits to these results using p_{off} alone (*second column*) as well as using p_{off} and p_{mem} (*third column*) are depicted. (*C*) Setup of stochastic simulations along linear multicompartment model (axon-soma-dendrite), with left and right jump and offloading rates depicted. Complete model is depicted in Fig. S9. To see this figure in color, go online.

transport on microtubules, denoted *mt* (for "microtubule"), and the remaining fraction that is not undergoing active transport, which we denote *del* ("delivered"). It is important to note that the delivered fraction, *del*, comprises the cytosolic pool as well as the functional, membrane-bound fraction. Imaging modalities differ in their ability to distinguish and reliably quantify the *mt* and *del* populations. We therefore use three different comparative measurements: electron microscopy (EM), fluorescent immunostaining, and live imaging.

To quantify endogenous expression, we first used EM after immunogold labeling of endogenous Kv4.2 subunits. Owing to the inherent constraints of EM imaging, we quantified axonal and dendritic expression in identifiable pre- or postsynapses and contiguous extrasynaptic regions, which correspond to *del* cargo densities. We imaged 624 presynaptic and 646 postsynaptic regions. Example micrographs in Fig. 1 *A* show an axon (ax) that can be traced to presynapses (pre).

Sampled immunogold particles identified in the synapses and perisynapses are broadly divided into pre- and postsynaptic regions corresponding to axons and dendrites, respectively. Axons contained 30.6% of all gold particles and 0.15 particles/synapse. Dendrites contained 69.4% of particles and 0.33 particles/synapse (Fig. 1 *B*). This is consistent with previous localization studies (17) in showing substantial, non-negligible subunit localization in axons. Gold particles are also subdivided into synaptic and extrasynaptic regions. In both axons and dendrites, under one-third of particles (28.0 and 32.2%, respectively) of particles were found in synaptic spaces, with the remaining two-thirds in extrasynaptic regions. These percentages and gold particle frequencies are summarized in Table S1.

We next confirmed preferential Kv4.2 expression in dendrites using fluorescence labeling in both endogenous and transfected expression systems. Fig. 1 *D* depicts a neuron with multiple dendrites and one axon stained for somatodendritic marker MAP2 (Fig. 1 *Dii*, *red*). We found substantial Kv4.2 (Fig. 1 *Di*, *green*) in both dendrites and the axon (marked with the *arrow*). The axon exhibits lower fluorescence intensity than the dendrite but is well above levels of background staining. Measurements of Kv4.2-SGFP2 transfected neurons also corroborate this trend, depicted as a histogram of prebleach fluorescence intensity (Fig. 1 *C*). Dendrites contain significantly more Kv4.2 per unit area compared to axons. Taken together, these results establish that the static fraction of Kv4.2 subunits preferentially localizes in dendrites, but its expression in axons is non-negligible, consistent with previous studies (16,17,20).

Kv4.2 microtubule-based trafficking is observed more frequently in axons than in dendrites

We next measured the frequency, density, and kinetic properties of actively transported Kv4.2 subunits on

microtubules (the *mt* population) in both axons and dendrites. To establish reliable estimates, we performed 129 hour-long recordings in neurites of cultured rat hippocampal cells. In total, 507 mobile Kv4.2-SGFP2 puncta were identified among 478 recorded dendrites, and 961 mobile puncta were identified in 46 axons (see Materials and methods). We define mobile puncta as distinct points of fluorescence observed in motion during a recorded time series (Video S1). Ion channels and other integral membrane proteins are transported in membrane vesicles on microtubules (34,35), as depicted in Fig. 4, *Ai–iii*. To measure puncta mobility, we created kymograms from the time series recordings (Fig. S1, *A and B*), traced puncta paths, and filtered for mobile trajectories (see Materials and methods). We assume that axonal and dendritic puncta contain a similar number of Kv4.2 subunits such that our recordings provide a comparative measure of *mt* density between neurite types.

To validate that mobile puncta are transported via active, motor protein-based transport, we applied the microtubule-disrupting drug colchicine (36–38). On average, colchicine administration resulted in a substantial (>60%) decrease in the number of mobile puncta when compared with vehicle (Fig. S5). The Kv4.2-SGFP2 puncta transport that we observe is thus likely to be an active, microtubule-dependent process.

The durations over which puncta are mobile are depicted in Fig. 2, *A* and *B* for axons and dendrites, receptively. Of the 478 dendrites in hour-long recordings, only 213 dendrites (45%) exhibited at least one mobile punctum and are presented in Fig. 2 *B*. Mobile puncta appeared consistently in axons, whereas in dendrites, mobile puncta appear intermittently or not at all. The average length of a sampled region was 85.4 μm in axons compared to 52.3 μm in dendrites. When standardizing these measurements for recording duration and neurite length, the difference in puncta frequency (*mt*) is 4.9 puncta/mm/h in axons vs. 0.18 puncta/mm/h in dendrites, depicted in Fig. 2 *C*. Puncta frequency (*mt*) in dendrites drops to 0.039 puncta/mm/h when considering dendritic recordings with zero mobile puncta (not depicted in Fig. 2 *B*). A histogram showing puncta frequency (*mt*) by neurite recording is depicted in Fig. 2 *D*.

To control for the possibility of global trafficking failure in dendrites that did not show puncta during hour-long recordings, we performed extended recordings lasting 10 h,

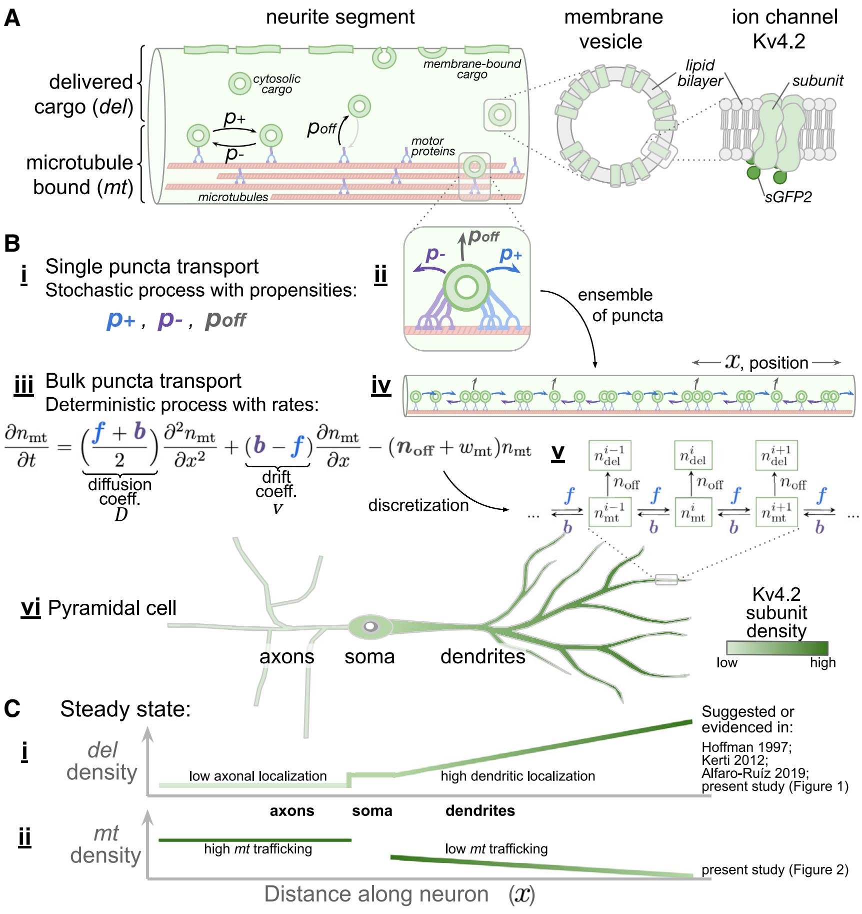

$$\frac{\partial n_{mt}}{\partial t} = \left(\frac{f+b}{2}\right)\frac{\partial^2 n_{mt}}{\partial x^2} + (b-f)\frac{\partial n_{mt}}{\partial x} - (n_{off} + w_{mt})n_{mt}$$

FIGURE 4 Mathematical model of intracellular transport. (*A*) Kv4.2 subunits are divided into microtubule-bound (*mt*) and delivered (*del*) cargo populations. (*B*) Individual vesicles containing cargo (puncta) have microscopic dynamics modeled as a directed random walk (*i* and *ii*). At the population level, the density of cargo (*iv*) behaves as a deterministic process, described by a drift-diffusion equation with decay (*iii*), which can be discretized into a compartmental model (*v*). Compartmental models can represent neurites of a full neuron morphology (*vi*). (*C*) Steady-state cargo densities depend on the relative rates of delivery and transport. Delivered (*del*) Kv4.2 density is low in axons and high in dendrites and increases with dendritic distance (*i*). Microtubule-bound (*mt*) trafficking densities are high in axons and low in dendrites (*ii*). To see this figure in color, go online.

shown in Fig. 2 *E*. The trend in frequency for extended recordings is consistent with that of hour-long recordings, suggesting that hour-long recordings with no puncta are simply a result of sampling.

Puncta frequency (*mt*) was found to be strongly, negatively correlated with distance from soma in dendrites (Fig. 2 *F*). We found no strong correlation between transit frequency and degree of branching from primary (apical) dendrites to quaternary branches (Fig. S3). To ensure that puncta visibility is not an artifact of the fluorescence intensity, we plot puncta frequency (*mt*) versus standardized neurite intensity and find no strong correlation (Fig. S4).

In some cases, it was possible to reliably identify and record from axons and dendrites originating from the same soma to control for cell-to-cell variation in trafficking or metabolism rates. Axons and dendrites from these 28 recordings are depicted alongside each other in Fig. 2 *G*. In all but one case, axons possessed the majority of mobile puncta, even though multiple dendrites were recorded for most neurons. Comparisons of raw puncta count and standardized puncta count (number of puncta/μm/h) are depicted in Fig. 2, *H* and *I*. After standardizing measurements to sampling distance and duration, the axons average a 36-fold increase over the simultaneously recorded dendrites from the same cell.

Taken together, these data establish that actively transported Kv4.2 puncta are present in significantly higher frequencies and densities in axons as compared to dendrites. Thus, the density of trafficked (*mt*) cargo follows the opposite trend of delivered (*del*) subunit density in axons and dendrites.

Kinetics of cargo motion in axons and dendrites reflects differential demand and trafficking mechanisms

The observed disparity between actively transported cargo versus delivered cargo in axons and dendrites raised the question of whether there were differences in the kinetic properties of puncta motion in these compartments. We analyzed Kv4.2 puncta trajectories in axons and dendrites by recording time-lapse images of neurite segments and tracing puncta trajectories on the resulting kymograms (see Materials and methods).

Representative kymograms from axons and dendrites are shown in Fig. 3, *Ai* and *ii*, respectively. Population measurements of puncta kinetics are shown in Fig. 3 *B* (*first column*). On average, axonal puncta have greater net displacement (Fig. 3 *Bi*), faster speed (Fig. 3 *Bii*), deceased stall time (Fig. 3 *Biii*), and increased superdiffusivity (Fig. 3 *Biv*). The computation of these four kinetic measures is detailed in the Materials and methods. Quantifying superdiffusivity involves fitting the MSD of each trajectory to a curve of anomalous diffusion, as discussed in the Materials and methods and depicted in Fig. S8. Taken together, axonal puncta undergo unidirectional runs at high speeds, whereas dendritic puncta appear to change direction more frequently and stall longer. This observation is consistent with low functional expression and a low delivered density of cargo in axons.

We next asked whether a microscopic model of transport could account for observed differences in axonal and dendritic transport and whether these differences might (in part) be explained by differences in cargo demand and sequestration. We expected that higher sequestration rates to the delivered cargo pool (*del*) in dendrites would lead to more interruptions in the directed movement of transported (*mt*) particles, with the opposite trend in axons. We formulated a simple mathematical model of the dynamics of discrete cargo particles as a (directed) random walk (11,39,40).

A cartoon of a neurite segment in Fig. 4 *A* depicts cargo-containing membrane vesicles, corresponding to observed Kv4.2-SGFP2 puncta, undergoing active transport and delivery to a local pool. In keeping with our previous conventions, we assume that cargo belongs either to the microtubule-bound *mt* fraction or the delivered *del* fraction. A microtubule-bound vesicle is attached to opposing motor proteins (Fig. 4 *Bii*), which subject it to stochastic anterograde and retrograde forces (11–14,41–44). We use a stochastic model to represent the net effects of collective forces on individual puncta. In the simplest variant of our model, puncta move in a modified random walk: right ($x = i \rightarrow i + 1$) with propensity p_+ and left ($x = i \rightarrow i - 1$) with propensity p_- per time step Δt. Puncta also detach from the microtubule with net propensity p_{off} per time step Δt.

Puncta trajectories were simulated on a one-dimensional lattice of spatial bins. A schematic of the model is shown in Fig. 3 *A* and contains three types of compartment: axon (A), soma (S), and dendrite (D). Each punctum begins in the S compartment. Puncta in axons and dendrites have distinct offload propensities p^a_{off} and p^d_{off}, consistent with differing cargo demands in each neurite type. We inferred parameters of this model from our experimental measurements of puncta trajectories using maximal likelihood (see Materials and methods and Supporting materials and methods).

The result of fitting for p_{off} is in Fig. 3 *B* (*second column*). Optimal parameter estimates for surface delivery gave $p^a_{\mathrm{off}} < p^d_{\mathrm{off}}$ ($p^a_{\mathrm{off}} = 0.01$ and $p^d_{\mathrm{off}} = 0.04$), consistent with our own observations and published evidence for stronger Kv4.2 sequestration in dendrites versus axons (8,15–17,20,23,45). Thus, a memoryless random walk with differential p_{off} in axons and dendrites is sufficient to explain the differences in net displacement and average speed of cargo (Fig. 3, *Bi* and *ii*). However, stall time distributions and superdiffusivity are not captured fully by this model (Fig. 3, *Biii* and *iv*).

We next incorporated an additional state into the stochastic model with parameter p_{mem} that introduces memory into the kinetics (depicted in Fig. S9 *A* and further explained in Materials and methods). p_{mem} is the probability that a punctum repeats its previous step, giving rise to extended runs if $p_{\mathrm{mem}} > 0$. The result of fitting the model with memory (0 <

$p_{mem} < 1$) is shown in Fig. 3 *B* (*third column*). We again found $p^a_{off} < p^d_{off}$, producing the same trends in displacement and speed (Fig. 3, *Bi* and *ii*). Optimal estimates of the memory term were $p^a_{mem} = 0.60$ and $p^d_{mem} = 0.05$. This is consistent with high superdiffusivity in axons and elevated stall times in dendrites (Fig. 3, *Biii* and *iv*; Fig. S9 *B*), as observed in trajectories (Fig. S6, *A and B*).

Together, this analysis suggests mechanistic differences in the transport of Kv4.2 in axons and dendrites. Increased net displacement, average speed, and puncta frequency in axons are explained by a random walk with minimal delivery (p_{off}) in axons, consistent with Kv4.2 localization to dendrites. However, $p^a_{off} < p^d_{off}$ only partially explains the longer observed runs. Other kinetic parameters—stall time and superdiffusivity—require an additional memory term p_{mem} in our model, suggesting a distinct axonal transport mechanism.

A mathematical model of bulk intracellular transport can account for trafficked and delivered cargo densities

We next asked whether a model of intracellular transport based on the sushi belt model could account for the bulk distributions of actively transported and delivered Kv4.2 cargo. Fig. 4 outlines how this model is derived and how bulk transport relates to the motion of individually measured puncta and delivered cargo (Fig. 4 *A*). The previous results established that the microscopic dynamics of trafficked cargo conform to a random walk. A standard derivation (Fig. 4, *Bi–iv*; Supporting materials and methods) shows that the density of a large population of cargo undergoing such stochastic motion is described by a drift-diffusion equation (3,46):

$$\frac{\partial n_{mt}}{\partial t} = \left(\frac{f+b}{2}\right)\frac{\partial^2 n_{mt}}{\partial x^2} + (b-f)\frac{\partial n_{mt}}{\partial x} - \left(n_{off} + w_{mt}\right)n_{mt}. \tag{8}$$

This partial differential equation relates the position x and time t dependence of microtubule-bound puncta density n_{mt} with rates for bulk flow directed to the right f, left b, and off the microtubule n_{off}. These flow rates relate to diffusion D and drift v coefficients and to stochastic propensities p_+ and p_- (as described in Materials and methods; Eqs. 6 and 7). Microtubule-bound puncta degrade with rate w_{mt}.

For the purpose of simulation and for fitting to data, Eq. 8 can be discretized into a compartmental model in which the flow of cargo obeys the law of mass action (Fig. 4 *Bv*). The spatial scale of compartmentalization can be refined or lumped into smaller or larger compartments, respectively, to account for spatially averaged average or distance-dependent measurements (3,47). We constrained compartmental models of bulk transport to our experimental measurements to test whether global microtubule-based trafficking, combined with differential local sequestration, can account for broad relationships observed in our data and in previous studies. Specifically, we asked the following:

1) Is the disparity between actively transported cargo and delivered cargo in axons and dendrites predicted by the sushi belt model?
2) Can such a model reconcile our trafficking measurements with the known functional dendritic density profile of Kv4.2 reported elsewhere (16,17)?

These questions are schematized in Fig. 4, *Ci* and *ii*, which show the contrasting observed densities of the *del* and *mt* populations, respectively, throughout a neuron.

We first asked whether a lumped compartmental model, constrained by our experimental measurements, can account for measured relationships between trafficked and delivered cargo. Fig. 5 *Ai* depicts a full neuron morphology discretized into spatial compartments. In each compartment, we assumed that cargo is either undergoing transport on microtubules (subscript *mt*) or delivered (subscript *del*) in axonal (*a*) and dendritic (*d*) compartments. As depicted in Fig. 4 *Ai*, *mt* denotes microtubule-bound cargo. Compartments *del* account for all channel subunits that have detached from microtubules, including those in local pools, in the cytosol, and on the plasma membrane. Rates from *mt* to *del* represent cargo

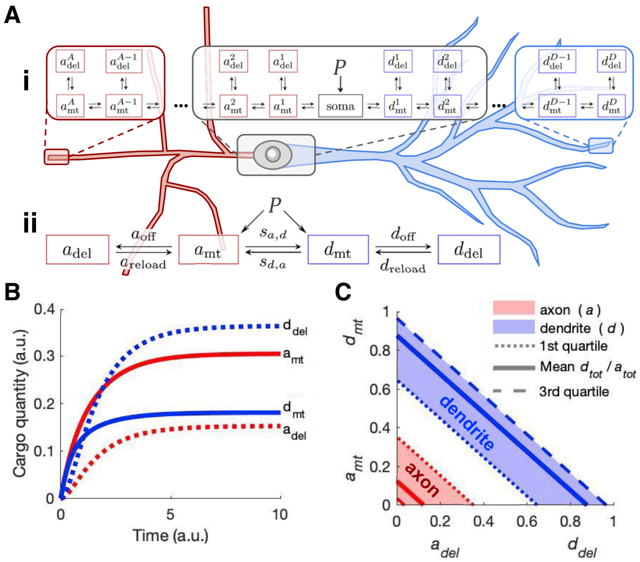

FIGURE 5 Disparity between delivered Kv4.2 (*del*) subunit density and puncta frequency (*mt*) in lumped neurites is explained by a mass-action model. (*A*) Box diagram of mass-action model of axon and dendrite transport. In a full morphology (*i*), the central soma is surrounded by microtubule (*mt*) and delivered (*del*) cargo compartments for axons *a* and dendrites *d*. Arrows denote rates of cargo transfer between compartments. A lumped variant (*ii*) can accommodate experimental constraints to simulate disparities in subunit density between axons and dendrites. (*B*) Result of simulation with experimentally constrained rates, corroborating *mt* and *del* densities observed experimentally. (*C*) Analytical result demonstrating negative correlation between a_{del} and a_{mt} or d_{del} and d_{mt} when restricted to a constant total (*tot*) density. To see this figure in color, go online.

offloading from the microtubules (a_{off}, d_{off}). The reverse rates (a_{reload}, d_{reload}) represent cargo reloading from *del* to *mt*.

Measurements in our study (Figs. 1 and 2) and others (17,20) do not provide axonal data as a function of axonal distance. To incorporate axonal data into a model, we coarsened into a lumped compartmental model that considers only the average density of material in axons and dendrites, irrespective of location (Fig. 5 *Aii*). In the lumped model, $s_{a,d}$ and $s_{d,a}$ represent the net flux of cargo passing between axons and dendrites on microtubules. Allowing separate fluxes, $s_{a,d}$ and $s_{d,a}$, provides for asymmetric flow due to sorting mechanisms that are known to regulate cargo entry into both axons and dendrites (48–50), including mechanisms specific for Kv4.2 (51). All other rates and compartments are as previously described.

The system of differential equations for the lumped compartmental model (Fig. 5 *Aii*) is as follows:

$$\begin{aligned}\dot{a}_{del} &= a_{off}a_{mt} - a_{reload}a_{del} - w_{del}a_{del}\\ \dot{a}_{mt} &= P + a_{reload}a_{del} - a_{off}a_{mt} + s_{d,a}d_{mt} - s_{a,d}a_{mt} - w_{mt}a_{mt}\\ \dot{d}_{mt} &= P + d_{reload}d_{del} - d_{off}d_{mt} + s_{a,d}a_{mt} - s_{d,a}d_{mt} - w_{mt}d_{mt}\\ \dot{d}_{del} &= d_{off}d_{mt} - d_{reload}d_{del} - w_{del}d_{del},\end{aligned} \tag{9}$$

where a generalized rate $v_{d,r}$ describes the flow of mass $dv_{d,r}$ from donor d to receiver r compartments (detailed in Materials and methods). The lumped model does not contain a soma compartment. To account for biosynthesis, we add a fixed production term P to both dendritic and axonal microtubule compartments. Note that flux into both is not assumed to be equal because the flow between axons and dendrites is accounted for by parameters $s_{d,a}$ and $s_{a,d}$. w represents cargo degradation (not depicted in Fig. 5 *Aii*), which, consistent with endolysosomal and authophagic degradation pathways of membrane proteins (52), is faster in *del* than *mt*: $w_{del} > w_{mt}$. The remaining rates in Eq. 9 are estimated from experimental results as described here.

We set $s_{a,d}$ and $s_{d,a}$ to a timescale slower than the other four rates because the distances traveled on microtubules are substantially longer than from *mt* to *del*, especially for large neuron morphologies. To enable a (quasi) steady-state estimate, $s_{a,d}$ and $s_{d,a}$ are set to a timescale 10-fold slower than the other rates, although more modest timescale separation produced the same qualitative result.

We next constrained the rates in this model with our experimental measurements. Rates $s_{a,d}$ and $s_{d,a}$ are estimated using the total (*del* + *mt*) subunit density in axons a_{tot} and dendrites d_{tot}. We grouped *mt* and *del* compartments (from Fig. 5 *Aii*) to produce a model with only a_{tot} and d_{tot}, depicted in Fig. S6 *B*, where

$$a_{tot} = a_{mt} + a_{del} \quad \text{and} \quad d_{tot} = d_{mt} + d_{del}. \tag{10}$$

Fluorescence microscopy of Kv4.2 captures a_{tot} and d_{tot}, in which we found a d_{tot}/a_{tot} ratio of 7.1:1 (see Fig. 1 *C*). Predominant dendritic segregation of the channel is corroborated by other localization studies (8,15,17,20). Steady-state analysis of the differential equations (see Materials and methods) for this model variant (Fig. S6 *B*) yields

$$\frac{d_{tot}}{a_{tot}} = \frac{s_{a,d}}{s_{d,a}} \approx \frac{7.11}{1}. \tag{11}$$

Rates $s_{a,d}$ and $s_{d,a}$ are normalized to axonal measures.

Constraining offload (a_{off}, d_{off}) and reload (a_{reload}, d_{reload}) rates requires estimates of *mt* and *del* cargo in both axons and dendrites. We estimate steady-state *mt* compartments (a_{mt}, d_{mt}) using experimental data for puncta frequency (Fig. 2 *C*). Normalizing to the axon, $d_{mt} = 0.04$ and $a_{mt} = 1$. We estimate steady-state *del* compartments (a_{del}, d_{del}) using our data from EM in synapses (see Fig. 1 *B*; Table S1). Normalizing to the axon, $d_{del} = 2.24$ and $a_{del} = 1$.

To estimate offload and reload rates from *mt* and *del* densities, we modeled axons and dendrites individually, as depicted in Fig. S6 *C*. As twice before, we arrived at expressions that allowed us to solve for ratios of rates:

$$\frac{a_{mt}}{a_{del}} = \frac{a_{reload}}{a_{off}} \approx \frac{1}{1} \quad \text{and} \quad \frac{d_{mt}}{d_{del}} = \frac{d_{reload}}{d_{off}} \approx \frac{0.0374}{2.24}. \tag{12}$$

Together, these estimates (Eqs. 11 and 12) provide constraints for all rates in the lumped model variant (Fig. 5 *Aii*).

The behavior of this model is shown in Fig. 5 *B*. At steady state, the negative correlation between *mt* and *del* compartments in both neurites is clear: $a_{del} < a_{mt}$ and $d_{mt} < d_{del}$. In the context of mass action, the result is intuitive. Because cargo demand in axons is restricted ($a_{del} < d_{del}$), more cargo tends to accumulate in the microtubules of axons versus those of dendrites ($a_{mt} > d_{mt}$).

We next analyzed the negative correlation between *mt* and *del* compartments using Eqs. 10 and 11. We normalized Eq. 11 to a total mass $a_{tot} + d_{tot} = 1$ ($a_{tot} = 0.12$, $d_{tot} = 0.88$) such that each density (a_{mt}, a_{del}, d_{mt}, d_{del}) is a fractional quantity. The resulting steady-state densities of *del* and *mt* cargo are plotted in Fig. 5 *C*. Shaded regions indicate the range of a_{tot} and d_{tot} from first to third quartiles on Fig. 1 *C*. Quantities of cargo *mt* have a clear negative correlation with *del*, and this result holds for any a_{tot} and d_{tot}.

Measured active transport dynamics can account for functional Kv4.2 density along the somatodendritic axis

We next asked whether the transport model described previously could account for the spatial profiles of dendritic distributions of transported and delivered cargo. We measured a decreasing *mt* profile along dendrites with distance from the soma (Fig. 2 *F*). On the other hand, functional and localization studies show that Kv4.2 current and subunit density increase along this axis (16,17).

To examine whether these observations were consistent with the model, we spatial discretized the model (Fig. 5

Ai). Somatic and dendritic compartments are depicted in Fig. 6 *B*, where d^i_{mt} and d^i_{del} represent microtubule-bound and delivered cargo, respectively. We considered a linear dendritic branch extending 250 μm from the soma. To constrain the steady-state concentrations of *mt* compartments (s, d^1_{mt},d^2_{mt},…,d^{10}_{mt}), we used experimental values obtained in Fig. 2 *F*. f_i and b_i denote the forward and backward transport rates along the microtubule. We have combined microtubule offload and reload rates into a net rate d^i_{off} for each d^i_{mt}.

We next computed directional bias in punctal velocity as a function of distance to constrain rates f_i and b_i. We averaged the instantaneous velocities of each puncta trajectory in bins by distance from the soma. Mean puncta velocity showed an increasing linear trend with p-value < 0.01, as plotted in Fig. 6 *A* with 90% confidence intervals. With a positive y intercept and slope, the mean punctal velocity is directed distally and increases with distance from the soma. That is, $f_i > b_i$ and $f_{i+1} >> b_{i+1}$. The velocities in Fig. 6 *A* range from 1.5 to 5.2 μm/s and are scaled according to the spatial discretization of the model to estimate f_i-values and b_i-values. The diffusion coefficient $D = \frac{f_i + b_i}{2}$ was estimated using Eq. 6, and D remains constant throughout the dendritic tree.

We constrained the cargo offloading rate d^i_{off} in each spatial compartment by solving the corresponding equations at steady state (Fig. 6 *B*). We found that a profile of increasing f_i-values and decreasing b_i-values with distance from the soma produces an increasing profile of d^i_{off}-values. In other words, for cargo with an increasing directional bias such that $0 < f_i - b_i < f_{i+1} - b_{i+1}$ and decreasing *mt* profile, mass action dictates increasing offload rates $d^i_{off} < d^{i+1}_{off}$ with distance from the soma.

Increasing d^i_{off}-values can produce *del* profiles that have the opposite spatial profile as *mt* densities. To demonstrate this, we simulate regulated Kv4.2 production, distribution, and delivery in our model. In the soma, Kv4.2 biosynthesis P is regulated by active subunits in *del* compartments, as depicted in Fig. 6 *B*. The equation for negative feedback is

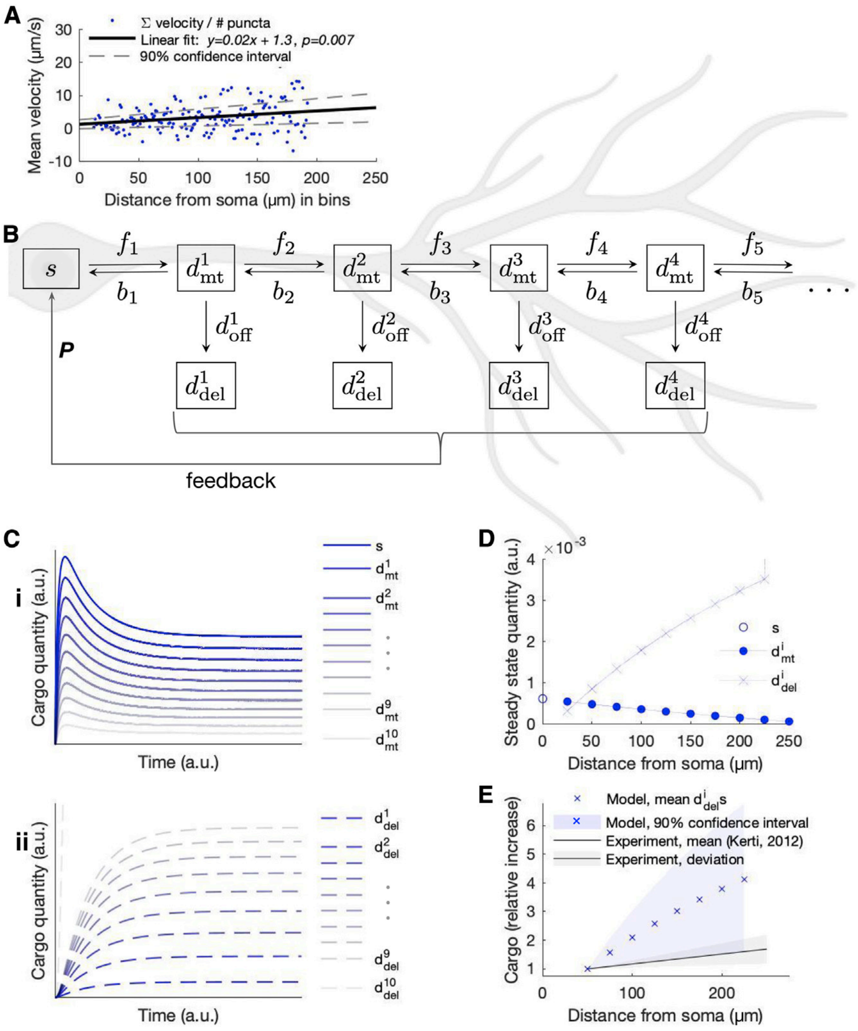

FIGURE 6 Opposing gradients in *del* and *mt* along somatodendritic axis are reconciled with mass-action kinetics. (*A*) The mean instantaneous velocities for all dendritic puncta are standardized by puncta frequency along the length of the dendrite. A linear tread line is plotted through the data with 90% confidence intervals, indicating a positive (distal) velocity bias that increases with distance from soma. (*B*) Box diagram of a mass-action model of dendritic transport and delivery with feedback. The dendrite is spatially discretized, with each discretization i comprising a microtubule-bound d^i_{mt} and delivered d^i_{del} compartment. f_i-values, b_i-values, and d^i_{off}-values denote rates between compartments. Degradation rates for all compartments are simulated but not depicted. (*C*) Simulation results for d^i_{mt} (*i*) and d^i_{del} (*ii*). (*D*) Steady-state concentrations of all compartments. (*E*) Steady-state concentrations of d^i_{del}-values standardized by d^2_{del} at 50 μm overlaid on equivalently standardized experimental data of Kv4.2 localization (17). To see this figure in color, go online.

$$P = K_P\left(d_{\text{avg,del}}^{\text{target}} - \frac{\sum_{i=1}^{10} d_{\text{del}}^i}{10}\right),$$

where $d_{\text{avg,del}}^{\text{target}}$ is the target *del* concentration (setpoint), $\frac{\sum_{i=1}^{10} d_{\text{del}}^i}{10}$ is mean delivered cargo (process variable), and K_P is the proportional gain. This control loop feedback mechanism is consistent with experimental observations that Kv4.2 expression is regulated as a function of neuron excitability (22–24,53). However, the exact nature of the feedback signal is unknown. We therefore used the averaged delivery rate over all d_{del}^i, which amounts to simple proteostasis and is a realistic feedback signal in a neuron (54).

The result of simulating this model is depicted in Fig. 6 *C*. d_{mt}^i-values assume a profile similar to that observed experimentally (Fig. 2 *F*), with decreasing density with dendritic distance (Fig. 6 *Ci*). d_{del}^i-values form the opposite profile—increasing density with dendritic distance (Fig. 6 *Cii*). Steady-state densities versus position along the dendrite are plotted in Fig. 6 *D*.

The increasing d_{del}^i density is notable because localization experiments (17) and, to a larger degree, recordings of A-type current (16) both demonstrate increasing profiles with distance from the soma. In this analysis, the gradient of steady-state d_{del}^i-values (Fig. 6 *D*) largely depends on that of the mean velocities (Fig. 6 *A*) used to constrain the directional bias $f_i > b_i$. In Fig. 6 *E*, we plot d_{del}^i-values for the linear fit and 90% confidence intervals from our measured directional bias. On the same plot, we shade the reported localization profile of Kv4.2 immunogold-tagged particles from Kerti et al.'s 2012 (17) study. Our model predicts an asymmetric profile of d_{del}^i-values that falls within a standard deviation of localization data. Together, these results provide an account of how a previously unexplained and highly organized protein expression pattern can emerge from relatively simple transport mechanisms.

A summary of the relationships between *mt* and *del* cargo densities

The questions we address in this study concern how densities of actively trafficked cargo (*mt*) relate to delivered localization (*del*). We lastly provide a summary of these relationships. A cartoon depicting *mt* and *del* is shown in Fig. 7, where *mt* and *del* densities are depicted by the shading and outline of the cell, respectively.

We depict the negative correlation between *mt* and *del* in lumped neurites in Fig. 7 *A*. As *mt* density in a neurite increases, we expect a corresponding decrease in *del* and vice versa. In our measurements, the intracellular distribution of Kv4.2 most closely resembles the rightmost cartoon (Fig. 7 *A*), as labeled. We next depict the negative correlation between *mt* and *del* along a gradient within a single neurite in Fig. 7 *B*. We find that the *mt* density of Kv4.2 decreases with dendritic distance, which is sufficient to explain the well-established *del* profile. An intuitive explanation for these negative relationships (Fig. 7, *A* and *B*) correlates cargo demand in *del* to local interactions that sequester and deplete passing cargo in *mt*.

Lastly, the kinetic properties of trafficked cargo in *mt* partially reflect the cargo demand in *del* (Fig. 7 *C*). Transport in axons is mechanistically distinct such that cargo is trafficked efficiently through regions of low, sparse demand with direct, unidirectional trajectories. Increased cargo demand in dendrites results in diffusive, winding, and meandering trajectories.

DISCUSSION

In this study, we measured densities of delivered Kv4.2 in synapses (*del*). We also analyzed mobile subunits (*mt*) in bleached neurites to estimate basal trafficking frequencies. We found substantially higher Kv4.2 subunit trafficking on *mt* in axons than in dendrites (Fig. 2). The *mt* distribution does not match established Kv4.2 functional or localization profiles (*del*, Fig. 1). However, just as a satellite photo of car traffic might reveal the highest density of cars on freeways as opposed to parked at a destination, our analysis showed that our measurements are consistent with a mass-action model of transport (Fig. 5). This implies that increased dendritic demand and local interactions with mobile cargo depletes dendritic microtubule-bound subunit density (*mt*). In axons, low subunit demand can result in higher trafficking density (*mt*). Indeed, previous localization studies, as well as our own observations, reveal a non-negligible density of axonal Kv4.2 (17,20). With no known presynaptic function, this axonal fraction might be an artifact of mass action, as our study suggests. We stress that although this paradoxical regime is consistent with standard trafficking models, the inverse relationship may not hold in all situations. Trivially, if transported cargo is strongly filtered from a compartment where it is not expressed, then trafficked density and expressed density may not show inverse relationships.

We also observed proximal-to-distal trends in dendritic Kv4.2 expression, particularly in puncta frequency (*mt*) and directional bias (Fig. 6). When these parameters constrain the rates of a mass-action model, the resultant delivered subunit density (*del*) can account for its well-established, characteristic functional profile (16,17). A similar increasing profile also exists for hyperpolarization-activated cyclic nucleotide-gated channels (55). Moreover, a study of hyperpolarization-activated cyclic nucleotide-gated channel trafficking and surface expression reveals similar dendritic trafficking dynamics to those reported here but no measured kinetic trend with distance along the dendrite (56). We suspect that the distance-dependent trafficking parameters observed here are partial contributors to the functional expression profiles of Kv4.2 channels, which likely rely

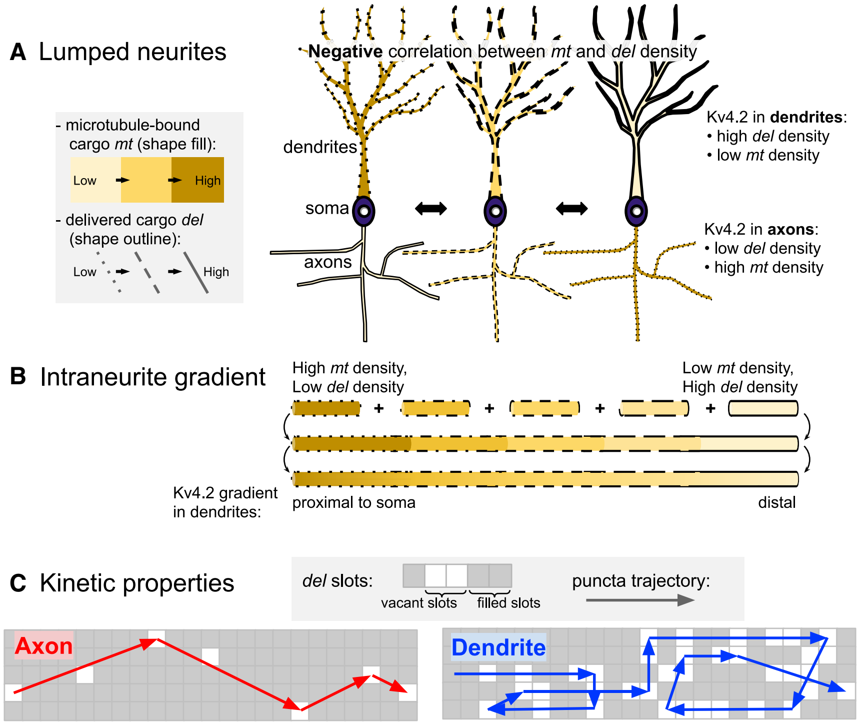

FIGURE 7 A summary of the relationships between microtubule-bound (*mt*) and delivered (*del*) cargo densities. The negative correlation between *mt* and *del* holds in lumped neurites (*A*) and along intraneurite gradients (*B*). (*C*) The kinetic properties of individual puncta trajectories in *mt* also reflects cargo demand in *del*. To see this figure in color, go online.

heavily on local interactions with membrane protein complexes, in line with the sushi belt model. Other mechanisms for supporting distal dendritic expression include elegant passive mechanisms that exploit differences between volume and surface-confined diffusion (57).

Transport kinetics of Kv4.2 puncta differed quantitatively in axons and in dendrites (Fig. 3). Puncta in axons showed increased superdiffusivity, with increased net displacement, increased velocity, and decreased stall time. The opposite is observed in dendrites. This relationship makes sense physiologically; there is greater sequestration (higher p_{off}) in dendrites, presumably because of local interactions with Kv4.2 cargo. However, increased microtubule offloading only partially explains the observed differences in kinetics. A random walk with memory better characterizes the observed stall fraction and diffusivity. We therefore infer that transport in axons is mechanistically different, with microtubule configuration or motor composition increasing the likelihood of unidirectional runs.

A number of implicit assumptions are made in our modeling. Notably, microtubule orientation is not considered in mass-action or stochastic simulations. Axons have a uniform arrangement of “plus-end-out” microtubules, whereas dendritic orientation is mixed. However, the microtubule motors are also mixed, with both kinesins and dyneins present in all neurites. Our understanding of Kv4.2 interaction with microtubule motors is incomplete, with only Kif17 identified as having a role in subunit trafficking (58). Without a comprehensive understanding of all motors and localization mechanisms, we assume the molecular “tug of war” between motors is equivalent in dendrites and axons, with no bias for microtubule orientation.

The models used in this study are an approximation of the molecular mechanisms known to underly trafficking. We briefly review the transport and expression mechanisms of Kv4.2 lumped within our models. Kv4.2 interacts with kinesin Kif17, suggesting transport on microtubules. In the absence of Kif17, Kv4.2 fails to localize in dendrites (58). Deletion of a portion of the C-terminus or fusion with myosin Va restricts expression of Kv4.2 to the somatodendritic region (51,59,60). Further, Kv channel-interacting proteins (KChIPs) have been established as auxiliary subunits that promote Kv4.2 exit from the endoplasmic reticulum for surface expression (4,22,61). An auxiliary subunit, DPP6, attached to Kv4.2 by a transmembrane domain (62), assists in trafficking Kv4.2 out of the endoplasmic reticulum to the plasma membrane (63). Dumenieu et al. (64) summarize these results with the following working hypothesis: Kv4.2 is trafficked short distances such as to proximal dendrites or within spines on actin filaments via myosin Va, whereas long-range transport is mediated along microtubules via KChIPs and Kif17.

There are unavoidable methodological tradeoffs between attempting to quantify protein at physiologically low expression levels and inducing high expression that enables live imaging. We assumed that the transport behavior of the transfected construct Kv4.2-SGFP2 is similar to that of endogenously expressed subunits. Our results are therefore subject to this caveat. It is possible that transfection of a recombinant construct alters intracellular expression profiles.

For this reason, we validated expression profiles by labeling and quantifying both endogenous and transfected Kv4.2 subunits while using a construct that has been thoroughly compared to endogenous channel (28). We anticipate that our approach can spur future work that will mitigate experimental challenges by designing enhanced fluorescent probes that might be suited to live superresolution imaging. Such methods will be crucial for peering deeper into the logic of intracellular protein regulation.

SUPPORTING MATERIAL

Supporting material can be found online at https://doi.org/10.1016/j.bpj.2021.02.048.

AUTHOR CONTRIBUTIONS

A.B. and T.O. designed the study. A.B., L.L., R.P., and Y.-X.W. carried out all experiments. A.B. performed numerical simulations. J.M. contributed the construct and experiment support. A.B., J.M., D.H., and T.O. designed experiments. A.B., J.M., L.L., R.P., and T.O. analyzed results. A.B. and T.O. wrote the manuscript. All authors discussed the results and commented on versions of the manuscript.

ACKNOWLEDGMENTS

We thank Alexander M. Berezhkovskii (Mathematical and Statistical Computing Laboratory, Office of Intramural Research, Center for Information Technology, NIH) for his help with modeling of the modified random walk. We thank Dr. Vincent Schram and Lynne Holtzclaw for their assistance with microscopy performed at the NICHD Microscopy & Imaging Core. We also thank members of the Hoffman lab and the Univ of Cambridge control group for advice and suggestions on this work. A.B. is supported by the National Institutes of Health, the NIH Oxford-Cambridge Scholars Program, the Gates Cambridge Scholarship Programme, and the UNC School of Medicine MD-PhD Program. A.B. and D.H. are funded and supported by the *Eunice Kennedy Shriver* National Institute of Child Health and Human Development intramural research program. R.P. and Y.-X.W. were supported by the Intramural Research Program of NIH/National Institute on Deafness and Other Communication Disorders (Advanced Imaging Core-ZIC DC000081). T.O.L. is supported by ERC grant StG 716643 FLEXNEURO.

REFERENCES

1. Kapitein, L. C., and C. C. Hoogenraad. 2011. Which way to go? Cytoskeletal organization and polarized transport in neurons. *Mol. Cell. Neurosci.* 46:9–20.
2. Maday, S., A. E. Twelvetrees, …, E. L. Holzbaur. 2014. Axonal transport: cargo-specific mechanisms of motility and regulation. *Neuron.* 84:292–309.
3. Williams, A. H., C. O'Donnell, …, T. O'Leary. 2016. Dendritic trafficking faces physiologically critical speed-precision tradeoffs. *eLife.* 5:e20556.
4. Shibata, R., H. Misonou, …, J. S. Trimmer. 2003. A fundamental role for KChIPs in determining the molecular properties and trafficking of Kv4.2 potassium channels. *J. Biol. Chem.* 278:36445–36454.
5. Holt, C. E., and E. M. Schuman. 2013. The central dogma decentralized: new perspectives on RNA function and local translation in neurons. *Neuron.* 80:648–657.
6. Glock, C., M. Heumüller, and E. M. Schuman. 2017. mRNA transport & local translation in neurons. *Curr. Opin. Neurobiol.* 45:169–177.
7. Fonkeu, Y., N. Kraynyukova, …, T. Tchumatchenko. 2019. How mRNA localization and protein synthesis sites influence dendritic protein distribution and dynamics. *Neuron.* 103:1109–1122.e7.
8. Stuart, G., N. Spruston, and M. Häusser. 2016. Dendrites. Oxford University Press, Oxford, UK.
9. Olkkonen, V. M., and E. Ikonen. 2006. When intracellular logistics fails–genetic defects in membrane trafficking. *J. Cell Sci.* 119:5031–5045.
10. Le Quesne, J. P. C., K. A. Spriggs, …, A. E. Willis. 2010. Dysregulation of protein synthesis and disease. *J. Pathol.* 220:140–151.
11. Newby, J., and P. C. Bressloff. 2010. Local synaptic signaling enhances the stochastic transport of motor-driven cargo in neurons. *Phys. Biol.* 7:036004.
12. Doyle, M., and M. A. Kiebler. 2011. Mechanisms of dendritic mRNA transport and its role in synaptic tagging. *EMBO J.* 30:3540–3552.
13. Buxbaum, A. R., G. Haimovich, and R. H. Singer. 2015. In the right place at the right time: visualizing and understanding mRNA localization. *Nat. Rev. Mol. Cell Biol.* 16:95–109.
14. Buxbaum, A. R., Y. J. Yoon, …, H. Y. Park. 2015. Single-molecule insights into mRNA dynamics in neurons. *Trends Cell Biol.* 25:468–475.
15. Sheng, M., M. L. Tsaur, …, L. Y. Jan. 1992. Subcellular segregation of two A-type K+ channel proteins in rat central neurons. *Neuron.* 9:271–284.
16. Hoffman, D. A., J. C. Magee, …, D. Johnston. 1997. K+ channel regulation of signal propagation in dendrites of hippocampal pyramidal neurons. *Nature.* 387:869–875.
17. Kerti, K., A. Lorincz, and Z. Nusser. 2012. Unique somato-dendritic distribution pattern of Kv4.2 channels on hippocampal CA1 pyramidal cells. *Eur. J. Neurosci.* 35:66–75.
18. Colbert, C. M., and E. Pan. 2002. Ion channel properties underlying axonal action potential initiation in pyramidal neurons. *Nat. Neurosci.* 5:533–538.
19. Trimmer, J. S. 2015. Subcellular localization of K+ channels in mammalian brain neurons: remarkable precision in the midst of extraordinary complexity. *Neuron.* 85:238–256.
20. Alfaro-Ruíz, R., C. Aguado, …, R. Luján. 2019. Expression, cellular and subcellular localisation of Kv4.2 and Kv4.3 channels in the rodent Hippocampus. *Int. J. Mol. Sci.* 20:246.
21. Varga, A. W., A. E. Anderson, …, J. D. Sweatt. 2000. Input-specific immunolocalization of differentially phosphorylated Kv4.2 in the mouse brain. *Learn. Mem.* 7:321–332.
22. Varga, A. W., L.-L. Yuan, …, J. D. Sweatt. 2004. Calcium-calmodulin-dependent kinase II modulates Kv4.2 channel expression and upregulates neuronal A-type potassium currents. *J. Neurosci.* 24:3643–3654.
23. Kim, J., S. C. Jung, …, D. A. Hoffman. 2007. Regulation of dendritic excitability by activity-dependent trafficking of the A-type K+ channel subunit Kv4.2 in hippocampal neurons. *Neuron.* 54:933–947.
24. Hammond, R. S., L. Lin, …, D. A. Hoffman. 2008. Protein kinase a mediates activity-dependent Kv4.2 channel trafficking. *J. Neurosci.* 28:7513–7519.
25. Hoffman, D. A., and D. Johnston. 1998. Downregulation of transient K+ channels in dendrites of hippocampal CA1 pyramidal neurons by activation of PKA and PKC. *J. Neurosci.* 18:3521–3528.
26. Magee, J. C., and M. Carruth. 1999. Dendritic voltage-gated ion channels regulate the action potential firing mode of hippocampal CA1 pyramidal neurons. *J. Neurophysiol.* 82:1895–1901.
27. Kremers, G. J., J. Goedhart, …, T. W. Gadella, Jr. 2007. Improved green and blue fluorescent proteins for expression in bacteria and mammalian cells. *Biochemistry.* 46:3775–3783.
28. Kim, J., D. S. Wei, and D. A. Hoffman. 2005. Kv4 potassium channel subunits control action potential repolarization and frequency-dependent broadening in rat hippocampal CA1 pyramidal neurones. *J. Physiol.* 569:41–57.

29. Lin, L., W. Sun, …, D. A. Hoffman. 2013. DPP6 regulation of dendritic morphogenesis impacts hippocampal synaptic development. *Nat. Commun.* 4:2270.

30. Sun, W., J. K. Maffie, …, D. A. Hoffman. 2011. DPP6 establishes the A-type K(+) current gradient critical for the regulation of dendritic excitability in CA1 hippocampal neurons. *Neuron.* 71:1102–1115.

31. Petralia, R. S., Y. X. Wang, …, R. J. Wenthold. 2010. Organization of NMDA receptors at extrasynaptic locations. *Neuroscience.* 167:68–87.

32. Lin, L., J. G. Murphy, …, D. A. Hoffman. 2018. DPP6 loss impacts hippocampal synaptic development and induces behavioral impairments in recognition, learning and memory. *Front. Cell. Neurosci.* 12:84.

33. Han, W., J. Li, …, W. Lu. 2019. Shisa7 is a $GABA_A$ receptor auxiliary subunit controlling benzodiazepine actions. *Science.* 366:246–250.

34. Hirokawa, N., Y. Noda, …, S. Niwa. 2009. Kinesin superfamily motor proteins and intracellular transport. *Nat. Rev. Mol. Cell Biol.* 10:682–696.

35. Jensen, C. S., H. B. Rasmussen, and H. Misonou. 2011. Neuronal trafficking of voltage-gated potassium channels. *Mol. Cell. Neurosci.* 48:288–297.

36. Kreutzberg, G. W. 1969. Neuronal dynamics and axonal flow. IV. Blockage of intra-axonal enzyme transport by colchicine. *Proc. Natl. Acad. Sci. USA.* 62:722–728.

37. Fink, B. R., M. R. Byers, and M. E. Middaugh. 1973. Dynamics of colchicine effects on rapid axonal transport and axonal morphology. *Brain Res.* 56:299–311.

38. Andreu, J. M., and S. N. Timasheff. 1982. Tubulin bound to colchicine forms polymers different from microtubules. *Proc. Natl. Acad. Sci. USA.* 79:6753–6756.

39. Bressloff, P. C., and J. M. Newby. 2013. Stochastic models of intracellular transport. *Rev. Mod. Phys.* 85:135–196.

40. Bressloff, P. C. 2014. Stochastic Processes in Cell Biology, Volume 41. Springer International Publishing, Germany.

41. Fisher, M. E., and A. B. Kolomeisky. 1999. The force exerted by a molecular motor. *Proc. Natl. Acad. Sci. USA.* 96:6597–6602.

42. Fisher, M. E., and A. B. Kolomeisky. 2001. Simple mechanochemistry describes the dynamics of kinesin molecules. *Proc. Natl. Acad. Sci. USA.* 98:7748–7753.

43. Kolomeisky, A. B., and M. E. Fisher. 2007. Molecular motors: a theorist's perspective. *Annu. Rev. Phys. Chem.* 58:675–695.

44. García-López, V., F. Chen, …, J. M. Tour. 2017. Molecular machines open cell membranes. *Nature.* 548:567–572.

45. Cai, X., C. W. Liang, …, S. M. Thompson. 2004. Unique roles of SK and Kv4.2 potassium channels in dendritic integration. *Neuron.* 44:351–364.

46. Smith, D. A., and R. M. Simmons. 2001. Models of motor-assisted transport of intracellular particles. *Biophys. J.* 80:45–68.

47. Voit, E. O., H. A. Martens, and S. W. Omholt. 2015. 150 years of the mass action law. *PLoS Comput. Biol.* 11:e1004012.

48. Kapitein, L. C., M. A. Schlager, …, C. C. Hoogenraad. 2010. Mixed microtubules steer dynein-driven cargo transport into dendrites. *Curr. Biol.* 20:290–299.

49. Nakata, T., and N. Hirokawa. 2003. Microtubules provide directional cues for polarized axonal transport through interaction with kinesin motor head. *J. Cell Biol.* 162:1045–1055.

50. Kuznetsov, A. V. 2014. Sorting of cargos between axons and dendrites: modelling of differences in cargo transport in these two types of neurites. *Comput. Methods Biomech. Biomed. Engin.* 17:792–799.

51. Jensen, C. S., S. Watanabe, …, H. Misonou. 2014. Specific sorting and post-Golgi trafficking of dendritic potassium channels in living neurons. *J. Biol. Chem.* 289:10566–10581.

52. Jin, E. J., F. R. Kiral, and P. R. Hiesinger. 2018. The where, what, and when of membrane protein degradation in neurons. *Dev. Neurobiol.* 78:283–297.

53. Nestor, M. W., and D. A. Hoffman. 2012. Differential cycling rates of Kv4.2 channels in proximal and distal dendrites of hippocampal CA1 pyramidal neurons. *Hippocampus.* 22:969–980.

54. Aljaberi, S., T. O'Leary, and F. Forni. 2019. Qualitative behavior and robustness of dendritic trafficking. *In* 2019 IEEE 58th Conference on Decision and Control (CDC). IEEE, pp. 6628–6633.

55. Magee, J. C. 1998. Dendritic hyperpolarization-activated currents modify the integrative properties of hippocampal CA1 pyramidal neurons. *J. Neurosci.* 18:7613–7624.

56. Noam, Y., Q. Zha, …, T. Z. Baram. 2010. Trafficking and surface expression of hyperpolarization-activated cyclic nucleotide-gated channels in hippocampal neurons. *J. Biol. Chem.* 285:14724–14736.

57. Sartori, F., A.-S. Hafner, …, T. Tchumatchenko. 2020. Statistical laws of protein motion in neuronal dendritic trees. *Cell Rep.* 33:108391.

58. Chu, P. J., J. F. Rivera, and D. B. Arnold. 2006. A role for Kif17 in transport of Kv4.2. *J. Biol. Chem.* 281:365–373.

59. Rivera, J. F., S. Ahmad, …, D. B. Arnold. 2003. An evolutionarily conserved dileucine motif in Shal K+ channels mediates dendritic targeting. *Nat. Neurosci.* 6:243–250.

60. Lewis, T. L., Jr., T. Mao, …, D. B. Arnold. 2009. Myosin-dependent targeting of transmembrane proteins to neuronal dendrites. *Nat. Neurosci.* 12:568–576.

61. Ruiz-Gomez, A., B. Mellström, …, J. R. Naranjo. 2007. G protein-coupled receptor kinase 2-mediated phosphorylation of downstream regulatory element antagonist modulator regulates membrane trafficking of Kv4.2 potassium channel. *J. Biol. Chem.* 282:1205–1215.

62. Maffie, J., and B. Rudy. 2008. Weighing the evidence for a ternary protein complex mediating A-type K+ currents in neurons. *J. Physiol.* 586:5609–5623.

63. Lin, L., L. K. Long, …, D. A. Hoffman. 2014. DPP6 domains responsible for its localization and function. *J. Biol. Chem.* 289:32153–32165.

64. Duménieu, M., M. Oulé, …, J. Lopez-Rojas. 2017. The segregated expression of voltage-gated potassium and sodium channels in neuronal membranes: functional implications and regulatory mechanisms. *Front. Cell. Neurosci.* 11:115.

Biophysical Journal, Volume *120*

Supplemental information

Paradoxical relationships between active transport and global protein distributions in neurons

Adriano Bellotti, Jonathan Murphy, Lin Lin, Ronald Petralia, Ya-Xian Wang, Dax Hoffman, and Timothy O'Leary

Paradoxical relationships between active transport and global protein distributions in neurons - Supplementary Material

A. Bellotti, J. Murphy. L. Lin, R. Petralia,
Y-X Wang, D. Hoffman, T. O'Leary

March 18, 2021

Supplementary Material

An online supplement to this article can be found by visiting BJ Online at http://www.biophysj.org.

Derivation of drift-diffusion equation

Here we derive the drift-diffusion equation in one dimension from a random walk.

Random walk master equation

We begin with a one dimension line that is discretized in space with index $x = 1, 2, ..., X$ and in time with index $n = 1, 2, ..., N$. We are observing a random walk, where a random walker has a probability of f that a step is taken to the right (forwards) and b that a step is taken to the left (backwards). Each step therefore increases or decreases space by one distance unit Δx ($x \to x - \Delta x$ or $x \to x + \Delta x$, respectively). Each step also increases time by one time unit ($n \to n + \Delta n$) where Δn is the duration of one time step.

Let $P_n(x)$ be the probability of finding the walker at position x at some time n. We can now write a master equation for the walker occupying position x at time $n + \Delta n$:

$$P_{n+1}(x) = fP_n(x - \Delta x) + bP_n(x + \Delta x) + (1 - f - b)P_n(x) \tag{1}$$

Notice that we allow for the probability that the walker stays in place with probability $1 - f - b$. If we distribute the last term on the RHS, we have

$$P_{n+\Delta n}(x) = fP_n(x - \Delta x) + bP_n(x + \Delta x) + P_n(x) - fP_n(x) - bP_n(x)$$

Subtract the term $P_n(x)$ from both sides.

$$P_{n+\Delta n}(x) - P_n(x) = fP_n(x-\Delta x) + bP_n(x+\Delta x) - bP_n(x) - fP_n(x) \tag{2}$$

We leave the LHS of Equation 2 as it is now before converting to continuous time and space after working on the RHS.

The RHS of Equation 2 is algebraically modified to a form related to the finite difference approximations of the first and second spatial derivatives. In order to do this, the first and second terms of the RHS of Equation 2 are expanded as follows:

$$pP_n(x-\Delta x) = \frac{1}{2}fP_n(x-\Delta x) - -\frac{1}{2}pP_n(x-\Delta x)$$

$$bP_n(x+\Delta x) = \frac{1}{2}bP_n(x+\Delta x) - -\frac{1}{2}bP_n(x+\Delta x)$$

Additionally, the following terms summing to zero are added to the RHS of Equation 2

$$\frac{1}{2}fP_n(x+\Delta x) - \frac{1}{2}fP_n(x+\Delta x) + \frac{1}{2}bP_n(x-\Delta x) - \frac{1}{2}bP_n(x-\Delta x)$$

All terms are added to Equation 2 and expansions are substituted into Equation 2. Rearrangement of terms and factoring puts the equation in the desired form, as follows:

$$\begin{aligned}
P_{n+\Delta n}(x) - P_n(x) &= fP_n(x-\Delta x) + bP_n(x+\Delta x) - fP_n(x) - bP_n(x) \\
&= \frac{1}{2}fP_n(x-\Delta x) - fP_n(x) + \frac{1}{2}pP_n(x+\Delta x) + \frac{1}{2}bP_n(x-\Delta x) - bP_n(x) \\
&\quad + \frac{1}{2}bP_n(x+\Delta x) - \frac{1}{2}fP_n(x+\Delta x) + \frac{1}{2}fP_n(x-\Delta x) + \frac{1}{2}bP_n(x+\Delta x) \\
&\quad - \frac{1}{2}bP_n(x-\Delta x) \\
&= \frac{1}{2}\Big(fP_n(x-\Delta x) - 2fP_n(x) + fP_n(x+\Delta x) + bP_n(x-\Delta x) - 2bP_n(x) \\
&\quad + bP_n(x+\Delta x)\Big) - \frac{1}{2}\Big(fP_n(x+\Delta x) - fP_n(x-\Delta x) - bP_n(x+\Delta x) \\
&\quad + bP_n(x-\Delta x)\Big) \\
P_{n+\Delta n}(x) - P_n(x) &= \frac{1}{2}(f+b)\Big(P_n(x-\Delta x) - 2P_n(x) + P_n(x+\Delta x)\Big) \\
&\quad - \frac{1}{2}(f-b)\Big(P_n(x+\Delta x) - P_n(x-\Delta x)\Big) \qquad (3)
\end{aligned}$$

The LHS of Equation 3 will become the time derivative of P, and the first and second terms on the RHS will become second and first spatial derivatives of P, respectively.

Finite difference approximation

As the spatial and temporal step sizes Δx and Δn approach zero, Equation 3 approaches the continuous drift-diffusion equation. In order to approximate the form of the derivatives in continuous space and time, we can use the finite difference approximations for first and second derivatives. We begin with the forward difference equation for a first derivative of some arbitrary function $f(t)$:

$$\frac{df}{dt} = \frac{f(t+\Delta t) - f(t)}{\Delta t} \tag{4}$$

We can also consider the central difference equation for the first derivative of $f(t)$:

$$\frac{df}{dt} = \frac{f(t+\frac{1}{2}\Delta t) - f(t-\frac{1}{2}\Delta t)}{\Delta t}$$

Since the smallest step size in our discrete case was Δt and not $\frac{1}{2}\Delta t$, it might be more helpful to take a central difference approximation over two steps $2\Delta t$:

$$\frac{df}{dt} = \frac{f(t+\Delta t) - f(t-\Delta t)}{2\Delta t} \tag{5}$$

The central difference approximation can then be applied to a second derivative using the chain rule:

$$\frac{d^2 f}{dt^2} = \frac{\frac{d}{dt}f(t+\frac{1}{2}\Delta t) - \frac{d}{dt}f(t-\frac{1}{2}\Delta t)}{\Delta t}$$

$$\frac{d^2 f}{dt^2} = \frac{1}{\Delta t}\Big(\frac{f(t+\Delta t) - f(t)}{\Delta t} - \frac{f(t) - f(t-\Delta t)}{\Delta t}\Big)$$

$$\frac{d^2 f}{dt^2} = \frac{f(t+\Delta t) - 2f(t) + f(t-\Delta t)}{\Delta t^2} \tag{6}$$

The approximations in Equations 4, 5, and 6 can be used to write discrete difference equations as continuous derivatives.

Discrete to continuous space and time

In order to modify our master equation in Equation 3 to continuous space and time, all terms must be in the form of Equations 4, 5, or 6. We can multiply the LHS by unit value $\frac{\Delta n}{\Delta n}$, the first term on the RHS by unit $\frac{\Delta x^2}{\Delta x^2}$, and the second

term on the RHS by unit $\frac{\Delta x}{\Delta x}$:

$$
\begin{aligned}
\Big(P_{n+\Delta n}(x) - P_n(x)\Big)\frac{\Delta n}{\Delta n} &= \frac{1}{2}\frac{\Delta x^2}{\Delta x^2}(f+b)\Big(P_n(x-\Delta x) - 2P_n(x) + P_n(x+\Delta x)\Big) \\
&\quad - \frac{1}{2}\frac{\Delta x}{\Delta x}(f-b)\Big(P_n(x+\Delta x) - P_n(x-\Delta x)\Big) \\
\frac{P_{n+\Delta n}(x) - P_n(x)}{\Delta n}\Delta n &= \frac{\Delta x^2}{2}(f+b)\frac{P_n(x-\Delta x) - 2P_n(x) + P_n(x+\Delta x)}{\Delta x^2} \\
&\quad - \frac{\Delta x}{2}(f-b)\frac{P_n(x+\Delta x) - P_n(x-\Delta x)}{\Delta x} \\
\frac{P_{n+\Delta n}(x) - P_n(x)}{\Delta n} &= \frac{\Delta x^2(f+b)}{2\Delta n}\frac{P_n(x-\Delta x) - 2P_n(x) + P_n(x+\Delta x)}{\Delta x^2} \\
&\quad - \frac{\Delta x(f-b)}{\Delta n}\frac{P_n(x+\Delta x) - P_n(x-\Delta x)}{2\Delta x}
\end{aligned}
$$

We can now make derivative approximations using Equations 4, 5, and 6 and replace discrete time n and space x with continuous time t and space x.

$$
\frac{\partial P(x,t)}{\partial t} = D\frac{\partial^2 P(x,t)}{\partial x^2} + v\frac{\partial P(x,t)}{\partial x}
$$

where $P(x,t)$ is the probability of finding a random walker at position x at time t in continuous space and time. We have also identified $D = \frac{\Delta x^2(f+b)}{2\Delta n}$ and $v = \frac{\Delta x(f-b)}{\Delta n}$ as $\lim_{\Delta x\to 0}$ and $\lim_{\Delta n\to 0}$. For a population that contains a total of T random walkers, the concentration of particles in some segment of line can be defined as $c(x,t) = TP(x,t)/\Delta x$ using the law of large numbers. Substituting this into our equation produces the one-dimensional drift-diffusion equation for a large population of particles in a more recognizable form:

$$
\frac{\partial c(x,t)}{\partial t} = D\frac{\partial^2 c(x,t)}{\partial x^2} + v\frac{\partial c(x,t)}{\partial x} \tag{7}
$$

Distribution of kinetic measures in stochastic model

In this section, the stochastic model is analyzed as a modified discrete time random walk. We aim to realize the distributions of the experimental measures of puncta kinetics (Figure 5C). Among the distributions derived here are distance traveled and mean speed.

Total distance traveled

Until absorption, a puncta in this stochastic model behaves as in a one-dimensional unbiased random walk. We use this premise to solve for the distributions of the kinetic measures of interest.

We begin with total distance traveled D_{tot}, which is a measure of the puncta's final position relative to its origin. The final position is the site of absorption, which ends the random walk. The expected value of an unbiased

random walk is 0, regardless of the number of time steps n. For a biased random walk with rightward (+1) propensity f and leftward (-1) propensity b, the expected value after n time steps is $n(f-b)$. The variability around this expected value scales with $\sqrt{n}$. As the number of puncta simulated approaches infinity, the distribution of their final positions - by central limit theorem - approximates a bell curve. In a DTRW with no absorption, this is well approximated by a normal distribution. We also find that unidirectional runs (with increasing parameter p_{mem}) increase the the standard deviation σ of the normal distribution approximation [1, 2].

In a DTRW with absorption (also called decay, degradation, sink, etc.), the shape of the distribution changes. To estimate the total distance traveled by puncta on a 1D lattice with absorption, we use a differential equation for diffusion. A random walk with more than a few steps is well approximated by diffusion [1]. The PDE for diffusion with decay and source is

$$\frac{\partial c}{\partial t} = D\frac{\partial^2 c}{\partial x^2} - K_{\text{off}}c + s \tag{8}$$

where $c = c(x,t)$ is the concentration of puncta at some position x at time t, D is the diffusion coefficient, K_{off} is the absorption rate, and s is puncta source at $x = 0$. The distribution of net displacement of puncta is approximated by the steady-state ($\frac{\partial c}{\partial t} = 0$) of Eq. 8:

$$0 = D\frac{\partial^2 c}{\partial x^2} - K_{\text{off}}c$$

Solving this produces two exponentials of the form:

$$c(x) = Ae^{\lambda x} + Be^{-\lambda x} \tag{9}$$

where λ is a space constant for spread of puncta before absorption: $\lambda = \sqrt{K_{\text{off}}/D}$. If we next impose the restriction that $c \to 0$ as $x \to \pm\infty$, then Eq. 9 is restricted to

$$c(x) = Ae^{\lambda x} \qquad \text{for } x < 0$$
$$c(x) = Be^{-\lambda x} \qquad \text{for } x > 0$$

For continuity at $c(0)$, $A = B$. Solving for this single coefficient $A = B$ requires the amount of mass M released at the source at $c = 0$. This quantity M in units of quantity per cross sectional area per time is split into left and right directions, therefore

$$A = B = \frac{M}{2D\lambda} = \frac{M}{2\sqrt{DK_{\text{off}}}}$$

. The full symmetric solution is

$$c(x) = \frac{M}{2\sqrt{DK_{\text{off}}}}\exp\sqrt{K_{\text{off}}/D}x \qquad \text{for } x < 0$$
$$c(x) = \frac{M}{2\sqrt{DK_{\text{off}}}}\exp-\sqrt{K_{\text{off}}/D}x \qquad \text{for } x > 0$$

In our experiments, the orientation of axons was not always clear. We therefore report this measure as distance traveled rather than net displacement, since we can only account for magnitude of displacement and not direction. The distribution for this measure of total distance traveled is thus symmetric about the y-axis:

$$c(x) = \frac{M}{\sqrt{DK_{\text{off}}}} \exp -\sqrt{K_{\text{off}}/D}x \qquad \text{for } x > 0 \tag{10}$$

For a DTRW, the diffusion coefficient can be approximated as $D = 2(\Delta x)^2 p_+ p_- / \Delta t$, where Δx and Δt are the discrete steps in space and time, and p_+ and p_- are right and left jump probabilities [3]. The total punctal distance traveled as computed in our experimental kinetic measure is follows a distribution with the form of Eq. 10, a monotonic decreasing function.

Average speed

The next kinetic measure for which we derive a probability distribution is average speed v. As a kinetic measure, v is computed as distance traveled divided by total puncta run time.

As before, we assume that a DTRW with more than a few steps is well approximated by diffusion [1]. We can then solve the diffusion equation

$$\frac{\partial c}{\partial t} = D \frac{\partial^2 c}{\partial x^2}$$

for diffusion propagator $c(x, t)$

$$c(x, t) = \frac{1}{\sqrt{4\pi Dt}} \exp\Big(-\frac{x^2}{4Dt} - K_{\text{off}} t \Big)$$

We use $c(x, t)$ to compute the probability of puncta at position x at time t: $p(x|t)$.

$$\begin{aligned} p(x|t) &= \frac{K_{\text{off}} c(x,t)}{\int_{-\infty}^{\infty} K_{\text{off}} c(x,t) dx} \\ &= \frac{K_{\text{off}} \frac{1}{\sqrt{4\pi Dt}} \exp\Big(-\frac{x^2}{4Dt} - K_{\text{off}} t \Big)}{\int_{-\infty}^{\infty} K_{\text{off}} \frac{1}{\sqrt{4\pi Dt}} \exp\Big(-\frac{x^2}{4Dt} - K_{\text{off}} t \Big) dx} \end{aligned}$$

We cancel terms in the numerator and denominator and solve the integral:

$$\begin{aligned} p(x|t) &= \frac{\exp\Big(-\frac{x^2}{4Dt} \Big)}{\int_{-\infty}^{\infty} \exp\Big(-\frac{x^2}{4Dt} \Big) dx} \\ &= \frac{\exp -\frac{x^2}{4Dt}}{\sqrt{4\pi Dt}} \end{aligned}$$

Note that $p(x|t)$ is independent of K_{off}

With $p(x|t)$, we can now compute the probability of a given puncta velocity v at time t: $p(v|t)$

$$p(v|t) = \int_{-\infty}^{\infty} \delta(v - \frac{|x|}{t})p(x|t)dx$$

where δ is the Dirac delta function. This integral sweeps through all positions x to find the probability of puncta at least location ($p(x|t)$) that matches each speed ($\delta(v - \frac{|x|}{t})$. We proceed using the scaling, symmetry, and translation properties of the Dirac delta function:

$$\begin{aligned}
p(v|t) &= \int_{-\infty}^{\infty} \delta(v - \frac{|x|}{t})p(x|t)dx \\
&= \int_{-\infty}^{\infty} \delta\big(\frac{1}{t}(vt - |x|)\big)p(x|t)dx \\
&= \int_{-\infty}^{\infty} t\delta(vt - |x|)p(x|t)dx \\
&= t\int_{-\infty}^{\infty} \delta(vt - |x|)p(x|t)dx \\
&= t\Big(p(\delta t|t) + p(-\delta t|t)\Big) \\
&= \frac{2t}{\sqrt{4\pi Dt}}e^{-(vt)^2/4Dt} \\
p(v|t) &= \frac{t}{\sqrt{\pi D}}e^{-v^2t/4D}
\end{aligned}$$

To check this probability distribution, we integrate it over the entire domain to ensure it sums to 1:

$$\int_0^{\infty} p(v|t)dv = \frac{1}{2}\int_{-\infty}^{\infty} p(v|t)dv = \frac{1}{2}\sqrt{\frac{t}{\pi D}}\int_{-\infty}^{\infty} \exp -\frac{v^2t}{4D}dv = \frac{1}{2}\sqrt{\frac{t}{\pi D}}\sqrt{\frac{4\pi D}{t}} = 1$$

We next compute the expected time until absorption $\phi(t)$, which is approximated as

$$\phi(t) = K_{\text{off}}e^{-K_{\text{off}}t}$$

With $p(v|t)$ and $\phi(t)$, we can now compute the probability distribution for average speed $F(v)$. We integrate the velocity distribution at a fixed time

$(p(v|t))$ multiplied by the fraction of particles $(\phi(t))$ for all time $t > 0$:

$$
\begin{aligned}
F(v) &= \int_0^\infty p(v|t)\phi(t)dt \\
&= \int_0^\infty \Big(\frac{t}{\sqrt{\pi D}} e^{-v^2 t/4D}\Big)\Big(K_{\text{off}} e^{-K_{\text{off}} t}\Big) dt \\
&= \frac{K_{\text{off}}}{\sqrt{\pi D}} \int_0^\infty \sqrt{t} e^{-(v^2/4D+k)t} dt \\
&= \frac{K_{\text{off}}}{\sqrt{\pi D}} \frac{\sqrt{\pi}}{2} (\frac{v^2}{4D} + K_{\text{off}})^{-3/2} \\
&= \frac{K_{\text{off}}}{2\sqrt{D}(\frac{v^2}{4D} + K_{\text{off}})^{-3/2}}
\end{aligned}
$$

If we substitute $v_0 = 2\sqrt{DK_{\text{off}}}$,

$$
F(v) = \frac{1}{v_0}\Big(\frac{v^2}{v_0^2} + 1\Big)^{-3/2} \tag{11}
$$

To confirm our result, we can compute its integral for all $v \geq 0$:

$$
\int_0^\infty F(v)dv = 1
$$

Like $c(x)$, $F(v)$ is a monotonically decreasing function. Increasing D and/or K_{off} increases the tailedness of the distribution. We can compute the expected value of mean particle speed $\langle v \rangle$ as follows:

$$
\langle v \rangle = \int_0^\infty vF(v)dv = v_0 = 2\sqrt{DK_{\text{off}}}
$$

This shows that $\langle v \rangle$ increases with increasing D and/or K_{off}

Statistical inference using stochastic model

Here we describe our method for statistical inference of puncta behavior based on experimental observations. In broad terms, we estimate parameters p_{off} and p_{mem} from our stochastic model using the observed distributions of kinetic measurements.

We interpret our experimental measures of puncta kinetics (Figure 5C) as evidence of puncta behavior. We have developed a stochastic model of puncta transport based on a modified random walk (Figure S8A), consistent with observed intracellular transport [4, 5, 6]. Our model produces simulated data similar to our experiments (compare Figures S7 and S8). We aim to infer whether the observed differences between axons and dendrites can result from a difference in p_{off} or p_{mem}. To this end, we perform a model fit to data from axons and dendrites. We then compare a model fit using p_{off} alone as well as with p_{off} and p_{mem}.

Maximum likelihood estimation

Maximum likelihood estimation (MLE) is a standard method of estimating the parameters of a model such that the model's output is the most probable match to some observed data. In this method, a likelihood equation is derived from the joint probability distribution of simulated and observed data as a function of model parameters. The set of parameters that maximizes the likelihood equation produces the best fit between the simulated and observed data.

We first explore the direct estimation of p_{off} and p_{mem} for which our stochastic model most closely reproduces our observed data. We then estimate parameters that reproduce a distribution that estimates our observed data—a less direct but simpler method.

We begin by defining the likelihood function:

$$\mathcal{L}(p_{\text{off}}, p_{\text{mem}} \mid \text{observed data}) = P(\text{transport producing observed data} \mid p_{\text{off}}, p_{\text{mem}}) \tag{12}$$

where the likelihood $\mathcal{L}$ of parameters $p_{\text{off}}, p_{\text{mem}}$ producing the observed data is equal to the probability of the actual physical transport mechanism producing the observed data given those parameters $p_{\text{off}}, p_{\text{mem}}$. Defining the probability in Eq. 12 is difficult for a few reasons. First, the observed data consists of sets of net displacements δ, mean speeds s, stall fractions ϵ, and diffusivities α for $N_a = 961$ puncta in axons and $N_d = 507$ puncta in dendrites:

$$\delta_1, \delta_2, ..., \delta_{N_a-1}, \delta_{N_a} \qquad \delta_1, \delta_2, ..., \delta_{N_d-1}, \delta_{N_d} \tag{13}$$

$$s_1, s_2, ..., s_{N_a-1}, s_{N_a} \qquad s_1, s_2, ..., s_{N_d-1}, s_{N_d} \tag{14}$$

$$\epsilon_1, \epsilon_2, ..., \epsilon_{N_a-1}, \epsilon_{N_a} \qquad \epsilon_1, \epsilon_2, ..., \epsilon_{N_d-1}, \epsilon_{N_d} \tag{15}$$

$$\alpha_1, \alpha_2, ..., \alpha_{N_a-1}, \alpha_{N_a} \qquad \alpha_1, \alpha_2, ..., \alpha_{N_d-1}, \alpha_{N_d} \tag{16}$$

This observed data is a large combination of variables. Further, these variables (Eqs. 13-16) are not measured directly; they are computed from the physical coordinates of trajectories from individual puncta. Computing s, ϵ, and α involves the trajectories themselves, not just the final position. The random process in our model is time-dependent (non-stationary) and non-ergodic, so deriving the statistics (expected value and variance) does not reveal the time averages. The expected value itself would only be useful in computing net displacements (Eq. 13), since this is the only kinetic measure computed using solely outcome of the random process without the full trajectory. Since a key parameter of our system p_{mem} abruptly ends the random process, the statistics are not as simple as those of a standard random walk. These nontrivial derivations only produce the likelihood function, which then requires differentiation w.r.t. p_{off} and p_{mem} for an analytical solution to this problem. For these reasons, rather than deriving the statistics and time-averages of our model, we opt for a numerical approach to this problem.

Instead of deriving equations for δ, s, ϵ, and α, we can estimate these probability distributions of these kinetic measures by averaging the behavior of simulated puncta. Simulating a large number of puncta N_s is crucial given the

stochasticity of the model. From N_s simulated trajectories with a given p_{off} and p_{mem}, we can then compute:

$$\delta_1, \delta_2, ..., \delta_{N_s-1}, \delta_{N_s} \tag{17}$$
$$s_1, s_2, ..., s_{N_s-1}, s_{N_s} \tag{18}$$
$$\epsilon_1, \epsilon_2, ..., \epsilon_{N_s-1}, \epsilon_{N_s} \tag{19}$$
$$\alpha_1, \alpha_2, ..., \alpha_{N_s-1}, \alpha_{N_s} \tag{20}$$

With a sufficiently large N_s, we obtain averages of δ, s, ϵ, and α. This method follows the law of large numbers, whereby the average of a large number of iterations approaches the expected behavior of the random process. We can then compare simulated data (Eqs. 17-20) to observed data (Eqs. 13-16) and optimize for parameter set p_{off} and p_{mem} that minimizes this variation. This semi-empirical approach employs both model simulation to estimate kinetic distributions and optimization to minimize the difference between simulated and observed data.

MLE is still applicable in this approach. Approximating simulated and observed data as probability distributions simplifies the optimization problem. Both observed and simulated sample sets can be approximated as probability distributions, and the distance between distribution parameters is minimized. MLE is a suitable method for fitting observed and simulated data to a distribution with a defined probability density function (pdf).

The pdf of two kinetic measures δ (Eq. 10) and s (Eq. 11) are derived in the previous section. Both are monotonically decreasing functions with domain $[0, \infty)$. The pdf of the other measures ϵ and α are seemingly intractable and beyond the scope of this paper. We therefore approximate the distributions of kinetic measures using an estimate likelihood with the gamma distribution. The gamma distribution can accommodate the monotonic decreasing pdfs derived here and is flexible for all simulated and observed data (Eqs. 17-20 and Eqs. 13-16 depicted in Figure 5C). The gamma distribution is appropriate because it is continuous and covers a semi-infinite $[0, \infty)$ interval. A gamma distribution has shape k and scale θ, which are fit to both observed and simulated data.

To this end, we derive a function for the likelihood of the gamma distribution with parameters k and θ producing observed or simulated data. As an example, we estimate k and θ that best match the observed mean speed s in axons:

$$\mathcal{L}(k, \theta \mid X = s_1, ..., s_{N_a}) = P(\text{gamma distribution fits X} \mid k, \theta)$$

Here, the probability in the RHS is, by definition, the pdf of the gamma distribution:

$$\mathcal{L}(k, \theta \mid X = s_1, ..., s_{N_a}) = \frac{1}{\Gamma(k)\theta^k} X^{k-1} e^{x/\theta} \tag{21}$$

where vector X is the data to which a gamma distribution is fit. The RHS of Eq. 21 is the pdf of the gamma distribution, where Γ is the gamma function: $\Gamma(g) = \int_0^\infty z^{g-1} e^{-z} dz$.

The remaining steps for MLE involve calculating the log-likelihood $\ell(k, \theta \mid X)$, taking partial derivatives w.r.t. each parameter $\partial\ell(k,\theta)/\partial k$ and $\partial\ell(k,\theta)/\partial\theta$, setting to zero, and solving for both k and θ. The solution for MLE using the gamma distribution has no closed-form expression [7, 8]. Rather, a numerical solution is computed. We use MATLAB function `fitdist`, which solves the following system of two equations:

$$\ln k - \psi(k) = \ln\left(\frac{(\sum_{i=1}^{N_a} s_i)/N_a}{(\prod_{i=1}^{N_a} s_i)^{1/N_a}}\right) \quad , \quad \theta = \frac{(\sum_{i=1}^{N_a} s_i)/N_a}{k}$$

where ψ is the digamma function: $\psi(g) = \frac{d}{dx}\ln(\Gamma(g))$.

MLE for gamma distribution parameters is performed for each set of observed data (Eqs. 13-16) as well as for N_s trajectories simulated using the stochastic model (Eqs. 17-20) with a given p_{off} and p_{mem}. Observed data for net displacement and average speed are normalized between 0 and 1. All resultant gamma parameters are:

$$k_{\delta,a}, \theta_{\delta,a} \qquad k_{s,a}, \theta_{s,a} \qquad k_{\epsilon,a}, \theta_{\epsilon,a} \qquad k_{\alpha,a}, \theta_{\alpha,a} \tag{22}$$

$$k_{\delta,d}, \theta_{\delta,d} \qquad k_{s,d}, \theta_{s,d} \qquad k_{\epsilon,d}, \theta_{\epsilon,d} \qquad k_{\alpha,d}, \theta_{\alpha,d} \tag{23}$$

$$k_{\delta,s}, \theta_{\delta,s} \qquad k_{s,s}, \theta_{s,s} \qquad k_{\epsilon,s}, \theta_{\epsilon,s} \qquad k_{\alpha,s}, \theta_{\alpha,s} \tag{24}$$

where each variable k or θ denotes gamma shape or scale parameter, the first subscript denotes kinetic measure, and the second subscript denotes source of the distribution (axons, dendrites, or simulation). The gamma distributions of kinetic measures in axons and dendrites (Eqs. 22 and 23) are plotted over the raw data in Figure 5C. We now have a succinct description of each distribution using two rather than N_a, N_d, or N_s terms. This framework allows us to compare observed and simulated data. We next use a least squares method fit simulation parameters p_{off} and p_{mem} to the experimental data.

Least squares method of optimization

MLE allows for a representation of data from axons, dendrites, or simulations as a gamma distribution using only two parameters k and θ. We now aim to fit our stochastic model to experimental data. A standard approach in model fitting is the least squares method (LSM) of regression. Here, we describe LSM in the context of our problem. We then outline numerical algorithms for solving nonlinear LSM problems.

We describe a model M with output y as a function of independant variable x and adjustable parameters β:

$$y = M(x, \beta)$$

We substitute an example optimization problem using our data and parameters,

$$[k_{\delta,s}, \theta_{\delta,s}] = M(p_{\text{off}}, p_{\text{mem}}) \tag{25}$$

where M is the stochastic model, p_{off} and p_{mem} are the adjustable parameters that are tweaked for an optimal fit with output $y = [k_{\delta,s}, \theta_{\delta,s}]$. Model fitting can be applied to any number of outputs from Eq. 24. Note that our function for optimization in Eq. 25 differs from that in curve fitting in that it takes no independent variables x. The model fitting in our example fits only the model parameters. Further, M itself is a complex, nonlinear function. Contained within M is a simulation of the N_s puncta trajectories using the stochastic model (Figure S8). Also contained within M is computation of kinetic measures (δ, s, ϵ, and α) for each of the N_s trajectories. Lastly contained within M is the MLE of the gamma parameters (k and θ) from the distributions of kinetic measures (Eqs. 17-20). Selection of N_s is therefore a balance between computational cost (run time) and accuracy of expected puncta behavior (by law of large numbers). We choose $N_s = 10,000$ puncta in each iteration of M.

The quality of fit for the output of M is measured by the size of the residuals, or the difference between the observed data and estimated data:

$$\text{residuals} = \text{observed data} - \text{simulated data}$$

In the context of our problem, we will fit the output in Eq. 25 to observed data in axons:

$$\begin{aligned} r &= [k_{\delta,a}, \theta_{\delta,a}] - M(p_{\text{off}}, p_{\text{mem}}) \\ r &= [k_{\delta,a}, \theta_{\delta,a}] - [k_{\delta,s}, \theta_{\delta,s}] \end{aligned} \tag{26}$$

where $[k_{\delta,a}, \theta_{\delta,a}]$ is the axonal data, and r is a vector of residuals, $r = [r_1, r_2]$. The LSM aims to minimize the sum of the squares S of the residuals: $S = \sum r_i^2$. S is minimized by setting its gradient to zero. This involves taking a partial derivative of S w.r.t. each parameter. In our example:

$$\begin{aligned} \frac{\partial S}{\partial p_{\text{off}}} &= \frac{\partial}{\partial p_{\text{off}}} \sum_i r_i^2 = 0 & \qquad \frac{\partial S}{\partial p_{\text{mem}}} &= \frac{\partial}{\partial p_{\text{mem}}} \sum_i r_i^2 = 0 \\ &= 2 \sum_i r_i \frac{\partial r_i}{\partial p_{\text{off}}} = 0 & &= 2 \sum_i r_i \frac{\partial r_i}{\partial p_{\text{mem}}} = 0 \end{aligned}$$

Using Eq. 26 and given that the partial derivatives of $[k_{\delta,a}, \theta_{\delta,a}]$ w.r.t. p_{off} and p_{mem} is zero,

$$\frac{\partial S}{\partial p_{\text{off}}} = -2 \sum_i r_i \frac{\partial M}{\partial p_{\text{off}}} = 0 \qquad \frac{\partial S}{\partial p_{\text{mem}}} = -2 \sum_i r_i \frac{\partial M}{\partial p_{\text{mem}}} = 0 \tag{27}$$

A closed-form solution for Eq. 27, as in most non-linear least squares problems, does not exist. A numerical algorithm is instead used to minimize S.

There are several algorithms for nonlinear curve-fitting and data-fitting in the least squares sense. We use MATLAB function `lsqcurvefit`, which employs a damped LSM, also known as the Levenberg–Marquardt algorithm [9, 10]. The damped LSM is a combination of the Gauss–Newton algorithm with a trust region. A broad overview of the damped LSM is presented here.

The damped LSM is an iterative process that begins at a starting point for parameter vector β. For our system, $\beta = [p_{\text{off}}, p_{\text{mem}}]$. The aim is to find the set of β that best fits each of the m data points (x_i, y_i). Again, in our system, we have no independent variables x_i, and we fit outputs of model M to observed data y_i. In each step of the algorithm, β is updated with a new parameter estimate $\beta+\Delta$. To make a sensible modification Δ to the parameter estimate, the nonlinear function M is approximated by linearization (first-order approximation):

$$M(x_i, \beta+\Delta) \approx M(x_i, \beta) + J_i\Delta \qquad , \qquad J_i = \frac{\partial M(\beta)}{\partial \beta}$$

where J_i is the gradient of M w.r.t. β. Using this approximation, we can compute the sum of squares S of the residuals:

$$S(\beta+\Delta) \approx \sum_i^m r_i^2$$

$$S(\beta+\Delta) \approx \sum_i^m \Big(y_i - M(x_i, \beta) - J_i\Delta\Big)^2 \tag{28}$$

As before, Eq. 28 is minimized where its derivative equals zero. The derivative of Eq. 28 w.r.t. Δ is

$$(J^T J)\Delta = J^T\big(y - M(\beta)\big) \tag{29}$$

where J is a Jacobian matrix consisting of rows J_i. Matrix multiplication in Eq. 29 results in a system of linear equations that is solved for Δ. This procedure of linearization, function approximation, and solving for step Δ repeats to progress toward a minimum S. The algorithm stops when step size Δ falls below some preset threshold. The approach as described thus far is the Gauss-Newton method.

The damped LSM has the addition of damping factor λ, a non-negative scalar, as follows:

$$(J^T J + \lambda I)\Delta = J^T\big(y - M(\beta)\big) \tag{30}$$

where I is the identity matrix. When λ is small or zero, the method approximates the Gauss-Newton method. When λ is large, the direction of Δ approaches the direction of steepest descent but with magnitude approaching zero. λ adjusts the size of the step, defining a trust region around the current estimate β that is reevaluated at each iteration. If $S(\beta+\Delta) < S(\beta)$, a successful step toward a minima, λ is decreased and the trust region increased. If $S(\beta+\Delta) > S(\beta)$, λ is increased and the trust region decreased. In this regard, a dynamic λ allows for a search that mediates between steepest descent and the Gauss-Newton method. For instance, a limitation of the Gauss-Newton method arises when second-order terms dominant the gradient, since the Gauss-Newton method relies on first-order approximation. Dampening with λ can ensure descent path more efficient than searching for steepest descent.

We use this algorithm in a series of model fits to our experimental data. Given the stochastic nature of M in our system, we increase the lower threshold for the finite difference step size Δ. This ensures continuous progression toward a global minima despite slight variation in output with repeated evaluations of M. Otherwise, with a low minimum Δ, the algorithm greatly reduces the trust region and terminates at a false local minima—an artifact of the randomness in M.

We first fit

$$[k_{\delta,s}, \theta_{\delta,s}, k_{s,s}, \theta_{s,s}] = M(p_{\text{off}}, p_{\text{mem}} = 0)$$

to the corresponding experimental results in axons ($[k_{\delta,a}, \theta_{\delta,a}, k_{s,a}, \theta_{s,a}]$) and dendrites ($[k_{\delta,d}, \theta_{\delta,d}, k_{s,d}, \theta_{s,d}]$). p_{mem} is constrained to zero, and upper and lower bounds for p_{off} are set to 0 and 1, consistent with the range of a probability. The starting value was $p_{\text{off}} = 0.001$. The result of this fit is depicted in Figure 5C (*second column*). We then fit

$$[k_{\delta,s}, \theta_{\delta,s}, k_{s,s}, \theta_{s,s}, k_{\epsilon,s}, \theta_{\epsilon,s}] = M(p_{\text{off}}, p_{\text{mem}})$$

to the corresponding experimental results in axons and dendrites. We now fit both parameters, and both are bounded between 0 and 1 with starting values $p_{\text{off}} = 0.001$ and $p_{\text{mem}} = 0.01$. The result of this fit is depicted in Figure 5C (*third column*). The goodness of these fits and their implications are discussed in Results.

References

[1] Subrahmanyan Chandrasekhar. Stochastic problems in physics and astronomy. *Reviews of modern physics*, 15(1):1, 1943.

[2] Alex H Williams, Cian O'Donnell, Terrence J Sejnowski, and Timothy O'Leary. Dendritic trafficking faces physiologically critical speed-precision tradeoffs. *eLife*, 5(DECEMBER2016), 12 2016.

[3] Alexander M Berezhkovskii, Leonardo Dagdug, and Sergey M Bezrukov. Exact solutions for distributions of first-passage, direct-transit, and looping times in symmetric cusp potential barriers and wells. *The Journal of Physical Chemistry B*, 123(17):3786–3796, 2019.

[4] Avi Caspi, Rony Granek, and Michael Elbaum. Enhanced Diffusion in Active Intracellular Transport. *Physical Review Letters*, 85(26):5655–5658, 2000.

[5] Avi Caspi, Rony Granek, and Michael Elbaum. Diffusion and directed motion in cellular transport. *Physical Review E - Statistical Physics, Plasmas, Fluids, and Related Interdisciplinary Topics*, 66(1), 2002.

[6] Benjamin M. Regner, Daniel M. Tartakovsky, and Terrence J. Sejnowski. Identifying transport behavior of single-molecule trajectories. *Biophysical Journal*, 107(10):2345–2351, 2014.

[7] S C Choi and R Wette. Maximum likelihood estimation of the parameters of the gamma distribution and their bias. *Technometrics*, 11(4):683–690, 1969.

[8] Gerald J Hahn and Samuel S Shapiro. Statistical models in engineering. Technical report, 1967.

[9] Kenneth Levenberg. A method for the solution of certain non-linear problems in least squares. *Quarterly of Applied Mathematics*, 2(2):164–168, 1944.

[10] Donald W. Marquardt. An Algorithm for Least-Squares Estimation of Nonlinear Parameters. *Journal of the Society for Industrial and Applied Mathematics*, 11(2):431–441, 1963.

Supplementary table

Table S1: Density of immunogold particles identified by electron microscopy in synapses of axons and dendrites.

**Axons**, *N = 624*		**Dendrites**, *N = 646*		*Neurite, number of synapses sampled*
93		211		*Number of gold particles*
0.149		0.327		*Gold particles / synapse*
30.6		69.4		*Percent of total*
Synaptic	*Extrasynaptic*	*Synaptic*	*Extrasynaptic*	*Subdivision*
26	67	68	143	*Number of gold particles*
0.042	0.107	0.105	0.221	*Gold particles / synapse*
28.0	72.0	32.2	67.8	*Percent of subdivision*
8.6	22.0	22.4	47.0	*Percent of total*

Supplementary figures

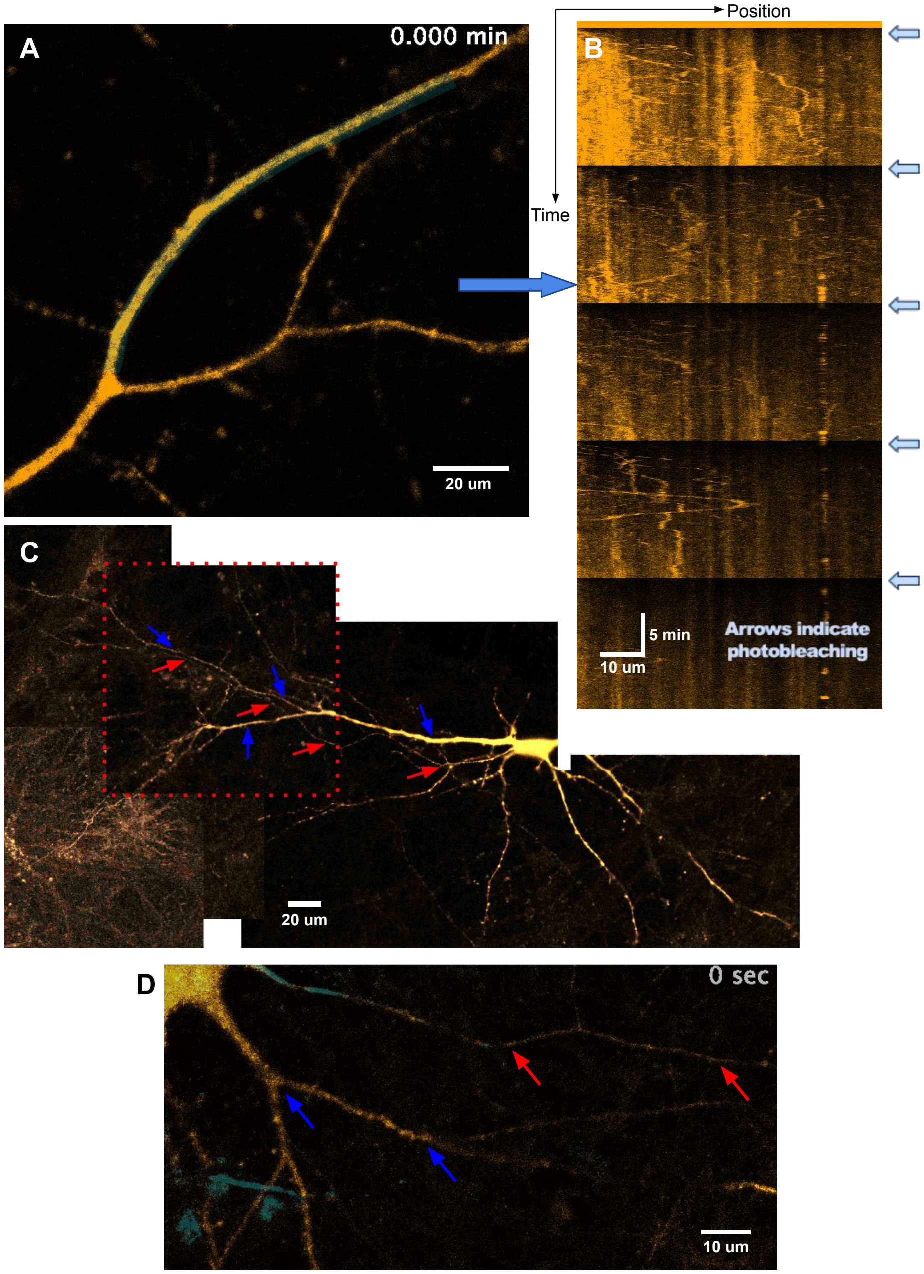

Figure S1: Time series, kymogram generation, and neurite differentiation. (A): A sample time series of Kv4.2-SGFP2 trafficking (orange) depicts a dendrite branch exhibiting frequent transport. The segmented line selection (cyan) surrounding the neurite is used to create kymogram in (B). For animation, see Supplemental Video 1. (B): Kymogram created from segmented line selection in (A). X-axis indicates neurite position and y-axis indicates time. Left-pointing arrows indicate time points for intermittent photobleaching. (C): High-magnification frame for time series (red dotted line) often makes differentiating axons (red arrows) from dendrites (blue arrows) difficult. Low-magnification global images (entire image, stitched) enable neurite differentiation by morphology and branching. (D): Time series depicts characteristic high frequency trafficking in axons (red arrow) compared to dendrites (blue arrow). Anti-GFP-488 (orange) indicates Kv4.2-SGFP2 expression and ankyrin-G (cyan) for axons are used for definitive neurite differentiation. For animation, see Supplemental Video 2.

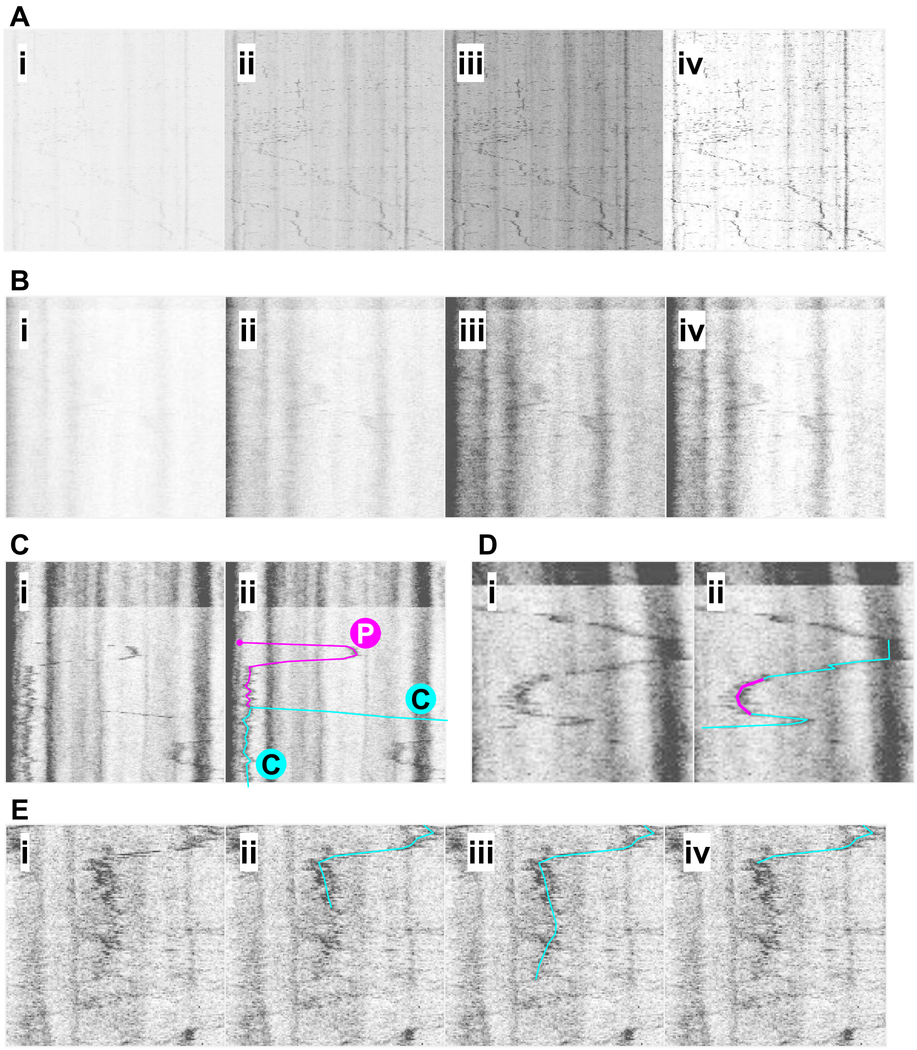

Figure S2: Kymogram contrast enhancement, thresholding, and puncta selection. (A) An example of axonal kymogram processing, where (i) is the raw image, (ii) is following automatic brightness/contrast adjustment, (iii) is following manual brightness/contrast adjustment, and (iv) is following thresholding. (B) A segment of dendritic kymogram is undergoing the same processing as (A). (C) Kymogram parent trajectories P that appear to merge or split into children C trajectories are each recorded as distinct trajectories. In (ii), one P (magenta) splits into two Cs (cyan), for a total of three trajectories. (D) If puncta appear to oscillate and the specific path cannot be resolved (i), trajectories are traced through the center of the oscillations (magenta segment) (ii). (E) To eliminate subjectivity in puncta trajectories that appear or fade away, immobile segments of trajectories are trimmed before and after mobile segments. For the disappearing trajectory shown in (i), both (ii) and (iii) would yield the same trajectory (iv) post trajectory trimming.

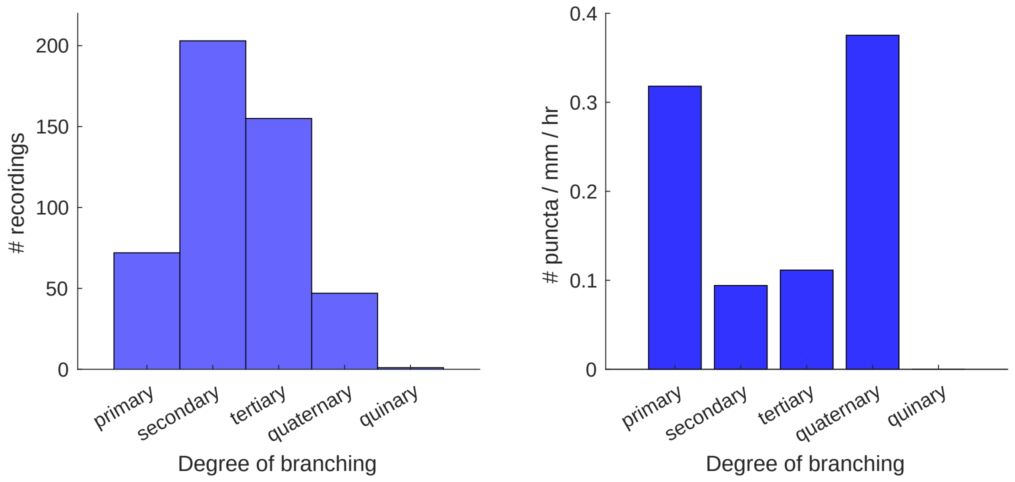

Figure S3: *Left:* Histogram depicting spread of dendritic recordings by degree of branching. Primary indicates the apical dendrite. *Right:* Degree of branching has no strong correlation with puncta frequency.

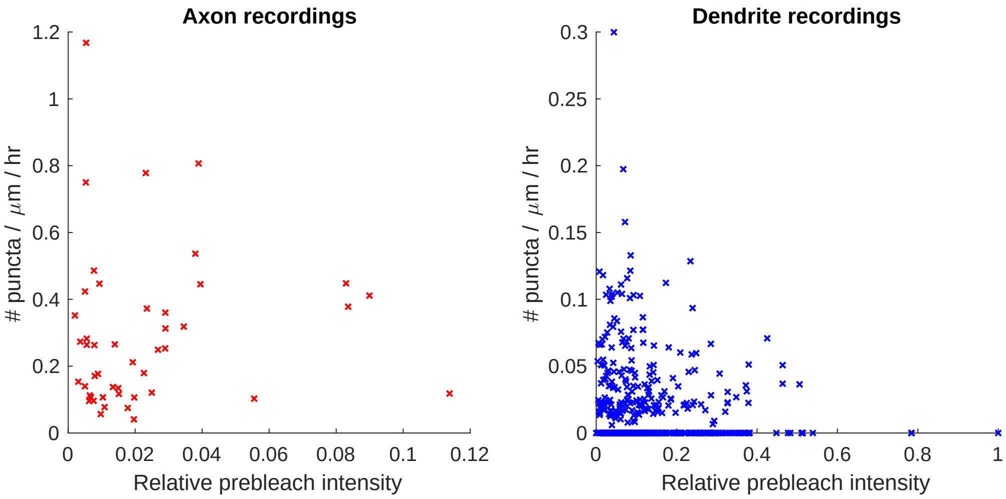

Figure S4: To ensure that puncta appearance or visibility is not an artifact of fluorescence intensity, we plot puncta frequency standardized by neurite length and recording duration versus average prebleach fluorescence intensity for recordings of all axons (*left*) and dendrites (*right*). Neither neurite population shows a strong correlation.

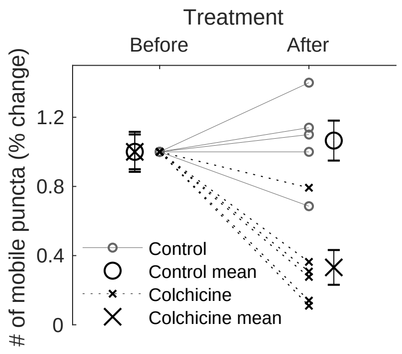

Figure S5: Percent change in number of mobile puncta following administration of microtubule disrupter (colchicine) compared to control (DMSO) during live recording.

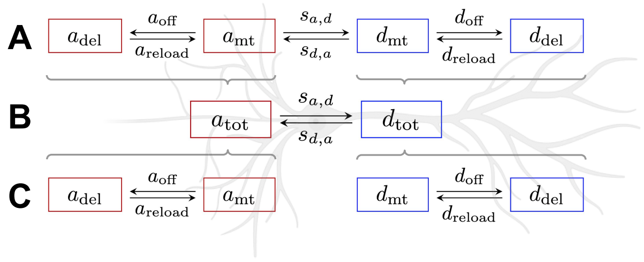

Figure S6: Lumped models of cargo distribution. (A): Mass action model of cargo transit on microtubules (*mt*) and delivery (*del*) in axons (*a*) and dendrites (*d*). (B): Simulation of (A) requires estimation of inter-neurite transit rates $s_{a,d}$ and $s_{d,a}$ using experimental constraints for total (*tot*) cargo. (C): Simulation of (A) requires estimation of cargo offloading (*off*) and reloading (*reload*) rates using experimental constraints for *mt* and *del*.

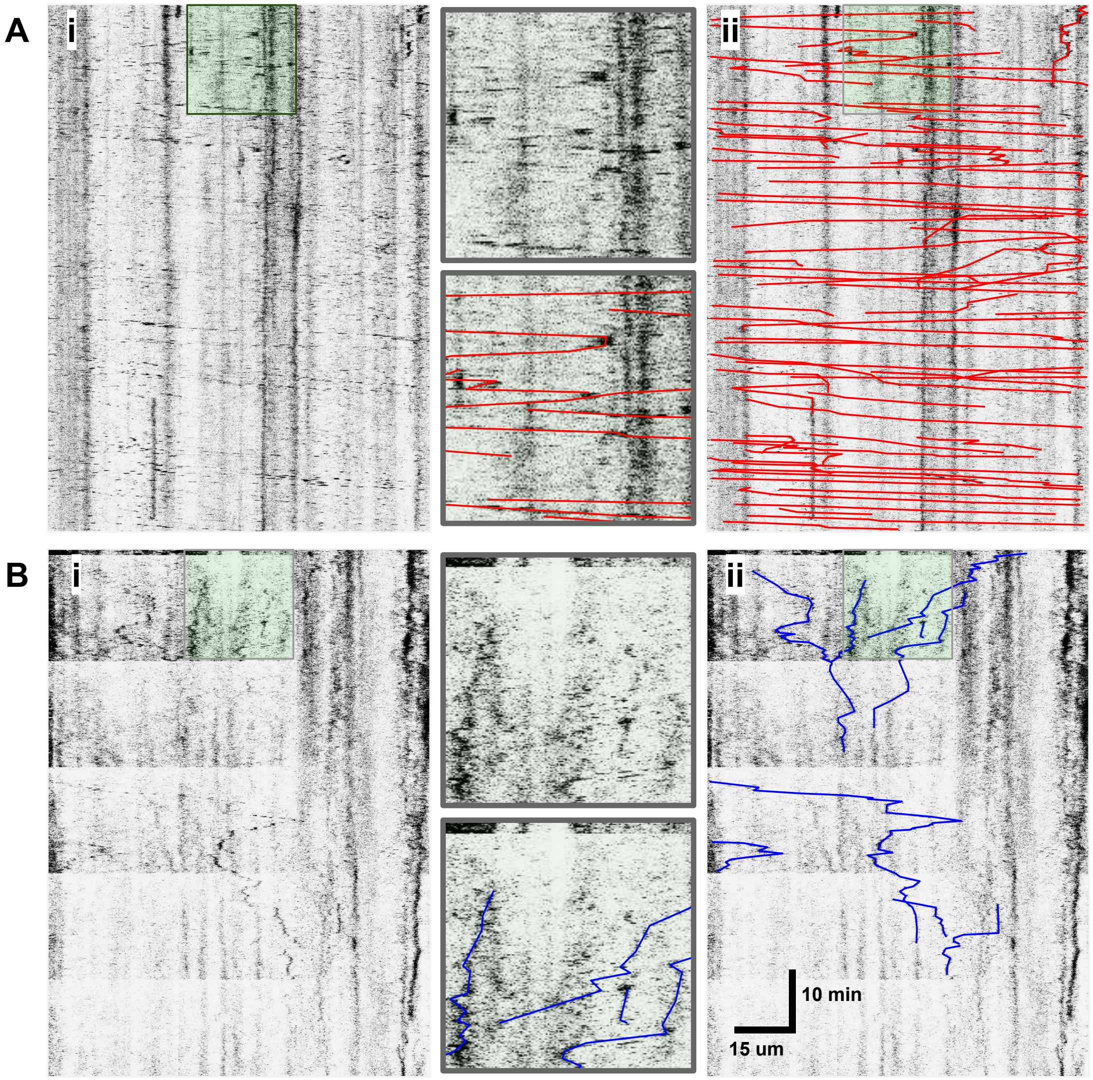

Figure S7: Kv4.2 trafficking is qualitatively different in axons and dendrites (A): Kymograms depicting characteristic axon trajectories (i) with puncta tracing overlaid in (ii). Insets correspond to regions highlighted in green. (B): Same as (A) for characteristic dendrite trajectories.

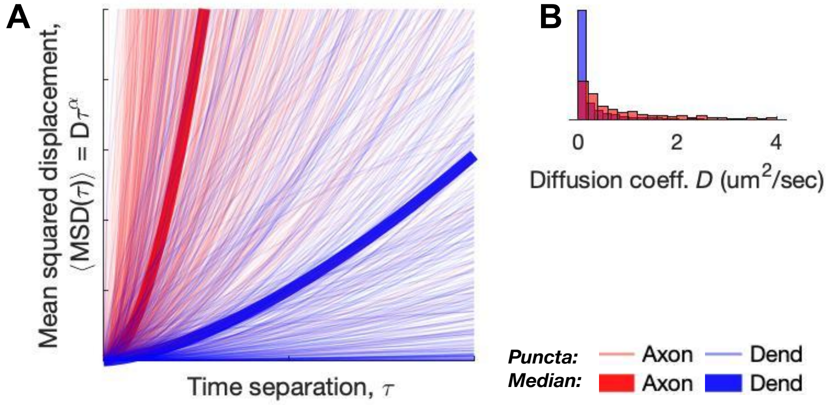

Figure S8: Result of curve fitting for mean squared displacement (MSD) versus time separation (τ), revealing higher degree of superdiffusivity in axons compared to dendrites (A). See details in Methods. Each thin line corresponds to one trajectory fit to $\mathrm{MSD}(\tau) = D\tau^{\alpha}$, where D is the diffusion coefficient, τ is the difference between two time points, and α is the diffusivity coefficient. $\alpha = 1$ is normal diffusion, $\alpha < 1$ is sub-diffusion, and $\alpha > 1$ is superdiffusion. Bold curves indicate the medians of the axonal and dendritic populations. Histogram show distribution for diffusion coefficient D (B).

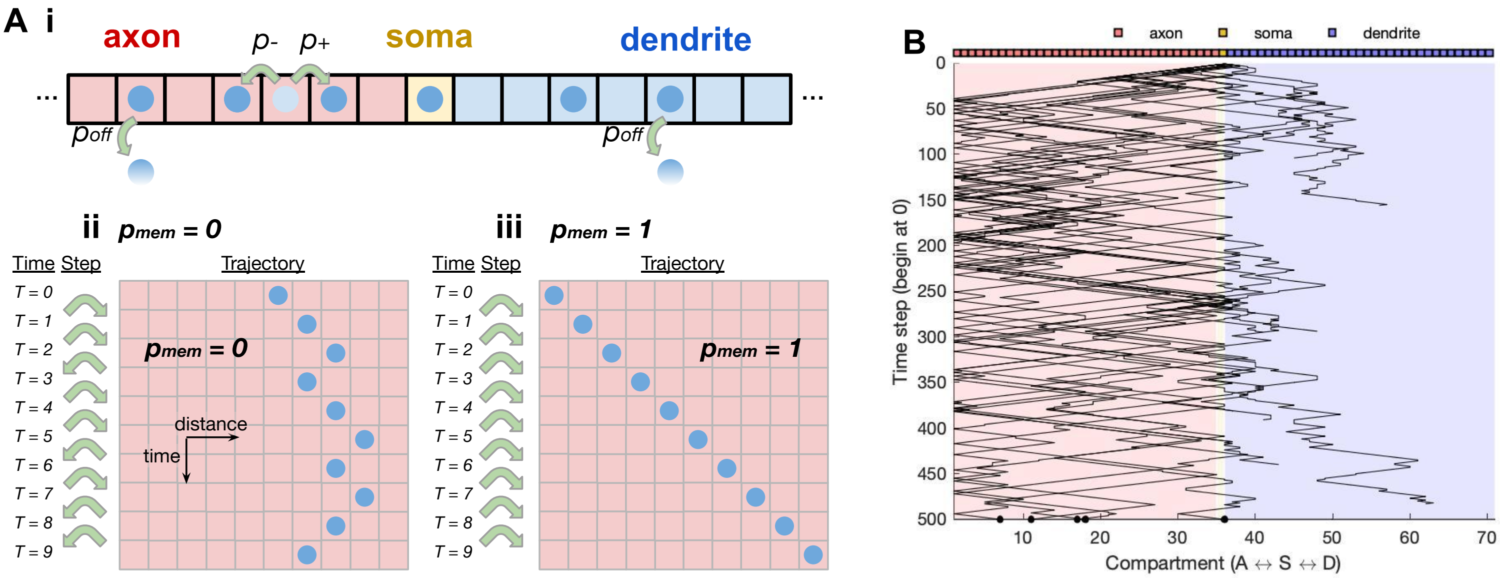

Figure S9: Model of modified random walk. (A): Setup of stochastic simulations along linear multi-compartment model (axon-soma-dendrite), with left/right jump and offloading rates depicted in (i). (ii) and (iii) depict extreme cases for memory parameter p_{mem}. (B): For demonstration of the stochastic model, 10 puncta are simulated over 500 time steps with $p^a_{\mathrm{off}} < p^d_{\mathrm{off}}$ and $p^a_{\mathrm{mem}} > p^d_{\mathrm{mem}}$. The resulting simulated trajectories visually compare to the experimentally-obtained kymograms.
